# A Unified Treatment of the Relationship Between Ligand Substituents and Spin State in a Family of Iron(II) Complexes

**DOI:** 10.1002/anie.201600165

**Published:** 2016-03-01

**Authors:** Laurence J. Kershaw Cook, Rafal Kulmaczewski, Rufeida Mohammed, Stephen Dudley, Simon A. Barrett, Marc A. Little, Robert J. Deeth, Malcolm A. Halcrow

**Affiliations:** ^1^ School of ChemistryUniversity of LeedsLeedsLS2 9JTUK; ^2^ Inorganic Computational Chemistry GroupDepartment of ChemistryUniversity of WarwickCoventryCV4 7ALUK; ^3^ Department of ChemistryUniversity of Bath, Claverton DownBathBA2 7AYUK; ^4^ Department of ChemistryUniversity of LiverpoolCrown StreetLiverpoolL69 7ZDUK; ^5^ School of ChemistryUniversity of EdinburghJoseph Black BuildingDavid Brewster RoadEdinburghEH9 3FJUK

**Keywords:** density functional calculations, iron, N ligands, spin state, substituent effects

## Abstract

The influence of ligands on the spin state of a metal ion is of central importance for bioinorganic chemistry, and the production of base‐metal catalysts for synthesis applications. Complexes derived from [Fe(bpp)_2_]^2+^ (bpp=2,6‐di{pyrazol‐1‐yl}pyridine) can be high‐spin, low‐spin, or spin‐crossover (SCO) active depending on the ligand substituents. Plots of the SCO midpoint temperature (*T* ${}_{1/2}$ ) in solution vs. the relevant Hammett parameter show that the low‐spin state of the complex is stabilized by electron‐withdrawing pyridyl (“X”) substituents, but also by electron‐donating pyrazolyl (“Y”) substituents. Moreover, when a subset of complexes with halogeno X or Y substituents is considered, the two sets of compounds instead show identical trends of a small reduction in *T* ${}_{1/2}$ for increasing substituent electronegativity. DFT calculations reproduce these disparate trends, which arise from competing influences of pyridyl and pyrazolyl ligand substituents on Fe‐L σ and π bonding.

The ability of first‐row transition ions to adopt different spin states in strong or weak ligand fields is of great importance to their catalysis and reactivity.^1^, ^2^, ^3^ For example, fundamental mechanistic steps in biological and synthetic oxidation catalysis involve a change in spin state at an iron catalyst center, described as two‐state reactivity.^3^ Catalysts with different resting spin states follow different pathways through these two‐state processes, leading to altered reactivity and product distributions.^4^ Similar considerations also apply for “base‐metal” catalysts for organometallic reactions,^5^ which give access to high‐spin active species with different reactivity patterns compared to conventional precious‐metal catalysts.^6^, ^7^ Another consequence of spin‐state dichotomy is the phenomenon of spin crossover (SCO), where a molecular or framework compound exhibits a transition between high‐ and low‐spin states under a physical stimulus.^8^, ^9^ SCO compounds have been developed into versatile molecular switches for molecular materials chemistry and nanoscience.^9^, ^10^

The relationship between chemical structure and spin state is central to these phenomena.^2^, ^11^ A sterically crowded ligand sphere generally leads to high‐spin complexes.^12^ However, the effect of ligand electronic character on metal‐ion spin state is less clear‐cut, with electron‐withdrawing substituents being reported to stabilize either the low‐spin^13^, ^14^, ^15^, ^16^ or the high‐spin state^17^, ^18^ in different series of compounds. While the literature includes data from solution and the solid‐state, these effects are best quantified by solution measurements which determine a complex's spin state in the absence of crystal‐packing effects or any other influences from a rigid solid lattice.^19^ We report herein a comprehensive study to resolve this contradiction, through a survey of twenty‐five complexes from the [Fe(bpp^X,Y^)_2_]^2+^ family (bpp^X,Y^=a 2,6‐di(pyrazol‐1‐yl)pyridine derivative; Scheme 1).^20^ Our results show that substituents at the X and Y sites have different, opposing effects on the iron‐atom spin state.

**Scheme 1:**
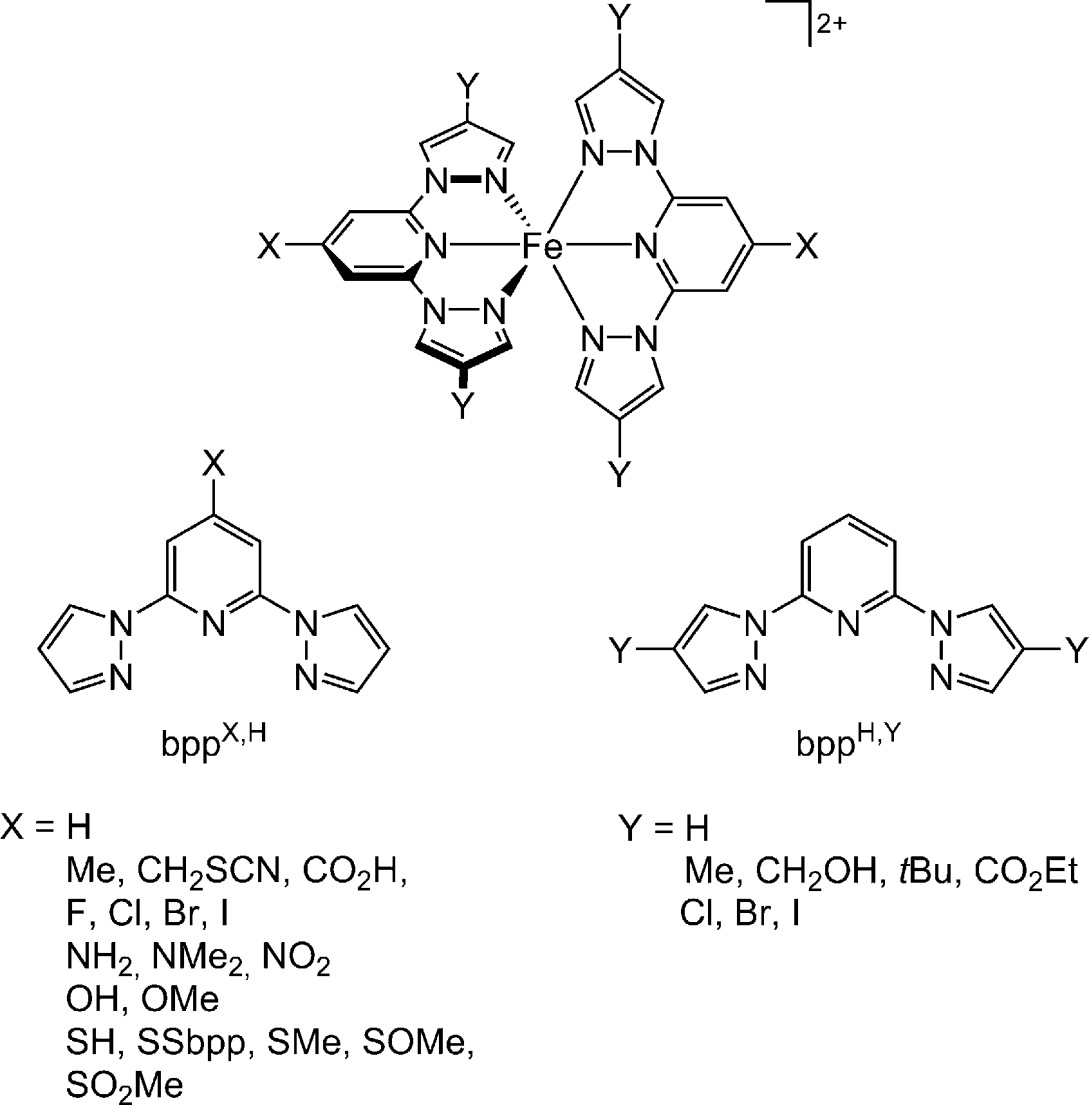
Different substitution patterns of [Fe(bpp)_2_]^2+^ (top), and the different bpp^X,Y^ ligands referred to in this study (bottom).

The spin states of these complexes were measured in solution by the variable‐temperature Evans method,^21^ in (CD_3_)_2_CO or CD_3_NO_2_ depending on their solubility (Figure 1). Our use of different weakly interacting solvents should cause only small perturbations to the data.^22^ The complexes with X=NH_2_ and NMe_2_ remain high‐spin within experimental error over the liquid range of the solvent. All the other complexes exhibit SCO, although the midpoint temperature of the transition (*T* ${}_{1/2}$ ) varies from 158 K (X=OMe)≤*T* ${}_{1/2}$ ≤305 K (X=NO_2_).^23^ Where they could be derived, thermodynamic parameters for these equilibria are mostly similar to other [Fe(bpp^X,Y^)_2_]^2+^ complexes.^20^, ^23^ However, higher Δ*H* and Δ*S* values for [Fe(bpp${}^{\mathrm{CO}{}_{2}H,H}$ )_2_]^2+^ and [Fe(bpp${}^{\mathrm{SO}{}_{2}\mathrm{Me},H}$ )_2_]^2+^ imply that ligand‐dissociation equilibria in those complexes may be occurring, promoted by the nucleophilic carboxylic and sulfoxide substituents. Since ligand dissociation only occurs in the labile high‐spin state of a complex, as a pre‐equilibrium to SCO, it will have little effect on *T* ${}_{1/2}$ .^19^, ^22^

**Figure 1:**
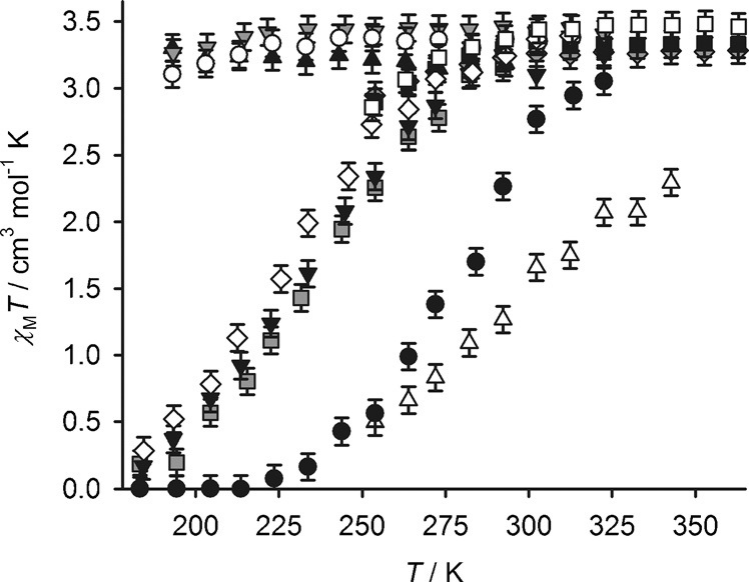
Solution‐phase magnetic susceptibilty data: [Fe(bpp^OH,H^)_2_][BF_4_]_2_ (○); [Fe(bpp^OMe,H)^_2_][PF_6_]_2_ (▼); [Fe(bpp${}^{\mathrm{NH}{}_{2},H}$ )_2_][BF_4_]_2_ (▲); [Fe(bpp^Me,H^)_2_][BF_4_]_2_ (□); [Fe(bpp^F,H^)_2_][BF_4_]_2_ (⧫); [Fe(bpp^pz,H^)_2_][BF_4_]_2_ (▪); [Fe(bpp^Cl,H^)_2_][BF_4_]_2_ (◊); [Fe(bpp${}^{\mathrm{Br},H{}_{2}}$ ][BF_4_]_2_ (▼); [Fe(bpp^I,H^)_2_][BF_4_]_2_ (▒); [Fe(bpp${}^{\mathrm{CO}{}_{2}H,H}$ )_2_][BF_4_]_2_ (•); [Fe(bpp${}^{\mathrm{NO}{}_{2},H}$ )_2_][BF_4_]_2_ (▵).^23^

Plots of *T* ${}_{1/2}$ versus the substituent electronegativity (*χ* ^P^ 24) for [Fe(bpp^X,H^)_2_]^2+^ and [Fe(bpp^H,Y^)_2_]^2+^ show identical correlations for substituents with weak π‐bonding character (X, Y=halogen and SH; Figure 2). Within this series, electronegative substituents lower *T* ${}_{1/2}$ to a small extent, so less electron‐rich X and Y groups slightly stabilize the high‐spin state. That is consistent with basic ligand‐field arguments. However, simple X and Y substituents with π‐bonding resonance properties (X, Y=CH_3_, NH_2_, and OH) deviate strongly from this relationship. That implies metal–ligand π bonding must contribute to the spin states of these complexes.

**Figure 2:**
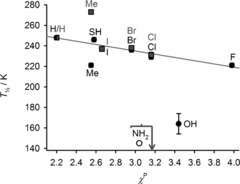
Plot of *T* ${}_{1/2}$ versus the substituent electronegativity (*χ* ^P^) for [Fe(bpp^X,H^)_2_]^2+^ (•) and [Fe(bpp^H,Y^)_2_]^2+^ (▪) complexes with simple heteroatom X and Y substituents.^23^ *T* ${}_{1/2}$ for X=NH_2_ (○) represents an upper limit for that measurement, since the complex is fully high‐spin over the liquid range of the solvent. The line shows the best fit correlation (*R* ^2^=0.91), omitting the X/Y=Me, OH and NH_2_ datapoints.

Resonance effects for ligand “X” substituents are accounted for by the *σ*_p_ Hammett parameter.^25^ A plot of *T* ${}_{1/2}$ versus *σ*_p_ for [Fe(bpp^X,H^)_2_]^2+^ contains some scatter, particularly around *σ*_p_≈0, but shows a positive linear correlation (Figure 3, top). That is, more electron‐withdrawing pyridyl X substituents stabilize the low‐spin state of [Fe(bpp^X,H^)_2_]^2+^. This result is consistent with previous studies of complexes with pyridyl donor ligands,^14^, ^15^, ^16^ but it is the opposite trend to the electronegativity plot (Figure 2). An improved correlation is found when *T* ${}_{1/2}$ is plotted against *σ*_p_^+^, a modified Hammett parameter accounting for conjugation of the ligand substitutents with a positively charged reaction center (Figure 3, bottom).^25^ Hence, these data appear to be influenced by π bonding between the Lewis acidic Fe^2+^ ion and the ligand pyridyl donors. In contrast, a plot of *T* ${}_{1/2}$ for [Fe(bpp^H,Y^)_2_]^2+^ versus the relevant substituent Hammett parameter (σ_M_ 25) shows the opposite trend from the [Fe(bpp^X,H^)_2_]^2+^ series. That is, more electron‐withdrawing pyrazolyl substituents stabilize the high‐spin state in [Fe(bpp^H,Y^)_2_]^2+^ derivatives, even when substituent resonance effects are included (Figure 4). Such a dependence of *T* ${}_{1/2}$ on the positioning of ligand substituents, in the absence of any steric influence, has not been noted before.

**Figure 3:**
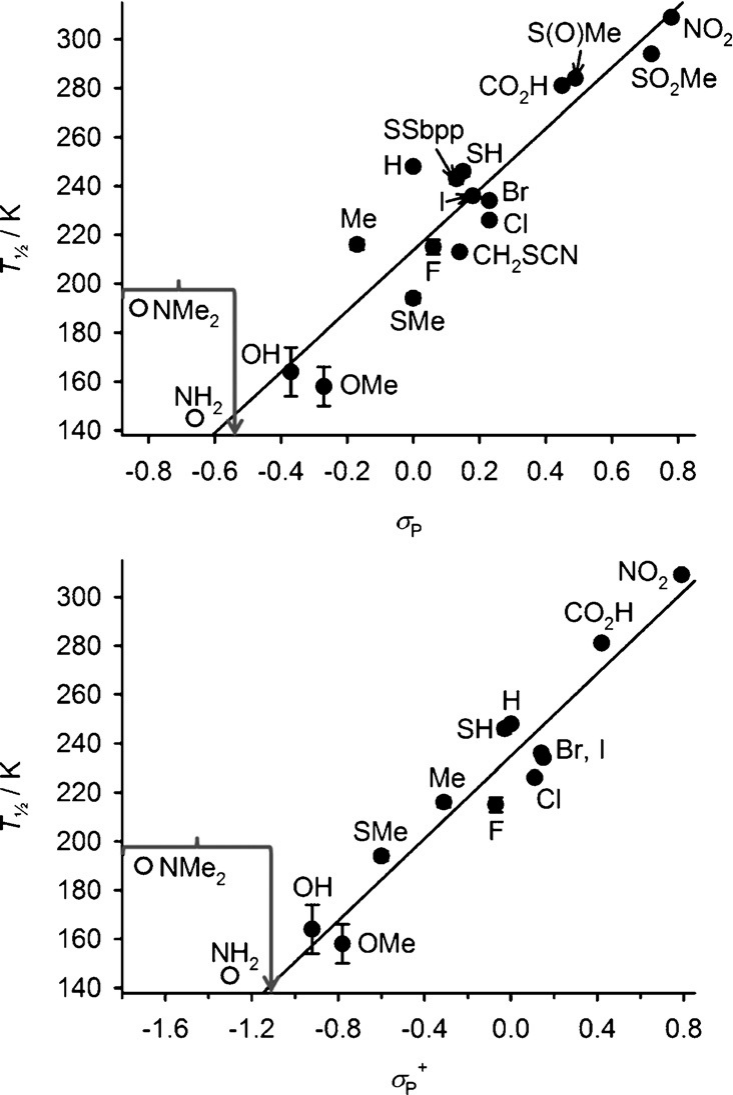
Plots of *T* ${}_{1/2}$ for [Fe(bpp^X,H^)_2_]^2+^ versus the X substituent Hammett parameters *σ*_P_ (top) and *σ*_P_^+^ (bottom; Table S1 in the Supporting Information).^23^ Error bars are mostly smaller than the symbols on the graph. The lines show the best fit correlation (*R* ^2^=0.86 [top] and 0.92 [bottom]), omitting the X=NH_2_ and NMe_2_ datapoints (○) which represent the upper limits for those *T* ${}_{1/2}$ measurements.

**Figure 4:**
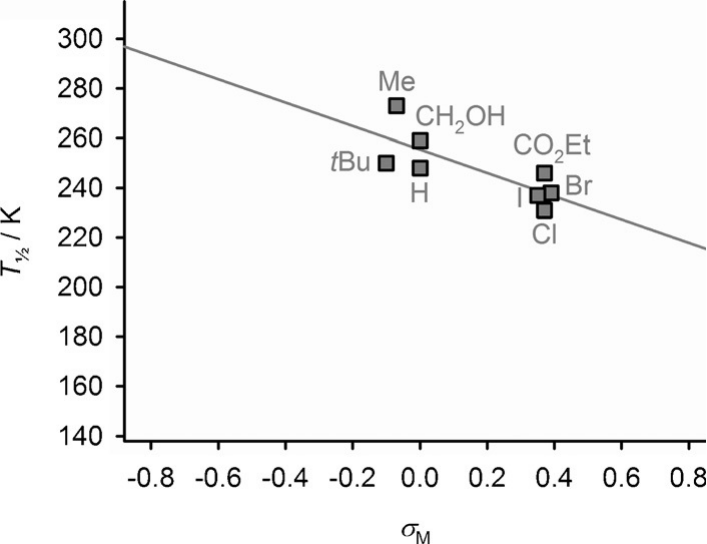
Plot of *T* ${}_{1/2}$ versus the Y substituent Hammett parameters *σ*_M_ for [Fe(bpp^H,Y^)_2_]^2+^ complexes with different Y substituents.^23^ Error bars are shown, but are smaller than the symbols on the graph. The line shows the best fit correlation (*R* ^2^=0.61). The graph is drawn for the same range as Figure 3 (top), to aid comparison.

This question was probed by density functional (DFT) calculations of [Fe(bpp^X,Y^)_2_]^2+^ using the BP86 functional. The correlation between the measured *T* ${}_{1/2}$ and the computed difference between the high‐spin and low‐spin total energies, Δ*E*_rel_(HS‐LS), is very good despite the relatively simplistic computational method used,^26^ with a *R* ^2^ correlation coefficient of 0.79.^23^ The agreement between Δ*E*_rel_(HS‐LS) and the X or Y substituent Hammett parameter is moderate when all the compounds are plotted together, but improves when [Fe(bpp^X,H^)_2_]^2+^ and [Fe(bpp^H,Y^)_2_]^2+^ are considered separately (Figure 5). Hence, the calculations have captured the spin‐state behavior of the two sets of compounds.

**Figure 5:**
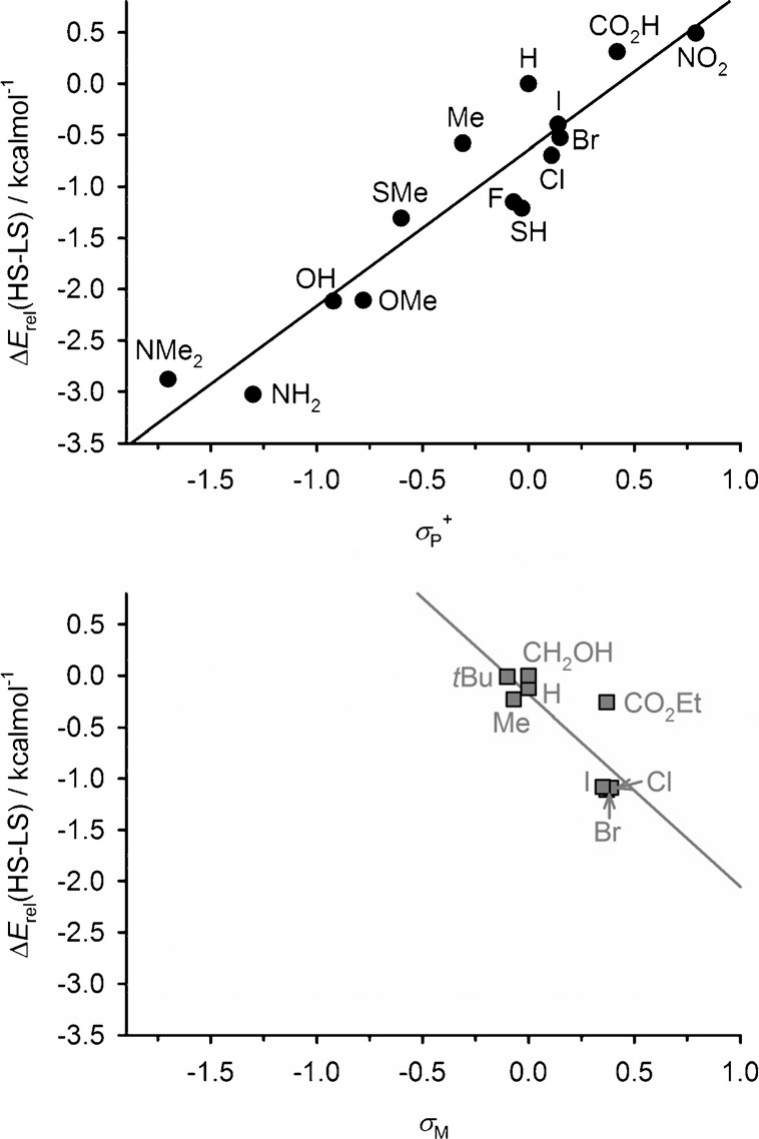
Plot of the relevant substituent Hammett parameter vs. the computed energy difference between the high‐ and low‐spin states relative to X=Y=H [Δ*E*_rel_(HS‐LS)], for: [Fe(bpp^X,H^)_2_]^2+^ (top, •) and [Fe(bpp^H,Y^)_2_]^2+^ (bottom, ▒).^23^ The graphs are plotted to the same scale to aid comparison, and the lines show the best fit correlations (*R* ^2^=0.89 [top] and 0.67 [bottom]^28^).

The σ and π contributions to Fe−L bonding for each bpp^X,Y^ ligand were quantified by considering the d‐orbital energies of the low‐spin compounds. Electron‐withdrawing X or Y substituents lower the energy of all the metal d‐orbitals (Figure 6), but the effect is 2–3 times greater for Y substituents than for X substituents since there as twice as many Y substituents as X groups in a [Fe(bpp^X,Y^)_2_]^2+^ molecule. The X substituents in [Fe(bpp^X,H^)_2_]^2+^ have a greater effect on the averaged *t*_2g_ orbital energies than on the *e*_g_ orbitals, from the slopes of their least squares correlations (Figure 6). In contrast, Y substituents in [Fe(bpp^H,Y^)_2_]^2+^ have a much larger influence on the averaged *e*_g_ orbital energies than on the *t*_2g_ energies (Figure 6).^27^

**Figure 6:**
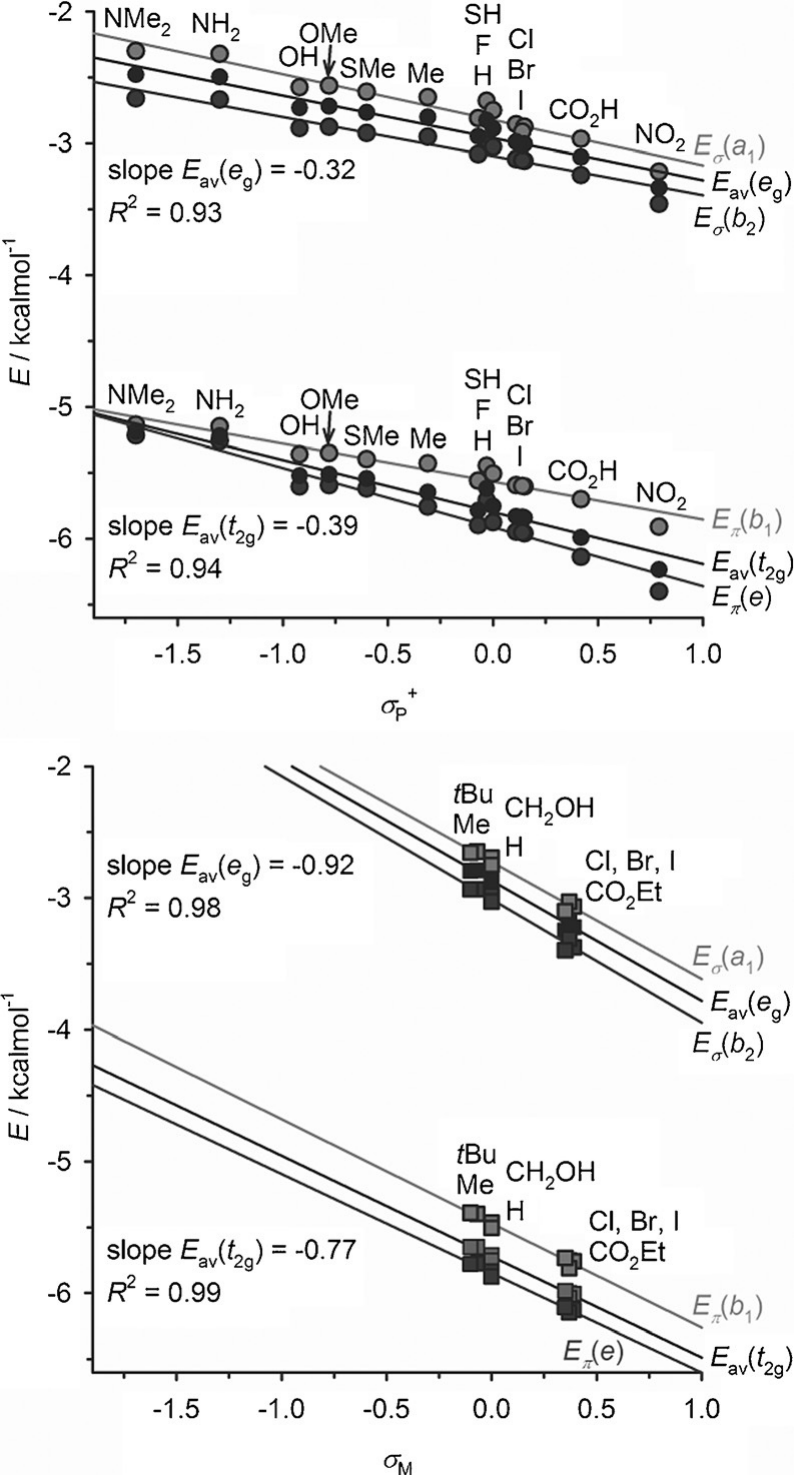
Plot of the relevant substituent Hammett parameter versus the computed d‐orbital energies for [Fe(bpp^X,H^)_2_]^2+^ (top, circles) and [Fe(bpp^H,Y^)_2_]^2+^ (bottom, squares).^23^, ^27^ The average orbital energies of the *t*_2g_ and *e*_g_ subshells are also shown, along with their best fit correlations and slopes.

The relationship between *T* ${}_{1/2}$ and the bpp^X,Y^ ligand is a competition between Fe−L σ‐ and π‐bonding effects. Electron‐withdrawing substituents inductively lower the energy of the bpp lone pairs, weakening the σ ligand field and thus stabilizing the high‐spin state. Conversely, electron‐withdrawing substituents also reduce the energy of the bpp^X,Y^ π* MOs, which increases the ligand field by strengthening Fe→bpp π backbonding and favors the low‐spin state. Fe−L π‐bonding effects dominate in the [Fe(bpp^X,H^)_2_]^2+^ series, where electron‐withdrawing substituents stabilize the *t*_2g_ orbital manifold more strongly than the *e*_g_, thus increasing the ligand field and raising *T* ${}_{1/2}$ . In contrast, the spin state of the [Fe(bpp^H,Y^)_2_]^2+^ family is controlled by Fe−L σ bonding, since electron‐withdrawing Y substituents stabilize the *e*_g_ orbitals more strongly, promoting the high‐spin state and lowering *T* ${}_{1/2}$ .

When complexes with halogen X and Y substituents are considered separately, the stabilization of *E*_av_(*e*_g_) by electron‐withdrawing substituents is approximately 25 % greater than *E*_av_(*t*_2g_) for both sets of complexes.^23^ Thus, electronegative halogen X and Y groups both reduce *T* ${}_{1/2}$ , and the essentially identical *T* ${}_{1/2}$ values shown by [Fe(bpp^X,H^)_2_]^2+^ and [Fe(bpp^H,Y^)_2_]^2+^ when X, Y=a halogen (Figure 2) are also supported by this computational study, despite being contrary to the rest of the data.^17^

These results reconcile the differing conclusions from earlier studies. Electron‐withdrawing substitutents indeed stabilize either the low‐spin^13^, ^14^, ^15^, ^16^ or the high‐spin state^17^, ^18^ of a complex, depending on their position in the molecule and on which types of substituent are considered. The relationship between ligand design and metal‐ion spin state is a fine balance between opposing M−L σ‐ and π‐bonding effects. Rational design of a complex with defined spin‐state properties for SCO, catalysis, or other applications requires consideration of all these aspects of the metal–ligand interaction.

## Supporting information

As a service to our authors and readers, this journal provides supporting information supplied by the authors. Such materials are peer reviewed and may be re‐organized for online delivery, but are not copy‐edited or typeset. Technical support issues arising from supporting information (other than missing files) should be addressed to the authors.

Supplementary
