# Supplementary material for: A Unified Treatment of the Relationship Between Ligand Substituents and Spin State in a Family of Iron(II) Complexes
Source: Angew Chem Int Ed Engl. 2016 Mar 1;55(13):4327–31. doi: 10.1002/anie.201600165 (PMC4804750; doi:10.1002/anie.201600165)
Supplement: Supplementary file 1 — Supplementary [file ANIE-55-4327-s001.pdf]

Angewandte Chemie
Eine Zeitschrift der Gesellschaft Deutscher Chemiker

Supporting Information

A Unified Treatment of the Relationship Between Ligand Substituents and Spin State in a Family of Iron(II) Complexes

Laurence J. Kershaw Cook, Rafal Kulmaczewski, Rufeida Mohammed, Stephen Dudley, Simon A. Barrett, Marc A. Little, Robert J. Deeth, and Malcolm A. Halcrow**

anie_201600165_sm_miscellaneous_information.pdf

(1) Solution phase magnetic susceptibility data and computational details

		Page
**Experimental details – solution magnetic measurements**		3
**Computational details**		4
**Table S1**	Solution phase spin-crossover data for $[Fe(bpp^{X,H})_2]^{2+}$.	5
**Table S2**	Solution phase spin-crossover data for $[Fe(bpp^{H,Y})_2]^{2+}$.	6
**Figure S1**	New solution phase magnetic susceptibility data for the compounds in this work.	7
**Figure S2**	Plots of the measured $T_{½}$ values for $[Fe(bpp^{X,H})_2]^{2+}$ and $[Fe(bpp^{H,Y})_2]^{2+}$ *vs.* the basic pK_a of the corresponding 4-substituted heterocycle.	9
**Table S3**	Computed energy difference between the high-spin and low-spin forms of the complexes.	10
**Figure S3**	Plot of the experimentally measured $T_{½}$ *vs* the computed ΔE_{rel}(HS-LS) for $[Fe(bpp^{X,H})_2]^{2+}$ and $[Fe(bpp^{H,Y})_2]^{2+}$.	11
**Table S4**	Calculated composition of the metal *d*-orbitals in $[Fe(bpp^{H,H})_2]^{2+}$.	12
**Table S5**	Computed *d*-orbital energies in the low-spin forms of the complexes.	13
**Figure S4**	Plot of the relevant substituent Hammett parameter *vs* the computed *d*-orbital energies for $[Fe(bpp^{X,H})_2]^{2+}$ and $[Fe(bpp^{H,Y})_2]^{2+}$ with X, Y = H or halogen only.	14
**Figure S5**	Computed energy-minimized structures of the low-spin forms of a selection of the complexes in this work.	15
**Table S6**	Atomic coordinates for the computed, energy-minimized structures of the $[Fe(bpp^{X,H})_2]^{2+}$ and $[Fe(bpp^{H,Y})_2]^{2+}$ complexes in their high-spin and low-spin states.	17

(2) Synthetic, crystallographic and solid state magnetic data

		Page
**Scheme S1**	Syntheses of the new bpp^{X,H} ligands in this work.	42
**Experimental details of the ligand syntheses**		42
**Experimental details of the synthesis of the complexes**		44
**Figure S6**	^{1}H and ^{13}C NMR spectra of bpp^{NMe2,H}.	45
**Figure S7**	^{1}H and ^{13}C NMR spectra of bpp^{NO2,H}.	46
**Figure S8**	^{1}H and ^{13}C NMR spectra of bpp^{F,H}.	47
**Figure S9**	^{1}H and ^{13}C NMR spectra of bpp^{OMe,H}.	48
**Figure S10**	^{1}H and ^{13}C NMR spectra of bpp^{H,tBu}.	49
**Experimental details of the single crystal structure analyses**		50-53
**Table S7**	Experimental data for the ligand crystal structure determinations in this work.	51
**Table S8**	Experimental data for the metal complex structure determinations in this work.	52
**Other techniques**		54
**Figure S11**	Views of the molecules in the ligand crystal structures bpp^{NO2,H} and bpp^{H,tBu}.	55
**Table S9**	Spin-state behavior of the new complexes in this work in the solid state.	56
**Figure S12**	View of the hydrogen bonded moieties in [Fe(bpp^{NH2,H})$_2$[BF$_4$]$_2$·3MeNO$_2$ and [Fe(bpp^{NH2,H})$_2$[ClO$_4$]$_2$·3MeNO$_2$.	57
**Table S10**	Selected bond lengths and angles in the crystal structures of [Fe(bpp^{NH2,H})$_2$][BF$_4$]$_2$·3MeNO$_2$ and [Fe(bpp^{NH2,H})$_2$][ClO$_4$]$_2$·3MeNO$_2$.	58
**Table S11**	Hydrogen bond parameters for the crystal structures of [Fe(bpp^{NH2,H})$_2$]$^{2+}$ complex salts.	58
**Figure S13**	Partial packing diagram of [Fe(bpp^{NH2,H})$_2$][BF$_4$]$_2$·3MeNO$_2$, showing the association of the complex cations into puckered hydrogen-bonded chains *via* a bridging BF$_4^-$ ion.	59
**Figure S14**	Packing diagram of [Fe(bpp^{NH2,H})$_2$][BF$_4$]$_2$·3MeNO$_2$.	60
**Figure S15**	Views of the complex dications in [Fe(bpp^{NMe2,H})$_2$][BF$_4$]$_2$·xH$_2$O, [Fe(bpp^{NMe2,H})$_2$][ClO$_4$]$_2$·xH$_2$O and [Fe(bpp^{OMe,H})$_2$][PF$_6$]$_2$.	61
**Table S12**	Selected bond lengths and angles in the crystal structures of [Fe(bpp^{NMe2})$_2$][BF$_4$]$_2$·xH$_2$O, [Fe(bpp^{NMe2})$_2$][ClO$_4$]$_2$·xH$_2$O and [Fe(bpp^{OMe,H})$_2$][PF$_6$]$_2$.	62
**Figure S16**	View of the complete formula unit in [Fe(bpp^{NMe2,H})$_2$][BF$_4$]$_2$·xH$_2$O (x = 0.29), showing the influence between the partial water site O(50) and the disordered anion.	63
**Figure S17**	View of the complex dications in [Fe(bpp^{F,H})$_2$][BF$_4$]$_2$ at 290 K and 150 K, and in [Fe(bpp^{Cl,H})$_2$][BF$_4$]$_2$.	64
**Table S13**	Selected bond lengths and angles in the crystal structures of the halogenated ligand complexes.	65
**Figure S18**	Packing diagrams of [Fe(bpp^{F,H})$_2$][BF$_4$]$_2$ in its high-spin and low-spin states.	66
**Figure S19**	Solid state magnetic susceptibility data for the new complexes in this work.	67

Experimental details – solution magnetic measurements

Magnetic susceptibility measurements in solution were obtained by Evans method using a Bruker DRX500 or Avance500 spectrometer operating at 500.13 MHz.[1] A diamagnetic correction for the sample,[2] and a correction for the variation of the density of the solvent with temperature,[3] were applied to these data. The spin-crossover midpoint temperatures from these data were derived by fitting the data to eq 1 and 2, where $n_{HS}(T)$ is the high-spin fraction of the sample at temperature T (Fig. S1).

$$\ln[(1- n_{HS}(T))/n_{HS}(T)] = (\Delta H/RT) - \Delta S/R \quad (1)$$

$$\Delta S = \Delta H/T_{½} \quad (2)$$

For data where $T_{½}$ is below the freezing point of the solvent, the restraint $18 \leq \Delta H \leq 22$ kJmol^{-1} was used to obtain a meaningful fit, and estimate $T_{½}$. This curve fitting was performed using *SIGMAPLOT*.[4]

[1] a) D. F. Evans, *J. Chem. Soc.* **1959**, 2003; b) E. M. Schubert, *J. Chem. Educ.* **1992**, *69*, 62.

[2] C. J. O'Connor, *Prog. Inorg. Chem.* **1982**, *29*, 203.

[3] a) J. C. Philip, H. B. Oakley, *J. Chem. Soc. Trans.* **1924**, *125*, 1189; b) W. A. Felsing, S. A. Durban, *J. Am. Chem. Soc.* **1926**, *48*, 2885.

[4] *SIGMAPLOT*, v. 8.02, SPSS Scientific Inc., Chicago IL, **2002**.

Experimental details – Computational study

All DFT calculations employed the *ORCA* program system, version 3.0.1.[5] All complexes were fully optimized using the Becke Perdew (*BP86*) functional[6,7] and a def2-SVP basis set.[8] The resolution of identity approximation was also used with a def2-SVP/J auxiliary basis. Low spin systems were treated spin restricted and high spin systems spin unrestricted. For the latter, the spin expectation value $<S^2>$ was of the order of 6.06 compared to the ideal value of 6. Although the theoretical justification within DFT of trusting the value of $<S^2>$ is debateable, we judge the spin contamination to be negligible.

To account for condensed phase effects, the optimizations were carried out in a polarizable continuum solvent using the conductor like screening model (*COSMO*)[9] implemented in *ORCA*. The solvent used was acetone. Default convergence criteria for the SCF and geometry were applied. Frequency calculations were carried out for the unsubstituted complex $[Fe(bpp^{H,H})_2]^{2+}$ which confirmed that both HS and LS forms were local minima. These structures were employed as the starting coordinates for substituted systems and no further frequency calculations were done. In common with our previous study of this type,[10] no corrections were made for zero point energies or dispersion and the analysis relies solely on the total electronic energies.

For substituents where multiple conformations are possible, the ones selected for this work are shown below (Fig. S5). Systematic conformational searches were not carried out. However, several conformations were calculated for CH_2SCN and CH_2OH, which showed minimal variation of the spin state and MO energy differences indicating significant error cancellations and that the particular choices of conformation should not significantly alter our conclusions.

After each structure minimization, a Loewden population analysis was performed in *ORCA*[5] using the following commands:

```
!RKS BP RI def2-SVP def2-SVP/J TightSCF SlowConv Cosmo(Acetone)
%Output
Print[P_ReducedOrbPopMO_L] 1
End
```

The five metal-based *d*-frontier orbitals were clearly identifiable in the resultant outputs, as containing *ca.* 65-70 % metal *d* character (Table S4). The atomic coordinates of all the minimized structures are listed in Table S6, while the *d*-orbital energies are summarized in Table S5. Plots correlating the spin state energies or orbital energies with substituent Hammett parameters were produced using *SIGMAPLOT*.[4]

[5] F. Neese, U. Becker, D. Ganiouchine, S. Koßmann, T. Petrenko, C. Riplinger, F. Wennmohs, *ORCA* 3.0.1 edn., Max Planck Institute for Chemical Energy Conversion, Mülheim an der Ruhr, **2014**.

[6] A. D. Becke, *Phys. Rev. A* **1988**, *38*, 3098.

[7] J. P. Perdew, W. Yue, *Phys. Rev. B* **1986**, *33*, 8800 and **1989**, *40*, 3399 [correction].

[8] A. Schaefer, H. Horn, R. Ahlrichs, *J. Chem. Phys.* **1992**, *97*, 2571.

[9] a) A. Klamt, V. Jones, *J. Chem. Phys.* **1996**, *105*, 9972; b) A. Klamt, *J. Phys. Chem.* **1995**, *99*, 2224;

c) A. Klamt, G. Schüürmann, *J. Chem. Soc., Perkin Trans. 2* **1993**, 799.

[10] B. J. Houghton, R. J. Deeth, *Eur. J. Inorg. Chem.* **2014**, 4573.

Table S1. Solution phase spin-crossover data for $[Fe(bpp^{X,H})_2]^{2+}$ (HS = fully high-spin over the liquid range of the solvent). Thermodynamic parameters are only quoted for equilibria that are fully resolved within the temperature range of the measurement.

X	χ_P	σ_P	σ_P^+	Solvent	$T_{½}$ / K	ΔH / kJmol^{-1}	ΔS / Jmol^{-1}K^{-1}	R^2	Ref
H	2.20	0	0	$(CD_3)_2CO$	248(1)	24.1	101	–	[13]
Me	2.55	–0.17	–0.31	CD_3NO_2	216(2)[a]	–	–	0.91	This work
CH_2SCN	–	0.14	–	CD_3CN^b	213(1)	–	–	–	[14]
CO_2H	–	0.45	0.42	$(CD_3)_2CO$	281(1)	40.9	146	0.88	This work
NH_2	3.04	–0.66	–1.30	$(CD_3)_2CO$	HS[b]	–	–	–	This work
NMe_2	–	–0.83	–1.70	CD_3NO_2	HS[b]	–	–	–	This work
NO_2	–	0.78	0.79	CD_3NO_2	309(2)	20.6	67	0.99	This work
pz (pyrazol-1-yl)	–	–	–	CD_3NO_2	215(3)[a]	–	–	0.71	This work
OH	3.44	–0.37	–0.92	$(CD_3)_2CO$	164(10)	–	–	0.53	This work
OMe	–	–0.27	–0.78	$(CD_3)_2CO$	158(8)	–	–	0.72	This work
F	3.98	0.06	–0.07	CD_3NO_2	215(4)[a]	–	–	0.93	This work
Cl	3.16	0.23	0.11	$(CD_3)_2CO$	226(1)	21.9	96	0.99	This work
Br	2.96	0.23	0.15	$(CD_3)_2CO$	234(1)	21.2	90	0.98	This work
I	2.66	0.18	0.14	$(CD_3)_2CO$	236(1)	23.2	98	0.97	This work
SH	2.58	0.15	–0.03	CD_3NO_2	246(2)	–	–	–	[15]
SSbpp	–	0.13[c]	–	CD_3NO_2	243(2)	–	–	–	[15]
SMe	–	0	–0.60	$(CD_3)_2CO$	194(2)	–	–	–	[16]
SOMe	–	0.49	–	$(CD_3)_2CO$	284(1)	23.0	81	–	[16]
SO_2Me	–	0.72	–	CD_3NO_2	294(1)	33.0	112	–	[16]
cis–CH=CHPh	–	–	–	$(CD_3)_2CO$	245(1)	19.4	79	–	[17]
cis–CH=CH(C_6H_4CN–4)	–	–	–	$(CD_3)_2CO$	259(1)	20.1	78	–	[17]
cis–CH=CH($C_6H_4NO_2$–4)	–	–	–	$(CD_3)_2CO$	261(1)	21.1	81	–	[17]

[a]$T_{½}$ derived by extrapolation below the freezing point of the solvent. [b]Compound remains fully high-spin within the liquid range of the solvent. [c] Hammett constant value is for R = SSMe.

The errors shown on $T_{½}$ are estimated errors, rather than standard deviations. χ_P is the Pauling electronegativity of the substituent heteroatom,[11] and Hammett parameters are taken from ref. [12]. R^2 is the correlation coefficient for the fits to the data in Fig. S1.

[11] L. Pauling, *J. Am. Chem. Soc.* **1932**, *54*, 3570.

[12] C. Hansch, A. Leo, R. W. Taft, *Chem. Rev.* **1991**, *91*, 165.

Table S2. Solution phase spin-crossover data for $[Fe(bpp^{H,Y})_2]^{2+}$. Details as for Table S1.

Y	χ_P	σ_M	Solvent	$T_{1/2}$ / K	ΔH / kJmol^{-1}	ΔS / Jmol^{-1}K^{-1}	R^2	Ref
H	2.20	0	$(CD_3)_2CO$	248(1)	24.1	101	–	[13]
Me	2.55	−0.07	CD_3NO_2	273(1)	26.4	96	–	[18]
CH_2OH	–	0	$(CD_3)_2CO$	259(2)	25.7	99	–	[18]
*t*Bu	–	−0.10	$(CD_3)_2CO$	251(1)	19.0	76	0.99	This work
CO_2Et	–	0.37	$(CD_3)_2CO$	246(1)	24.4	99	–	[18]
Cl	3.16	0.37	CD_3NO_2	231(3)[a]	–	–	–	[19]
Br	2.96	0.39	CD_3NO_2	238(2)[a]	–	–	–	[19]
I	2.66	0.35	$(CD_3)_2CO$	237(2)	19.2	80	0.93	This work

[a] $T_{1/2}$ derived by extrapolation below the freezing point of the solvent.

[13] J. M. Holland, J. A. McAllister, C. A. Kilner, M. Thornton–Pett, A. J. Bridgeman, M. A. Halcrow, *J. Chem. Soc. Dalton Trans.* **2002**, 548.

[14] M. Haryono, F. W. Heinemann, K. Petukhov, K. Gieb, P. Müller, A. Grohmann, *Eur. J. Inorg. Chem.* **2009**, 2136.

[15] L. J. Kershaw Cook, J. Fisher, L. P. Harding, M. A. Halcrow, *Dalton Trans.* **2015**, 44, 9417.

[16] L. J. Kershaw Cook, R. Kulmaczewski, S. A. Barrett, M. A. Halcrow, *Inorg. Chem. Front.* **2015**, *2*, 662.

[17] K. Takahashi, Y. Hasegawa, R. Sakamoto, M. Nishikawa, S. Kume, E. Nishibori, H. Nishihara, *Inorg. Chem.* **2012**, *51*, 5188.

[18] R. Pritchard, C. A. Kilner, S. A. Barrett, M. A. Halcrow, *Inorg. Chim. Acta* **2009**, *362*, 4365.

[19] R. Pritchard, H. Lazar, S. A. Barrett, C. A. Kilner, S. Asthana, C. Carbonera, J.–F. Létard, M. A. Halcrow, *Dalton Trans.* **2009,** 6656.

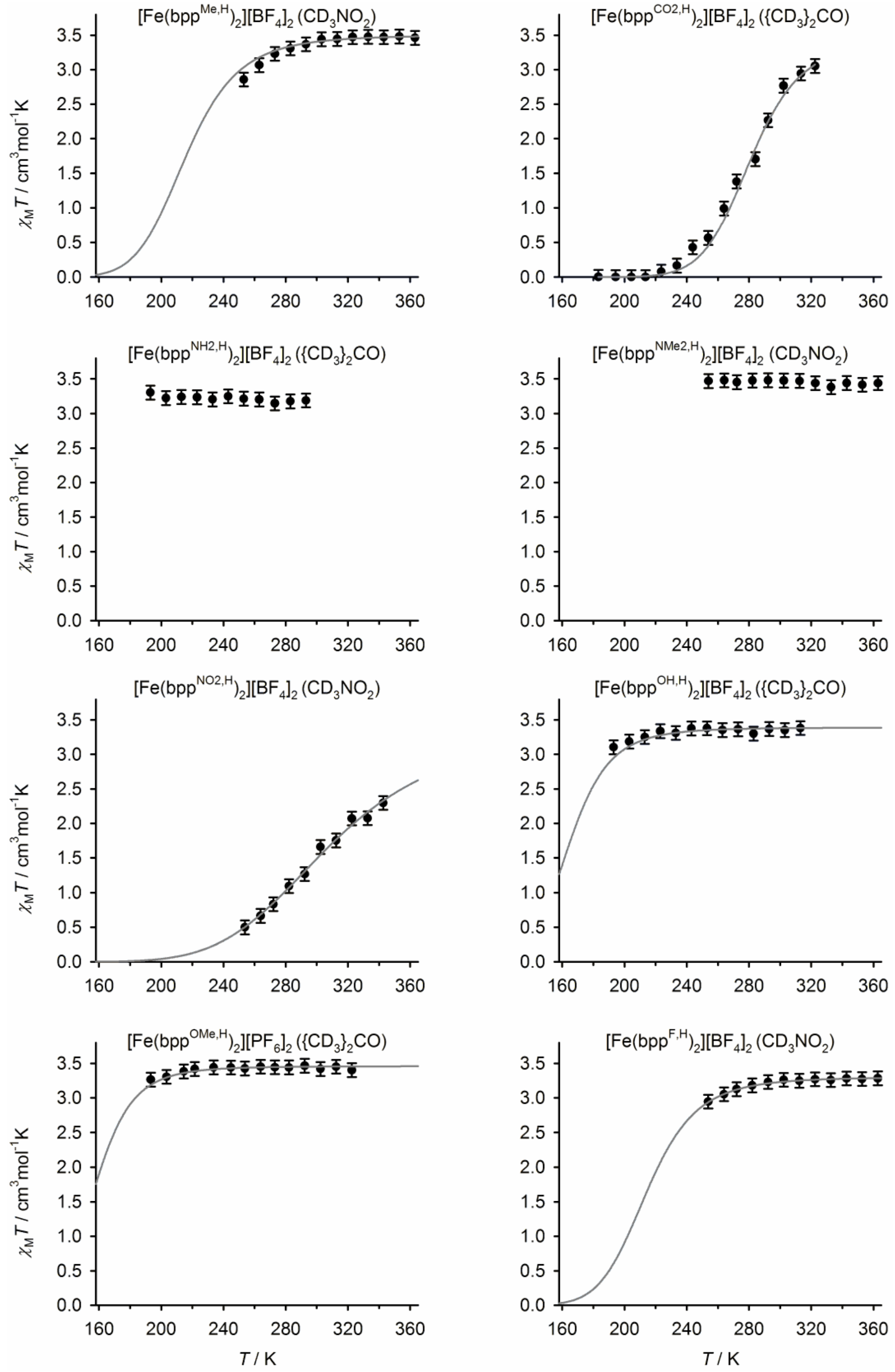

Fig. S1. New solution phase magnetic susceptibility data for the compounds in this work. The lines show the best fit of each curve to the equation $\ln K = \Delta H/RT - \Delta S/R$ where $\Delta S = \Delta H/T_{½}$ (Tables S1 and S2).

Some of these data are also included in Fig. 1 of the main article.

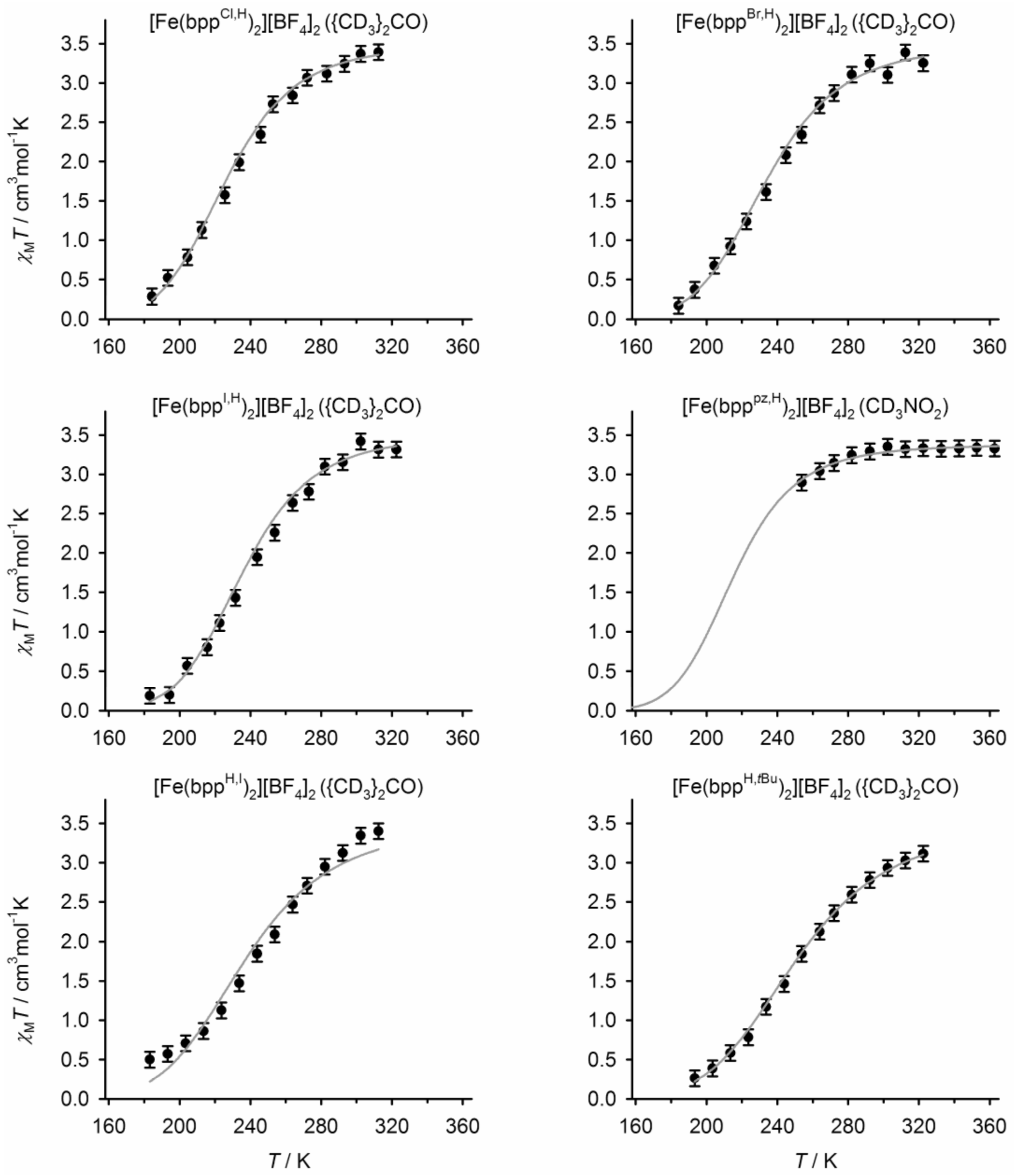

Fig. S1 continued.

No simulation of the data was undertaken for $[Fe(bpp^{NH2,H})_2][BF_4]_2$ and $[Fe(bpp^{NMe2,H})_2][BF_4]_2$, since there is no decrease in $\chi_M T$ at the lowest temperature measured to indicate the onset of a spin-crossover equilibrium.

The simulations for $[Fe(bpp^{OH,H})_2][BF_4]_2$ and $[Fe(bpp^{OMe,H})_2][PF_6]_2$ are of lower accuracy than the others, for similar reasons, and the errors on the quoted $T_{½}$ values for those compounds are correspondingly larger. The derived $T_{½}$ values for those compounds are in good agreement with predictions based on the σ_P and σ_P^+ Hammett parameters, however (Fig. 3, main article).

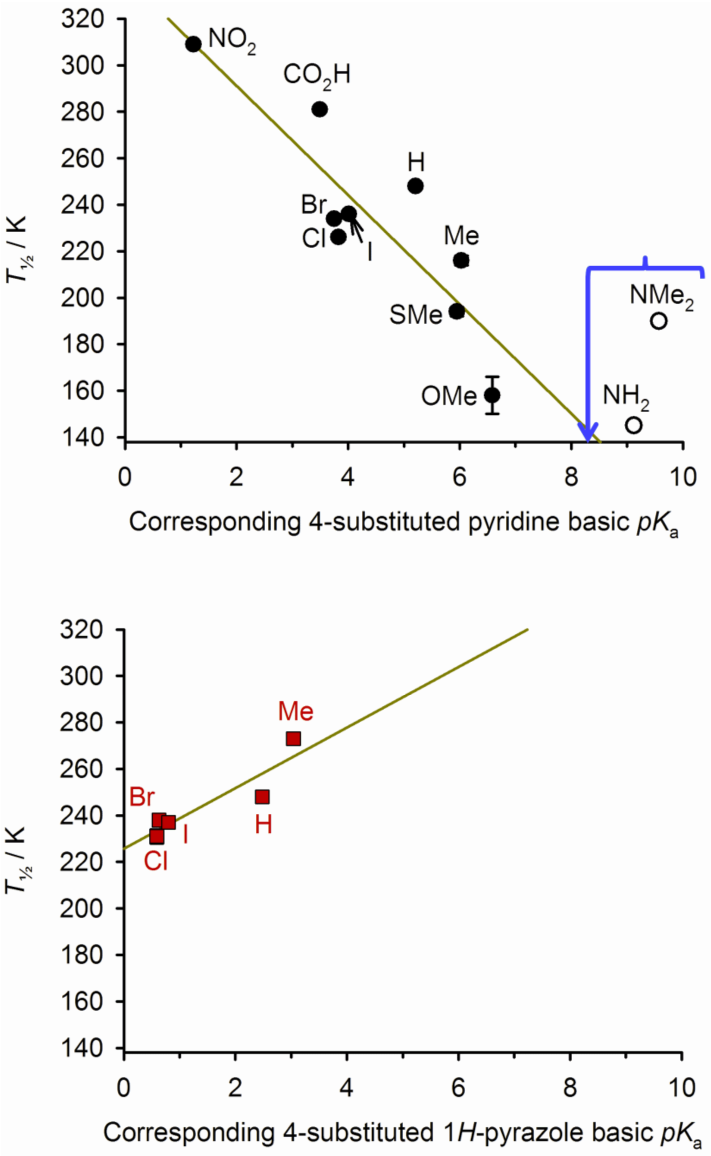

Fig. S2. Plots of the measured $T_{½}$ values for $[Fe(bpp^{X,H})_2]^{2+}$ *vs.* the basic pK_a of the corresponding 4–substituted pyridine[20] (top), and of $[Fe(bpp^{H,Y})_2]^{2+}$ *vs.* the basic pK_a of the corresponding 4-substituted pyrazole[21] (bottom). The lines show the best fit correlations (R^2 = 0.78 [top] and 0.83 [bottom]), omitting the X = NH_2 and NMe_2 datapoints (white circles) whose $T_{½}$ values represent the upper limits for those measurements.

Both series of compounds show reasonable, but opposite, linear relationships between $T_{½}$ and the basicity of the corresponding substituted heterocyclic ligand donor. For $[Fe(bpp^{X,H})_2]^{2+}$, more basic pyridyl donors stabilize the high-spin state of the complex. In contrast, for $[Fe(bpp^{H,Y})_2]^{2+}$, more basic pyrazolyl donor groups favor the low-spin state.

Hence, the different relationships between $T_{½}$ and substituent Hammett parameter in $[Fe(bpp^{X,H})_2]^{2+}$ and $[Fe(bpp^{H,Y})_2]^{2+}$ complexes does not simply reflect the basicity of the $bpp^{X,Y}$ ligands, but must be a function of the metal-ligand interaction.

[20] M. Sawada, M. Ichihara, Y. Yukawa, T. Nakachi, Y. Tsuno, *Bull. Chem. Soc. Jpn.* **1980**, *53*, 2055.

[21] J. Catalan, J. L. M. Abboud, J. Elguero, *Adv. Heterocycl. Chem.* **1987**, *41*, 187.

Table S3 Computed energy difference between the high-spin and low-spin forms of complexes in this work [ΔE_{rel}(HS-LS)]. Experimentally determined $T_{½}$ values and substituent Hammett parameters from the main article are also listed here, for comparison. These data are plotted in Fig. 5 of the main article, and Fig. S4.

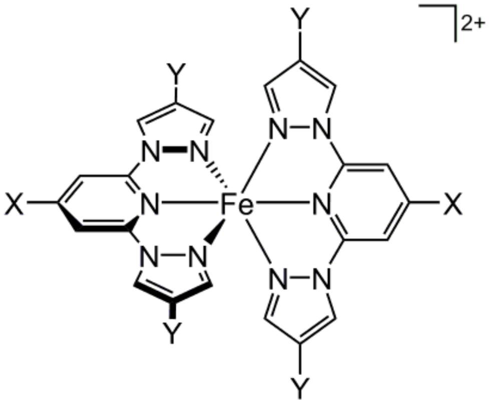

X	Y	σ_P (X)	σ_P^+ (X)	$T_{½}$ (K)	ΔE_{rel}(HS-LS) (kcal.mol^{-1})
H	H	0	0	248(1)	0
Me	H	−0.17	−0.31	216(2)	–0.58
CH_2SCN	H	0.14	–	213(1)	–0.63
CO_2H	H	0.45	0.42	281(1)	0.31
NH_2	H	−0.66	−1.30	<145[a]	–3.03
NMe_2	H	−0.83	−1.70	<190[b]	–2.88
NO_2	H	0.78	0.79	309(2)	0.49
pz (pyrazol-1-yl)	H	–	–	215(3)	–1.29
OH	H	−0.37	−0.92	164(10)	–2.12
OMe	H	−0.27	−0.78	158(8)	–2.11
F	H	0.06	−0.07	215(4)	–1.15
Cl	H	0.23	0.11	226(1)	–0.70
Br	H	0.23	0.15	234(1)	–0.53
I	H	0.18	0.14	236(1)	–0.40
SH	H	0.15	−0.03	246(2)	–1.21
SMe	H	0	−0.60	194(2)	–1.31
SOMe	H	0.49	–	284(1)	–0.51
SO_2Me	H	0.72	–	294(1)	–0.05
X	Y	σ_M (Y)		$T_{½}$ (K)	ΔE_{rel}(HS-LS)
H	H	0		248(1)	0
H	Me	−0.07		273(1)	–0.23
H	CH_2OH	0		259(2)	–0.12
H	*t*Bu	−0.10		251(1)	–0.01
H	CO_2Et	0.37		246(1)	–0.26
H	Cl	0.37		231(3)	–1.11
H	Br	0.39		238(2)	–1.09
H	I	0.35		237(2)	–1.08

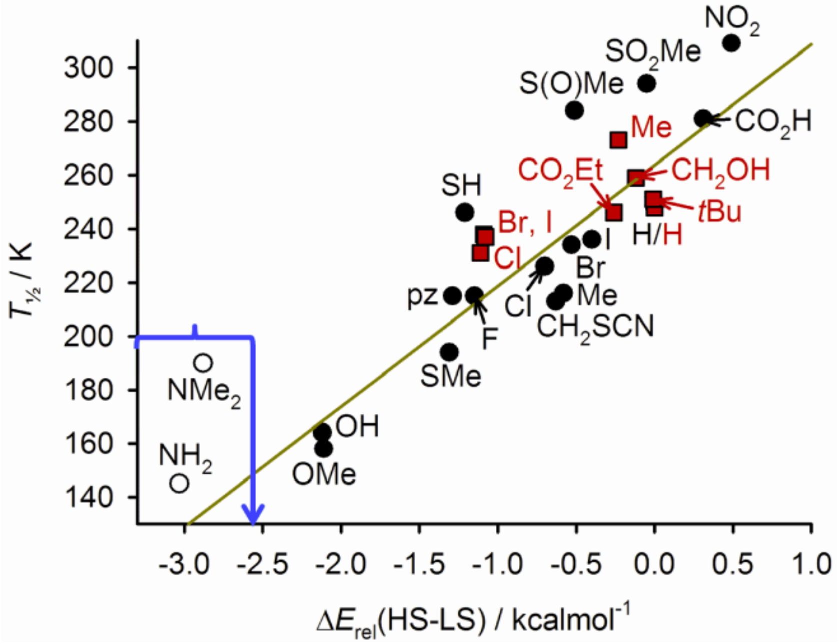

Fig. S3 Plot of the experimentally measured $T_{½}$ *vs* the computed ΔE_{rel}(HS-LS) for $[Fe(bpp^{X,H})_2]^{2+}$ (black circles) and $[Fe(bpp^{H,Y})_2]^{2+}$ (red squares) with different X or Y substituents (Table S3). The line shows the best fit correlation, omitting the X = NH_2 and NMe_2 datapoints (white circles) whose $T_{½}$ values are not properly defined.

The correlation coefficient for this graph is $R^2 = 0.79$.

Table S4 Calculated % composition of the metal *d*-orbitals in low-spin $[Fe(bpp^{H,H})_2]^{2+}$.

orbital #	117	118	119	120	121	122	123	124	125	126	127	128	129
E (Ha)	–0.25573	–0.25550	–0.21594	–0.21594	–0.20231	–0.12822	–0.12821	–0.12057	–0.11976	–0.11122	–0.10106	–0.07535	–0.07534
population	2.00	2.00	2.00	2.00	2.00	0.00	0.00	0.00	0.00	0.00	0.00	0.00	0.00
Fe p_x	0.0	0.0	0.7	0.0	0.0	0.2	0.1	0.0	0.0	0.0	0.0	0.0	0.0
Fe p_y	0.0	0.0	0.0	0.7	0.0	0.1	0.2	0.0	0.0	0.0	0.0	0.0	0.0
Fe p_z	0.0	0.0	0.0	0.0	0.0	0.0	0.0	0.0	0.0	0.2	0.0	0.0	0.0
Fe d_{yz}	0.0	0.0	0.0	0.0	0.0	0.0	0.0	0.0	0.0	0.0	0.0	0.0	0.0
Fe d_{xz}	0.0	0.0	0.1	77.5	0.0	3.6	4.2	0.0	0.0	0.0	0.0	0.0	0.0
Fe d_{yz}	0.0	0.0	77.5	0.1	0.0	4.2	3.6	0.0	0.0	0.0	0.0	0.0	0.0
Fe $d_{x^2-y^2}$	0.3	0.0	0.0	0.0	73.5	0.0	0.0	5.2	0.0	0.0	0.0	0.0	0.0
Fe d_{xy}	0.0	0.0	0.0	0.0	0.0	0.0	0.0	0.0	0.0	69.7	0.0	0.0	0.0
Fe d_{z^2}	0.0	0.0	0.0	0.0	0.0	0.0	0.0	0.0	0.0	0.0	66.5	0.0	0.0

The t_{2g} metal *d* orbitals are the HOMO–2, HOMO–1 and HOMO, while the e_g *d* orbitals are the LUMO+4 and LUMO+5.

The compositions of the metal-based *d*-frontier orbitals show only small differences between the 25 complexes calculated.

Table S5 Computed *d*-orbital energies in the low-spin forms of complexes in this work, and the average energies of the t_{2g} and e_g subshells (all in kcal.mol^{-1}). These data are plotted in Fig. 6 of the main article, and in Fig. S4.

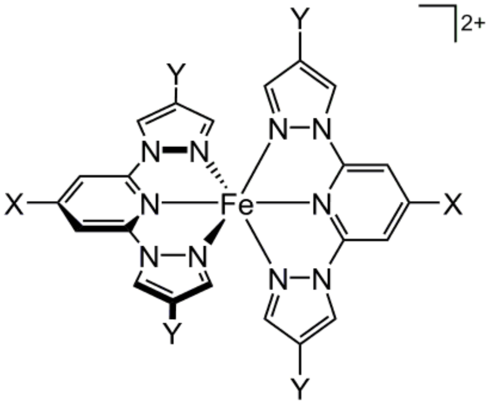

X	Y	$E(e)$ (d_{xz}, d_{yz})	$E(b_1)$ ($d_{x^2-y^2}$)	$E(b_2)$ (d_{xy})	$E(a_1)$ (d_{z^2})	$E_{av}(t_{2g})$	$E_{av}(e_g)$
H	H	–5.8759	–5.5050	–3.0265	–2.7499	–5.7523	–2.8882
Me	H	–5.7556	–5.4269	–2.9517	–2.6505	–5.6461	–2.8011
CH_2SCN	H	–6.0589	–5.6612	–3.2006	–2.9303	–6.0589	–5.6612
CO_2H	H	–6.1386	–5.7001	–3.2404	–2.9672	–5.9924	–3.1038
NH_2	H	–5.2571	–5.1479	–2.6704	–2.3228	–5.2207	–2.4966
NMe_2	H	–5.2165	–5.1367	–2.6606	–2.2996	–5.1899	–2.4801
NO_2	H	–6.3994	–5.9087	–3.4587	–3.2134	–6.2358	–3.3361
pz (pyrazol–1–yl)	H	–5.7880	–5.4931	–3.0314	–2.7255	–5.6897	–2.8785
OH	H	–5.6041	–5.3599	–2.8861	–2.5774	–5.5227	–2.7318
OMe	H	–5.5919	–5.3504	–2.8762	–2.5630	–5.5114	–2.7196
F	H	–5.8966	–5.5580	–3.0837	–2.8117	–5.7837	–2.9477
Cl	H	–5.9478	–5.5931	–3.1207	–2.8561	–5.8296	–2.9884
Br	H	–5.9602	–5.6020	–3.1327	–2.8750	–5.8408	–3.0039
I	H	–5.9532	–5.5984	–3.1311	–2.9118	–5.8349	–3.0215
SH	H	–5.7044	–5.4442	–2.9720	–2.6788	–5.6176	–2.8254
SMe	H	–5.6177	–5.3953	–2.9224	–2.6104	–5.5436	–2.7664
SOMe	H	–5.9810	–5.6214	–3.1514	–2.8802	–5.8611	–3.0158
SO_2Me	H	–6.1561	–5.7363	–3.2729	–3.0172	–6.0162	–3.1451
X	Y	$E(e)$ (d_{xz}, d_{yz})	$E(b_1)$ ($d_{x^2-y^2}$)	$E(b_2)$ (d_{xy})	$E(a_1)$ (d_{z^2})	$E_{av}(t_{2g})$	$E_{av}(e_g)$
H	H	–5.8759	–5.5050	–3.0265	–2.7499	–5.7523	–2.8882
H	Me	–5.7769	–5.3978	–2.9334	–2.6481	–5.6505	–2.7908
H	CH_2OH	–5.8355	–5.4627	–2.9830	–2.6950	–5.7112	–2.8390
H	*t*Bu	–5.7801	–5.3906	–2.9342	–2.6560	–5.6502	–2.7951
H	CO_2Et	–6.1494	–5.8121	–3.3115	–3.0284	–6.0370	–3.1700
H	Cl	–6.1226	–5.7512	–3.3499	–3.0372	–5.9988	–3.1936
H	Br	–6.1287	–5.7581	–3.3771	–3.0664	–6.0051	–3.2218
H	I	–6.1073	–5.7339	–3.3985	–3.0999	–5.9828	–3.2492

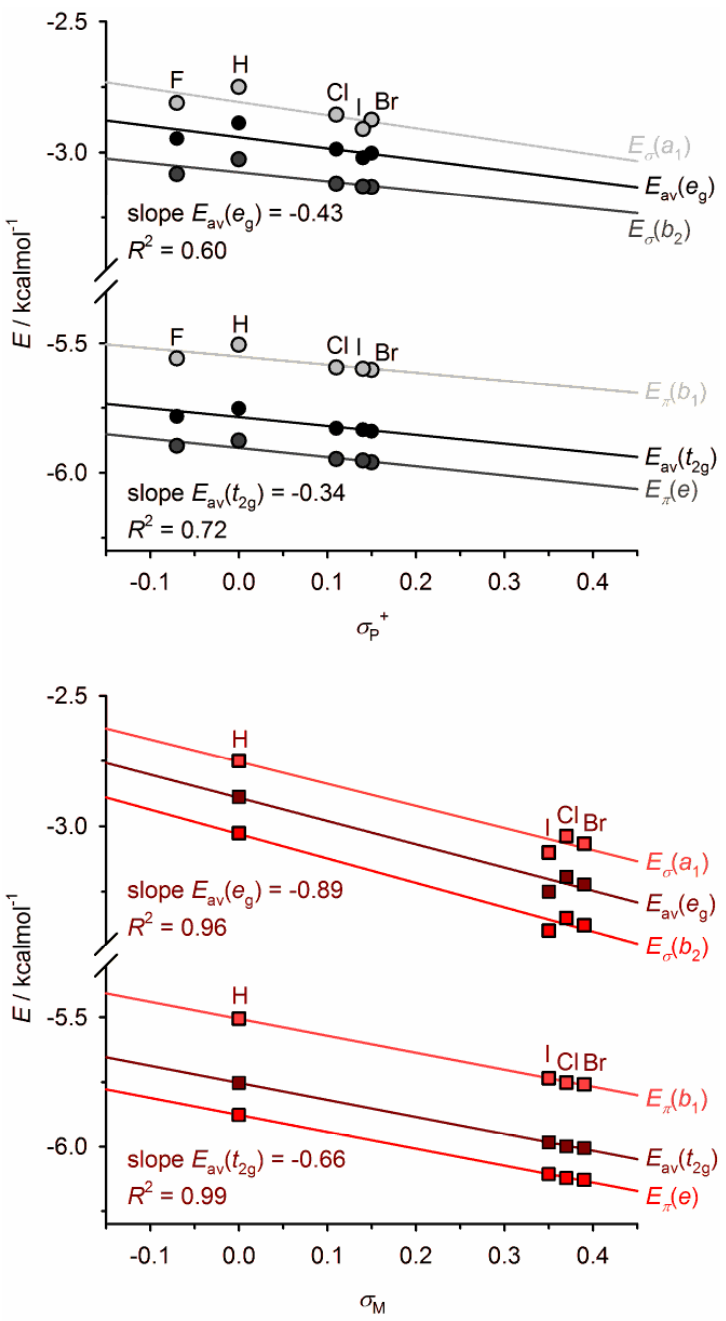

Fig. S4 Plot of the relevant substituent Hammett parameter *vs* the computed *d*-orbital energies for $[Fe(bpp^{X,H})_2]^{2+}$ (top, black/grey circles) and $[Fe(bpp^{H,Y})_2]^{2+}$ (bottom, red squares) with X, Y = H or halogen only (Table S4). The average orbital energies of the t_{2g} and e_g subshells are also shown, along with their best fit correlations and slopes.

The slope of these correlations is almost exactly 2x greater for Y substituents than for X substituents, because there as twice as many Y substituents as X groups in a $[Fe(bpp^{X,Y})_2]^{2+}$ molecule.

The slope of the E_{av} *vs* Hammett parameter correlations is 25 % larger for $E_{av}(e_g)$ than for $E_{av}(t_{2g})$ for both X and Y substituents. This contrasts with the equivalent plots for the complete group of complexes, where X substituents give rise to a steeper correlation with σ_P^+ for $E_{av}(t_{2g})$ than for $E_{av}(e_g)$ (Fig. 6 of the main article).

This explains why the $T_{½}$ for $[Fe(bpp^{X,H})_2]^{2+}$ and $[Fe(bpp^{H,Y})_2]^{2+}$ is affected to a similar extent by halogeno X and Y groups (Fig. 2, main article), but not by other classes of substituent.

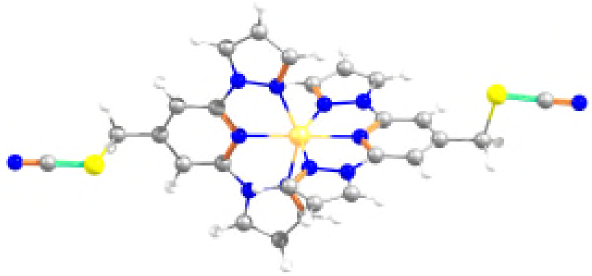

X = CH_2SCN, Y = H

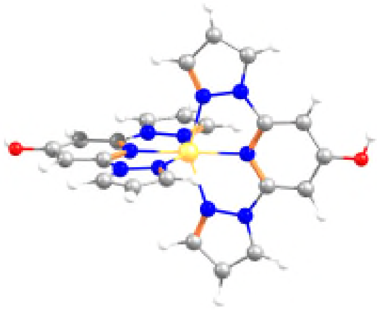

X = OH, Y = H

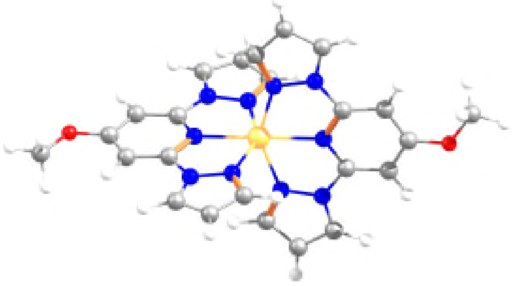

X = OMe, Y = H

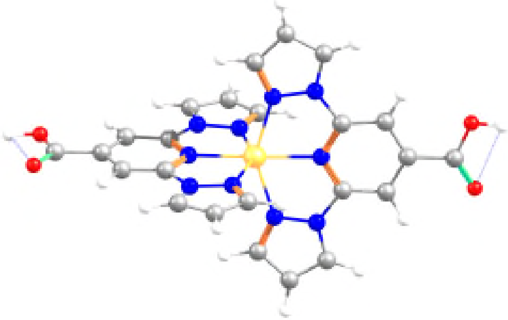

X = CO_2H, Y = H

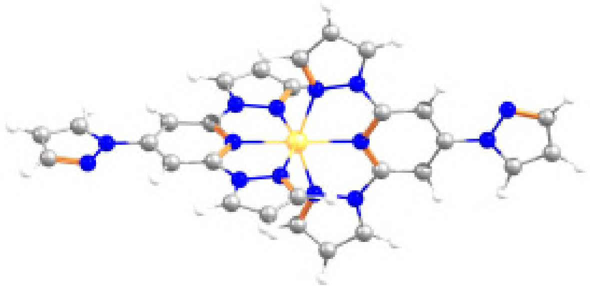

X = pyrazol–1–yl, Y = H

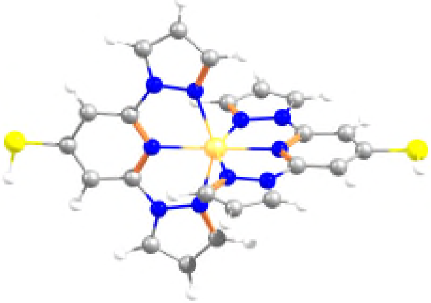

X = SH, Y = H

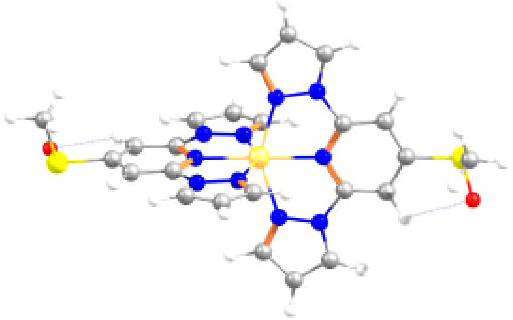

X = S(O)Me, Y = H

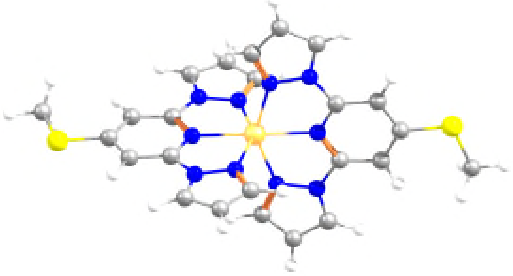

X = SMe, Y = H

Fig. S5 Computed energy-minimized structures of the low-spin forms of a selection of the complexes in this work.

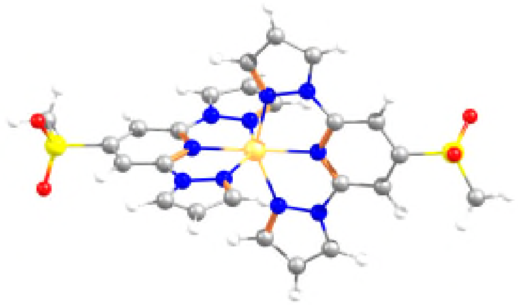

X = SO_2Me, Y = H

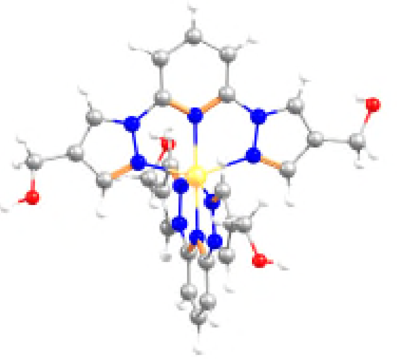

X = H, Y = CH_2OH

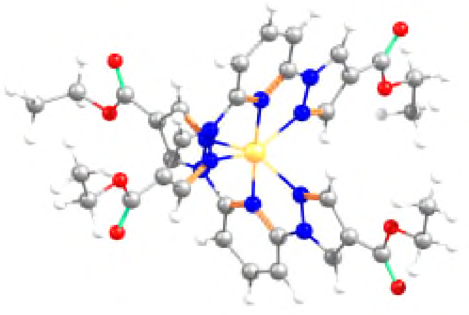

X = H, Y = CO_2Et

Fig. S5 continued

Table S6 Atomic coordinates for the computed, energy-minimized structures of the $[Fe(bpp^{X,H})_2]^{2+}$ and $[Fe(bpp^{H,Y})_2]^{2+}$ complexes in their high-spin and low-spin states.

$[Fe(bpp^{H,H})_2]^{2+}$
51
$FeC_{22}N_{10}H_{18}$

High-spin				Low-spin			
Fe	5.162957284	5.163003480	–0.003561675	Fe	5.162933076	5.162989616	–0.000007095
N	5.163046904	5.163098720	–2.130009192	N	5.163002505	5.163030350	–1.888730253
N	6.651969711	6.652545483	–0.594741586	N	6.526920249	6.526990211	–0.320484675
C	5.987971994	5.988383257	–2.798110563	C	5.994213058	5.994176515	–2.558981127
C	6.029517566	6.030058172	–4.199488558	C	6.031510006	6.031429967	–3.957825535
H	6.703507269	6.704887524	–4.742158155	H	6.707958054	6.707879367	–4.496413192
C	5.163564367	5.162984830	–4.888004256	C	5.163249257	5.163054145	–4.646805723
H	5.163751479	5.162889016	–5.988291374	H	5.163322029	5.163008595	–5.746749683
N	6.787691741	6.788238814	–1.952739592	N	6.756897154	6.756811932	–1.662881212
C	7.732858553	7.733825720	–2.268086707	C	7.719809794	7.719585458	–1.840547813
H	7.979730677	7.980740986	–3.306150186	H	8.036247213	8.035944253	–2.840023182
C	8.227780796	8.225581270	–1.067492214	C	8.121855118	8.121751104	–0.573769960
H	8.997932054	8.993096324	–0.936219408	H	8.877053168	8.876930417	–0.331542181
C	7.520345905	7.521239384	–0.058938552	C	7.352269491	7.352311219	0.339124143
H	7.606820931	7.607229838	1.031700188	H	7.365766787	7.365922787	1.435851373
C	4.297331986	4.295985505	–4.199734185	C	4.294843762	4.294656547	–3.958005421
C	4.338367173	4.337769249	–2.798350267	C	4.331911248	4.331896835	–2.559141757
N	3.538305532	3.537996964	–1.953202773	N	3.569107011	3.569224727	–1.663136115
N	3.673530888	3.673771525	–0.595173405	N	3.798923136	3.798992304	–0.320689195
N	5.163189508	5.163138153	2.129871469	N	5.163009421	5.163042351	1.888711499
C	5.984467304	4.342311859	2.812613516	C	5.994302469	4.332026574	2.559012193
C	6.027726049	4.299577944	4.212916118	C	6.031671833	4.294908141	3.957858714
H	6.704835557	3.622495837	4.749300638	H	6.708173234	3.618541574	4.496483035
C	5.164087485	5.163906077	4.907755487	C	5.163402126	5.163316077	4.646793271
C	4.299991700	6.027830374	4.212988188	C	4.294949939	6.031628122	3.957944487
C	4.342324190	5.984345067	2.812666582	C	4.331954715	5.994251916	2.559078805
N	3.542831722	6.782766943	1.966634893	N	3.569157721	6.756875355	1.663037826
N	3.690860206	6.634043686	0.615916270	N	3.798899880	6.526951403	0.320608472
N	6.634820908	3.691677323	0.615817347	N	6.526983424	3.799026976	0.320536266
N	6.783356671	3.543339039	1.966507727	N	6.757040180	3.569380721	1.662951650
C	7.731089383	2.595587388	2.261063499	C	7.720085096	2.606748322	1.840711145
H	7.988388996	2.338457439	3.293944765	H	8.036598820	2.290565968	2.840218358
C	8.211319523	2.114649804	1.048360739	C	8.121906791	2.204245041	0.573970798
H	8.978350037	1.347050345	0.900382191	H	8.877000036	1.448945548	0.331807682
C	7.496927966	2.829379554	0.054280500	C	7.352424554	2.973712503	–0.338971429
H	7.571762670	2.754523177	–1.037954962	H	7.365956924	2.959916432	–1.435687594
C	2.828097133	7.495734064	0.054470389	C	2.973415791	7.352240973	–0.338875014
H	2.752743794	7.570182687	–1.037756765	H	2.959660018	7.365844683	–1.435591229
C	2.114043931	8.210709274	1.048620029	C	2.204330076	8.122079731	0.574087615
H	1.346669396	8.977979508	0.900682594	H	1.449330952	8.877477491	0.331914839
C	2.594759692	7.730160863	2.261279332	C	2.606273536	7.719664502	1.840818323
H	2.337708118	7.987436827	3.294185622	H	2.289981841	8.036052086	2.840331215
H	3.623268341	6.705216906	4.749483176	H	3.618601426	6.708134212	4.496595625
H	5.164435022	5.164309254	6.007752714	H	5.163517203	5.163465207	5.746736985
C	2.804826725	2.805210079	–0.059689893	C	2.973428918	2.973660644	0.338739004
H	2.717848056	2.719172896	1.030896970	H	2.959725140	2.959933560	1.435455247
C	2.098077665	2.100488267	–1.068436048	C	2.204095765	2.204123685	–0.574271005
H	1.328067922	1.332793604	–0.937396112	H	1.448944135	1.448861382	–0.332162896
C	2.593121451	2.592530025	–2.268861068	C	2.606163963	2.606495245	–1.840978951
H	2.346581165	2.345626956	–3.307006155	H	2.289836497	2.290206285	–2.840511951
H	3.623538906	3.621142721	–4.742637292	H	3.618485461	3.618190943	–4.496694382

$[Fe(bpp^{Me,H})_2]^{2+}$
57
$FeC_{24}N_{10}H_{22}$

High-spin				Low-spin			
Fe	5.163253000	5.160013000	–0.006286000	Fe	5.162954470	5.158992663	–0.000006388
N	5.163414000	5.159810000	–2.124284000	N	5.163389024	5.159230603	–1.887526792
N	6.656703000	6.649000000	–0.600053000	N	6.527398590	6.522665808	–0.323468055
C	5.987154000	5.980717000	–2.800900000	C	5.993243205	5.986684736	–2.562899190
C	6.028815000	6.018425000	–4.197992000	C	6.031066100	6.020828769	–3.958111499
H	6.710727000	6.694084000	–4.731051000	H	6.714800457	6.698659790	–4.487164325
C	5.165026000	5.157251000	–4.916375000	C	5.165947550	5.157350643	–4.676874756
N	6.791207000	6.782557000	–1.958080000	N	6.758067579	6.750558915	–1.666748816
C	7.737188000	7.725623000	–2.275435000	C	7.721210770	7.712127113	–1.845266661
H	7.983908000	7.971174000	–3.313850000	H	8.038315366	8.027247896	–2.844899695
C	8.232787000	8.220450000	–1.075802000	C	8.123840361	8.116228504	–0.578874335
H	9.002814000	8.988469000	–0.946706000	H	8.879544722	8.871265346	–0.337866785
C	7.527070000	7.516857000	–0.065848000	C	7.353468839	7.348690000	0.334755057
H	7.614381000	7.604176000	1.024588000	H	7.366656855	7.363878409	1.431452046
C	4.303966000	4.296562000	–4.198633000	C	4.302800869	4.294437156	–3.959896259
C	4.342106000	4.337943000	–2.799489000	C	4.336482023	4.331181047	–2.562579631
N	3.539189000	3.534217000	–1.958331000	N	3.571646413	3.565830754	–1.668474644
N	3.673056000	3.666728000	–0.600048000	N	3.799185037	3.794867482	–0.324712516
N	5.163336000	5.160979000	2.125709000	N	5.162687445	5.159261225	1.887510620
C	5.980156000	4.343070000	2.814225000	C	5.989752035	4.331304341	2.562487603
C	6.019139000	4.300201000	4.213144000	C	6.023701130	4.294707550	3.959804892
H	6.698075000	3.617099000	4.740835000	H	6.701858439	3.611973397	4.489544068
C	5.160364000	5.158091000	4.935499000	C	5.160692798	5.157691265	4.676864150
C	4.299582000	6.016819000	4.211743000	C	4.295474952	6.021138975	3.958178612
C	4.343352000	5.977623000	2.815030000	C	4.333030158	5.986838207	2.562962918
N	3.540882000	6.778184000	1.970689000	N	3.568119994	6.750721041	1.666875234
N	3.689038000	6.631987000	0.619790000	N	3.798533393	6.522703221	0.323570868
N	6.635755000	3.686718000	0.620708000	N	6.526703918	3.794850683	0.324586093
N	6.781916000	3.540759000	1.972044000	N	6.754472911	3.565924894	1.668324633
C	7.728885000	2.593725000	2.269863000	C	7.716550673	2.603343615	1.848323863
H	7.984775000	2.338214000	3.303483000	H	8.031284290	2.287422914	2.848444959
C	8.211709000	2.110486000	1.058800000	C	8.121330012	2.199723155	0.582532934
H	8.979231000	1.342814000	0.913703000	H	8.876904708	1.444224087	0.342550382
C	7.498818000	2.823338000	0.062310000	C	7.353279891	2.968301074	–0.332294515
H	7.575510000	2.746646000	–1.029658000	H	7.368766114	2.953525670	–1.428959498
C	2.825495000	7.493881000	0.059681000	C	2.972446623	7.348810008	–0.334533723
H	2.750432000	7.570054000	–1.032440000	H	2.959033094	7.364012351	–1.431219248
C	2.110239000	8.205966000	1.054805000	C	2.201940210	8.116117204	0.579162279
H	1.341810000	8.972493000	0.908504000	H	1.445971856	8.870911198	0.338217566
C	2.592420000	7.723867000	2.266738000	C	2.605137788	7.712435813	1.845509307
H	2.334930000	7.979727000	3.299892000	H	2.288268644	8.027676256	2.845179602
H	3.615361000	6.695219000	4.738872000	H	3.611875543	6.699058381	4.487292261
C	2.804011000	2.796943000	–0.067155000	C	2.972494192	2.968367251	0.332091219
H	2.716326000	2.708318000	1.023150000	H	2.956809107	2.953688306	1.428755315
C	2.099485000	2.093250000	–1.078125000	C	2.204677889	2.199642089	–0.582807376
H	1.330508000	1.324042000	–0.949851000	H	1.449093567	1.444132057	–0.342889291
C	2.594610000	2.589832000	–2.277028000	C	2.609601258	2.603226646	–1.848565262
H	2.348657000	2.344984000	–3.315780000	H	2.295020277	2.287243218	–2.848714971
H	3.627408000	3.615686000	–4.731685000	H	3.624748945	3.611646337	–4.489696692
C	5.167950000	5.177352000	6.439429000	C	5.169091353	5.178299462	6.181237846
H	4.136354000	5.184281000	6.844053000	H	4.137818340	5.189433642	6.586115108
H	5.712503000	4.310686000	6.858386000	H	5.710110596	4.309866378	6.600617334
H	5.663195000	6.101973000	6.806795000	H	5.668354940	6.101091086	6.547392597
C	5.156818000	5.176941000	–6.418929000	C	5.157817486	5.177839413	–6.181252197
H	6.188511000	5.189170000	–6.822835000	H	6.189162202	5.188958004	–6.585946697
H	4.612715000	4.311092000	–6.839456000	H	4.616882101	4.309369078	–6.600663212
H	4.660075000	6.102956000	–6.780917000	H	4.658605641	6.100596761	–6.547563539

$[Fe(bpp^{CH2SCN,H})_2]^{2+}$
61
$FeC_{26}N_{12}H_{20}S_2$

High-spin				Low-spin			
Fe	5.161258000	5.186635000	−0.010566000	Fe	5.179371854	5.238754459	−0.017630845
N	6.229992000	4.569910000	−1.745734000	N	6.116686129	4.663923938	−1.548246012
N	6.866979000	6.528870000	−0.187941000	N	6.666152277	6.511723987	0.016872477
C	7.347455000	5.218856000	−2.121202000	C	7.255496018	5.294990508	−1.916376282
C	8.079191000	4.866169000	−3.261484000	C	7.980710944	4.912189802	−3.048307320
H	8.985256000	5.412393000	−3.554575000	H	8.901055685	5.437217828	−3.335991646
C	7.606478000	3.784280000	−4.035114000	C	7.482641126	3.834484743	−3.816902321
N	7.687071000	6.278604000	−1.255475000	N	7.553490930	6.333453723	−1.026120350
C	8.756853000	7.139330000	−1.274174000	C	8.575226407	7.249436035	−0.965905511
H	9.522589000	7.093231000	−2.056337000	H	9.378004640	7.262300433	−1.711924782
C	8.621134000	7.975333000	−0.173210000	C	8.338070032	8.040474596	0.149728807
H	9.293426000	8.786367000	0.125911000	H	8.949575139	8.875409562	0.506947240
C	7.427754000	7.554867000	0.468412000	C	7.139496232	7.543638711	0.728629139
H	6.954145000	7.955992000	1.372398000	H	6.609110383	7.889433613	1.623286182
C	6.440225000	3.095025000	−3.639864000	C	6.296052636	3.171534992	−3.426459714
C	5.785369000	3.536341000	−2.483105000	C	5.642117328	3.628856400	−2.278274759
N	4.605616000	2.959994000	−1.969124000	N	4.457128917	3.133709483	−1.720769288
N	4.080174000	3.483251000	−0.817840000	N	4.013430124	3.769911872	−0.577329304
N	4.106145000	5.853201000	1.713523000	N	4.237859752	5.811578595	1.511669983
C	4.378568000	5.323919000	2.919868000	C	4.525574952	5.273328930	2.719468345
C	3.729669000	5.738727000	4.090438000	C	3.859339773	5.669120295	3.883535396
H	3.965342000	5.294803000	5.066046000	H	4.105098046	5.225421922	4.857029122
C	2.766893000	6.764085000	3.977280000	C	2.866152159	6.668694520	3.766784721
C	2.475203000	7.317850000	2.711701000	C	2.561760823	7.224437890	2.502001189
C	3.177505000	6.821392000	1.607061000	C	3.281354081	6.762085946	1.396317323
N	3.000936000	7.275447000	0.284185000	N	3.153090952	7.173645478	0.064226271
N	3.782682000	6.727426000	−0.698100000	N	3.995915106	6.561032078	−0.843719040
N	5.922737000	4.012578000	1.659298000	N	6.039563087	4.110980770	1.331914342
N	5.373868000	4.326330000	2.874771000	N	5.541264711	4.316983914	2.604003972
C	5.938357000	3.583951000	3.883032000	C	6.169419402	3.505361997	3.516846501
H	5.625311000	3.693706000	4.927004000	H	5.904081198	3.524787536	4.579662123
C	6.889517000	2.761077000	3.293604000	C	7.100156927	2.755242985	2.811508330
H	7.533234000	2.032165000	3.797088000	H	7.781907444	2.000989724	3.218003248
C	6.838458000	3.065671000	1.908449000	C	6.982225667	3.166620171	1.456662190
H	7.423074000	2.635609000	1.085864000	H	7.539122785	2.815759336	0.580016428
C	3.391714000	7.310421000	−1.840219000	C	3.699596580	7.095239858	−2.036562050
H	3.875313000	7.045116000	−2.788220000	H	4.235059003	6.774853638	−2.937882623
C	2.348039000	8.242126000	−1.603125000	C	2.661965249	8.057217039	−1.908274231
H	1.828263000	8.867232000	−2.336676000	H	2.210251133	8.654271120	−2.706997740
C	2.123554000	8.196958000	−0.233119000	C	2.335225379	8.083822761	−0.559532123
H	1.414720000	8.740175000	0.401554000	H	1.595263370	8.667847819	−0.001119449
H	1.729507000	8.116903000	2.605249000	H	1.791656847	7.999825308	2.393144889
C	2.970621000	2.772700000	−0.568498000	C	2.884546598	3.140905011	−0.222499745
H	2.361317000	2.994530000	0.316445000	H	2.325307564	3.456615627	0.666407806
C	2.769755000	1.781532000	−1.563861000	C	2.589920937	2.092518945	−1.135210452
H	1.954502000	1.052971000	−1.629183000	H	1.734950827	1.408927099	−1.104644060
C	3.834845000	1.927268000	−2.443696000	C	3.609636039	2.113257441	−2.076954125
H	4.095516000	1.382542000	−3.357277000	H	3.798137554	1.493446331	−2.960647630
H	6.059025000	2.249832000	−4.227576000	H	5.895330794	2.332024654	−4.009689957
C	2.035886000	7.252354000	5.199055000	C	2.109342295	7.122013164	4.985920348
H	2.602179000	7.049747000	6.126253000	H	2.691982341	6.969291380	5.912768734
H	1.790096000	8.327852000	5.124638000	H	1.790708187	8.177272127	4.901882252
C	8.360618000	3.350811000	−5.263254000	C	8.233181041	3.375478579	−5.037416089
H	7.703803000	2.842627000	−5.992355000	H	7.568250343	2.880309480	−5.768009703
H	8.882280000	4.199345000	−5.742512000	H	8.781453014	4.206221869	−5.518089443
S	0.411781000	6.308947000	5.300677000	S	0.555067843	6.068252881	5.107552706
S	9.669157000	2.117098000	−4.713917000	S	9.505259267	2.108250848	−4.473609514
C	−0.275826000	7.061373000	6.654457000	C	−0.159115746	6.752271154	6.482912836
N	−0.803294000	7.551327000	7.588738000	N	−0.700250917	7.191956932	7.434329470
C	10.439489000	1.825676000	−6.194311000	C	10.245856584	1.754813544	−5.955567482
N	11.010192000	1.585242000	−7.198227000	N	10.795230208	1.471668618	−6.960240781

$[Fe(bpp^{CO2H,H})_2]^{2+}$
57
$FeC_{24}N_{10}H_{18}O_4$

High-spin				Low-spin			
Fe	5.162220000	5.176500000	0.000466000	Fe	5.133941977	5.162716886	–0.000013654
N	5.189155000	5.151350000	–2.123825000	N	5.134926341	5.181464807	–1.882303553
N	6.607264000	6.688631000	–0.602127000	N	6.498712264	6.531448905	–0.304031751
C	6.010103000	5.978169000	–2.799323000	C	5.966666185	6.019292945	–2.544993805
C	6.075162000	5.998545000	–4.197149000	C	6.006895230	6.071096855	–3.941269537
H	6.743550000	6.670162000	–4.751571000	H	6.682739823	6.751896943	–4.473261363
C	5.239386000	5.105167000	–4.894551000	C	5.138665953	5.207655359	–4.644709480
N	6.780236000	6.804101000	–1.956086000	N	6.728655441	6.774180627	–1.644343312
C	7.737272000	7.741711000	–2.258155000	C	7.691615744	7.738975323	–1.812578385
H	8.011159000	7.974510000	–3.292656000	H	8.008279439	8.065415377	–2.808799806
C	8.200098000	8.247939000	–1.050479000	C	8.092980663	8.128343382	–0.542170979
H	8.966955000	9.016702000	–0.907970000	H	8.847877750	8.881141603	–0.291930545
C	7.463609000	7.561230000	–0.051142000	C	7.323251137	7.350033852	0.363634537
H	7.516306000	7.667687000	1.039766000	H	7.337503408	7.354138275	1.460462101
C	4.375828000	4.235243000	–4.199635000	C	4.269488679	4.332372218	–3.958855626
C	4.391908000	4.303885000	–2.800423000	C	4.304332110	4.355696828	–2.563165187
N	3.590415000	3.506077000	–1.959241000	N	3.540781043	3.583792279	–1.678379118
N	3.719000000	3.661381000	–0.604586000	N	3.768737789	3.800444311	–0.333197771
N	5.136418000	5.151380000	2.126559000	N	5.134697917	5.144156435	1.882269888
C	5.934291000	4.304208000	2.802535000	C	5.966417334	4.306480603	2.545168438
C	5.951826000	4.236217000	4.201776000	C	6.006588873	4.254938614	3.941457245
H	6.603477000	3.539457000	4.742452000	H	6.682379858	3.574214983	4.473613466
C	5.089599000	5.107164000	4.897110000	C	5.138329666	5.118524943	4.644691860
C	4.253557000	6.000728000	4.200179000	C	4.269279935	5.993768661	3.958625994
C	4.316713000	5.979052000	2.802277000	C	4.304170179	5.970159505	2.562936596
N	3.545832000	6.804260000	1.958974000	N	3.540815033	6.742040273	1.677978469
N	3.718358000	6.688390000	0.605028000	N	3.768790492	6.525078064	0.332854426
N	6.604774000	3.661319000	0.606093000	N	6.498671233	3.793947191	0.304318157
N	6.734850000	3.506302000	1.960592000	N	6.728533678	3.551486603	1.644696060
C	7.689501000	2.567052000	2.265435000	C	7.691578010	2.586817863	1.813215760
H	7.932716000	2.305252000	3.300646000	H	8.008148331	2.260567636	2.809529322
C	8.195328000	2.100903000	1.058938000	C	8.093520777	2.197665880	0.542924618
H	8.973308000	1.343044000	0.918448000	H	8.848842429	1.445225768	0.292901609
C	7.485490000	2.811893000	0.057372000	C	7.323354282	2.975285505	–0.363086006
H	7.574609000	2.738239000	–1.033913000	H	7.337746559	2.970864151	–1.459902030
C	2.861200000	7.560343000	0.054090000	C	2.943579933	7.356694823	–0.317655128
H	2.807943000	7.666549000	–1.036813000	H	2.927621389	7.381789091	–1.414151850
C	2.124488000	8.246659000	1.053431000	C	2.174877890	8.117042307	0.604085906
H	1.356905000	9.014685000	0.910915000	H	1.419401123	8.874271558	0.369486597
C	2.588285000	7.741212000	2.261115000	C	2.578454980	7.703670053	1.866221061
H	2.314584000	7.974005000	3.295669000	H	2.262915705	8.009916059	2.869237591
H	3.586739000	6.673423000	4.755198000	H	3.600353915	6.658827710	4.520752135
C	2.837590000	2.812291000	–0.056630000	C	2.943222423	2.968962562	0.317104814
H	2.747307000	2.738925000	1.034576000	H	2.927170890	2.943692240	1.413597148
C	2.128802000	2.101038000	–1.058837000	C	2.174536046	2.208824025	–0.604821800
H	1.350573000	1.343292000	–0.919059000	H	1.418876073	1.451717335	–0.370397176
C	2.635868000	2.566892000	–2.264882000	C	2.578109691	2.622509888	–1.866857879
H	2.393719000	2.304785000	–3.300266000	H	2.262516175	2.316570597	–2.869951631
H	3.723977000	3.538654000	–4.740286000	H	3.600548707	3.667464869	–4.521142059
C	5.023964000	5.118384000	6.404231000	C	5.102600170	5.142304537	6.150461025
O	4.292340000	5.865758000	7.034275000	O	4.365248534	5.876994257	6.789528683
O	5.854026000	4.218928000	6.952455000	O	5.970150783	4.272768790	6.690918238
H	5.765366000	4.271006000	7.932099000	H	5.898841195	4.334162010	7.671383762
C	5.307171000	5.115016000	–6.401725000	C	5.103200860	5.183990620	–6.150486036
O	6.042006000	5.859874000	–7.030938000	O	4.363912980	4.451400148	–6.789729614
O	4.474852000	4.218035000	–6.950415000	O	5.968600929	6.055814396	–6.690708230
H	4.565862000	4.268566000	–7.929950000	H	5.895728014	5.996226600	–7.671170034

$[Fe(bpp^{NH2,H})_2]^{2+}$
55
$FeC_{22}N_{12}H_{20}$

High-spin				Low-spin			
Fe	5.162864000	5.158786000	–0.001125000	Fe	5.163011882	5.160779667	–0.000005638
N	5.137780000	5.161793000	–2.114383000	N	5.139406616	5.161292723	–1.893257822
N	6.673405000	6.617008000	–0.629454000	N	6.522766672	6.524269654	–0.349917118
C	5.948456000	5.980178000	–2.818014000	C	5.956236241	5.988826204	–2.585851099
C	5.983077000	6.030193000	–4.206243000	C	5.981122515	6.038314110	–3.973905867
H	6.664224000	6.703172000	–4.743091000	H	6.653229942	6.720474829	–4.511230608
C	5.098926000	5.170915000	–4.931704000	C	5.099829892	5.166733459	–4.693166130
N	6.768446000	6.781913000	–1.983801000	N	6.734674380	6.753073655	–1.696240343
C	7.675892000	7.759155000	–2.303619000	C	7.694903894	7.713966664	–1.887956739
H	7.888438000	8.033610000	–3.342192000	H	7.998003464	8.029313846	–2.891791979
C	8.185899000	8.246257000	–1.105181000	C	8.116887700	8.118055411	–0.627047210
H	8.935295000	9.034992000	–0.979026000	H	8.876739066	8.872599366	–0.397560481
C	7.528881000	7.500322000	–0.093872000	C	7.359051589	7.350420538	0.296532472
H	7.643535000	7.562118000	0.995658000	H	7.386526123	7.363603240	1.392918838
C	4.234848000	4.307022000	–4.187561000	C	4.238714222	4.292790828	–3.952513201
C	4.307847000	4.348113000	–2.800512000	C	4.302966518	4.336929971	–2.565555825
N	3.510622000	3.541290000	–1.949145000	N	3.548191302	3.570821305	–1.657236758
N	3.638658000	3.701927000	–0.596919000	N	3.795181036	3.797691388	–0.316499585
N	5.187937000	5.161320000	2.116216000	N	5.186715289	5.161324682	1.893243867
C	6.017439000	4.347900000	2.802792000	C	6.023270885	4.337050641	2.565502884
C	6.090623000	4.306425000	4.189968000	C	6.087668120	4.293001211	3.952458724
H	6.787412000	3.637123000	4.711141000	H	6.775790162	3.613697360	4.472952866
C	5.226845000	5.170000000	4.934529000	C	5.226600333	5.166954811	4.693147721
C	4.343224000	6.029555000	4.208920000	C	4.345244241	6.038504353	3.973923298
C	4.377847000	5.979416000	2.820556000	C	4.369993544	5.988924989	2.585873454
N	3.558092000	6.781295000	1.986270000	N	3.591502255	6.753168528	1.696290154
N	3.654533000	6.616036000	0.632415000	N	3.803264514	6.524276070	0.349960589
N	6.685510000	3.702971000	0.599536000	N	6.530861184	3.797710878	0.316435410
N	6.814652000	3.541363000	1.951193000	N	6.778002231	3.570938536	1.657157760
C	7.727867000	2.563014000	2.250254000	C	7.742617681	2.610062320	1.825186195
H	7.965755000	2.290614000	3.283811000	H	8.071721390	2.296406768	2.821321391
C	8.205900000	2.071601000	1.040316000	C	8.130817233	2.203341317	0.554294261
H	8.949719000	1.280505000	0.897101000	H	8.883577811	1.447624415	0.306045701
C	7.525127000	2.816443000	0.044127000	C	7.349543993	2.970056381	–0.350338914
H	7.611768000	2.751312000	–1.047836000	H	7.348296480	2.954951478	–1.447058026
C	2.799207000	7.498706000	0.095168000	C	2.966921633	7.350421862	–0.296422679
H	2.685612000	7.560191000	–0.994481000	H	2.939271268	7.363580356	–1.392795748
C	2.140860000	8.244582000	1.105533000	C	2.209254316	8.118180622	0.627178111
H	1.391198000	9.032893000	0.978314000	H	1.449432365	8.872761883	0.397711910
C	2.649908000	7.758187000	2.304768000	C	2.631320156	7.714101391	1.888064716
H	2.436231000	8.032818000	3.343044000	H	2.328320220	8.029491786	2.891916568
H	3.662052000	6.702930000	4.745289000	H	3.673200703	6.720705586	4.511275783
C	2.799025000	2.814410000	–0.043447000	C	2.976438626	2.970063201	0.350237750
H	2.711493000	2.748343000	1.048392000	H	2.977552106	2.955046555	1.446958726
C	2.119179000	2.070160000	–1.040798000	C	2.195337325	2.203212075	–0.554429661
H	1.375480000	1.278721000	–0.898858000	H	1.442537462	1.447523862	–0.306199059
C	2.598147000	2.562699000	–2.249816000	C	2.583610589	2.609909493	–1.825307346
H	2.361306000	2.290993000	–3.283793000	H	2.254597739	2.296205496	–2.821457715
H	3.538304000	3.638092000	–4.709519000	H	3.550671519	3.613431751	–4.473038960
N	5.244906000	5.172550000	6.282366000	N	5.246251900	5.168889544	6.043140309
H	4.640994000	5.795804000	6.819035000	H	4.639484790	5.788419806	6.580422776
H	5.873930000	4.562076000	6.804678000	H	5.869437795	4.551969251	6.564380270
N	5.078930000	5.175610000	–6.279270000	N	5.080293328	5.168600177	–6.043159005
H	5.691701000	5.789936000	–6.816269000	H	5.687095649	5.788113391	–6.580421711
H	4.458619000	4.556689000	–6.802135000	H	4.457153274	4.551652476	–6.564421269

[Fe(bpp^{NMe2,H})$_2$]$^{2+}$
67
$FeC_{26}N_{12}H_{28}$

High-spin				Low-spin			
Fe	5.162635000	5.147823000	0.001612000	Fe	5.162858360	5.155363009	0.000007739
N	5.049838000	5.158682000	–2.107425000	N	5.058809606	5.159344132	–1.888244903
N	6.643391000	6.609648000	–0.685454000	N	6.506840969	6.519037950	–0.405698629
C	5.829791000	5.977470000	–2.843638000	C	5.843802875	5.986956995	–2.615589422
C	5.812266000	6.030381000	–4.231995000	C	5.813349429	6.038630621	–4.003244660
H	6.481378000	6.711296000	–4.767709000	H	6.471888698	6.728730588	–4.541002807
C	4.902980000	5.170539000	–4.935344000	C	4.902551626	5.168449262	–4.697534956
N	6.681671000	6.780401000	–2.041614000	N	6.659709934	6.751431033	–1.759161916
C	7.572900000	7.761135000	–2.393980000	C	7.610772888	7.712725992	–1.989404247
H	7.741322000	8.042419000	–3.438850000	H	7.870016643	8.031681464	–3.004359992
C	8.131291000	8.244238000	–1.215507000	C	8.087406178	8.113275897	–0.746825595
H	8.883473000	9.034308000	–1.117416000	H	8.856658054	8.867183610	–0.548806781
C	7.518494000	7.492486000	–0.181299000	C	7.370539904	7.343104108	0.206598894
H	7.677903000	7.549173000	0.902756000	H	7.445208606	7.352537997	1.300760516
C	4.069853000	4.305713000	–4.148234000	C	4.072831818	4.294789095	–3.911613267
C	4.196806000	4.346580000	–2.765220000	C	4.196680054	4.337453830	–2.528989965
N	3.432991000	3.536401000	–1.885345000	N	3.480051102	3.568382287	–1.591529350
N	3.621371000	3.687763000	–0.539237000	N	3.783237074	3.792246762	–0.261937397
N	5.276144000	5.158838000	2.112532000	N	5.267091397	5.159448260	1.888244063
C	6.129680000	4.347196000	2.770021000	C	6.129423912	4.337708119	2.528896796
C	6.257647000	4.306704000	4.152966000	C	6.253540384	4.295195954	3.911492621
H	6.978784000	3.630313000	4.622868000	H	6.968486933	3.609305199	4.378064001
C	5.424976000	5.171680000	4.940398000	C	5.424014002	5.168958652	4.697495893
C	4.515032000	6.031092000	4.237392000	C	4.513030302	6.039031728	4.003299656
C	4.496626000	5.977638000	2.849015000	C	4.482291558	5.987182528	2.615653620
N	3.644092000	6.779958000	2.047032000	N	3.666296524	6.751633511	1.759263169
N	3.681382000	6.607686000	0.691134000	N	3.818911075	6.519057811	0.405799144
N	6.703386000	3.688540000	0.543879000	N	6.542524808	3.792303554	0.261841698
N	6.892943000	3.536955000	1.889680000	N	6.845955938	3.568604924	1.591398608
C	7.822879000	2.564670000	2.154355000	C	7.815373795	2.607016130	1.720636936
H	8.108269000	2.300553000	3.177911000	H	8.186311931	2.295686429	2.702663893
C	8.249043000	2.066552000	0.927717000	C	8.148783978	2.196166846	0.435427020
H	8.989163000	1.277355000	0.757337000	H	8.889046173	1.438453317	0.157456770
C	7.521700000	2.801829000	–0.042253000	C	7.331011038	2.961782628	–0.437149484
H	7.559470000	2.728824000	–1.136559000	H	7.282677575	2.943960058	–1.532779587
C	2.806101000	7.490135000	0.186526000	C	2.955179131	7.343151508	–0.206416567
H	2.645794000	7.545694000	–0.897449000	H	2.880269618	7.352512938	–1.300552612
C	2.194135000	8.243114000	1.220317000	C	2.238558203	8.113518276	0.747022768
H	1.442041000	9.033219000	1.121848000	H	1.469384711	8.867495974	0.548972314
C	2.753296000	7.761231000	2.398950000	C	2.715367151	7.713051075	1.989563796
H	2.585823000	8.043636000	3.443663000	H	2.456346416	8.032156824	3.004529679
H	3.845979000	6.711942000	4.773313000	H	3.854709425	6.729301397	4.541120479
C	2.802613000	2.800841000	0.045857000	C	2.994816916	2.961612404	0.436995327
H	2.763860000	2.727617000	1.140116000	H	3.042988101	2.943891722	1.532634827
C	2.076159000	2.065682000	–0.924891000	C	2.177342764	2.195745541	–0.435642378
H	1.335993000	1.276369000	–0.755282000	H	1.437263406	1.437847686	–0.157695072
C	2.503368000	2.564053000	–2.151027000	C	2.510875190	2.606548955	–1.720836563
H	2.218828000	2.300079000	–3.174853000	H	2.140160268	2.295053057	–2.702895480
H	3.349148000	3.629111000	–4.618489000	H	3.357994917	3.608836571	–4.378271564
N	5.495228000	5.175662000	6.296966000	N	5.499640425	5.172012638	6.056018792
N	4.833760000	5.174087000	–6.291929000	N	4.827392591	5.171337278	–6.056094659
C	6.423821000	4.282455000	6.989731000	C	6.431851068	4.279847816	6.743524373
H	6.321870000	4.432380000	8.078820000	H	6.336573496	4.430529539	7.833190140
H	6.206974000	3.216178000	6.764639000	H	6.214550655	3.212705867	6.521406917
H	7.477946000	4.493315000	6.708980000	H	7.484765965	4.489626967	6.456500667
C	4.641416000	6.072167000	7.076550000	C	4.650176374	6.068878229	6.838359317
H	3.564041000	5.860273000	6.905098000	H	3.571548128	5.864202052	6.664721095
H	4.853732000	5.927532000	8.150339000	H	4.859783492	5.918002615	7.911957712
H	4.834516000	7.137173000	6.826214000	H	4.848186347	7.134821010	6.594230557
C	5.688283000	6.070277000	–7.071140000	C	5.677125200	6.068161633	–6.838155899
H	6.765425000	5.859847000	–6.896702000	H	6.755676975	5.863478412	–6.664118270
H	5.478734000	5.923297000	–8.145142000	H	5.467925363	5.917236998	–7.911824903
H	5.493382000	7.135493000	–6.823072000	H	5.478979917	7.134115083	–6.594175236
C	3.904718000	4.281640000	–6.985088000	C	3.895090497	4.279468095	–6.743876109
H	4.007454000	4.431246000	–8.074162000	H	3.991088684	4.429729498	–7.833540094

H	4.120459000	3.215211000	–6.759666000	H	4.111712891	3.212270234	–6.521346670
H	2.850632000	4.493491000	–6.704942000	H	2.842088931	4.489798199	–6.457539257

$[Fe(bpp^{NO2,H})_2]^{2+}$
55
$FeC_{22}N_{12}H_{16}O_4$

High-spin				Low-spin			
Fe	5.162993000	5.162895000	0.000864000	Fe	5.162677146	5.162833647	–0.000010138
N	5.163057000	5.163004000	–2.126237000	N	5.162864421	5.162974088	–1.880695475
N	6.603413000	6.673330000	–0.602448000	N	6.528019316	6.528903886	–0.316298508
C	5.973189000	6.003509000	–2.798983000	C	5.996090198	5.997238397	–2.549835670
C	6.017421000	6.051118000	–4.198863000	C	6.036641974	6.037918660	–3.946435275
H	6.670166000	6.728316000	–4.762662000	H	6.701927687	6.704262902	–4.509120250
C	5.163097000	5.163170000	–4.863502000	C	5.163317638	5.163048756	–4.610259775
N	6.755415000	6.813218000	–1.957430000	N	6.757224072	6.759321601	–1.659719966
C	7.708610000	7.756727000	–2.257743000	C	7.720417246	7.723368352	–1.837609388
H	7.966914000	8.008242000	–3.291928000	H	8.036568227	8.040557992	–2.837097628
C	8.189554000	8.240249000	–1.048900000	C	8.121308986	8.123934965	–0.571520161
H	8.959030000	9.005878000	–0.903895000	H	8.875978186	8.879106412	–0.327865703
C	7.468347000	7.535961000	–0.050044000	C	7.352267215	7.353608595	0.342492687
H	7.539675000	7.623666000	1.041518000	H	7.368253382	7.368836259	1.439234828
C	4.308780000	4.275126000	–4.198965000	C	4.289780164	4.288131980	–3.946764038
C	4.352957000	4.322571000	–2.799095000	C	4.329884239	4.328743187	–2.550155217
N	3.570724000	3.512708000	–1.957682000	N	3.568516490	3.566520022	–1.660319743
N	3.722595000	3.652418000	–0.602669000	N	3.797334973	3.796744179	–0.316788435
N	5.163070000	5.163057000	2.127399000	N	5.162822625	5.162900422	1.880664194
C	5.973203000	4.322592000	2.800204000	C	5.996165709	4.328864608	2.549929947
C	6.017634000	4.275383000	4.200114000	C	6.036879469	4.288527695	3.946534600
H	6.670366000	3.598215000	4.763956000	H	6.702252265	3.622353762	4.509315589
C	5.163605000	5.163708000	4.864575000	C	5.163585567	5.163528493	4.610243203
C	4.309361000	6.051752000	4.200011000	C	4.289996917	6.038301677	3.946625658
C	4.353233000	6.003880000	2.800113000	C	4.329945773	5.997339685	2.550012031
N	3.570890000	6.813683000	1.958733000	N	3.568624027	6.759466170	1.660076389
N	3.722186000	6.673704000	0.603644000	N	3.797333253	6.528926516	0.316583432
N	6.603700000	3.652048000	0.603799000	N	6.528078510	3.796787832	0.316457033
N	6.755316000	3.512488000	1.958897000	N	6.757364464	3.566692691	1.659925077
C	7.708368000	2.568958000	2.259787000	C	7.720764305	2.602882739	1.838024440
H	7.966337000	2.317691000	3.294118000	H	8.036992878	2.285978506	2.837579270
C	8.189683000	2.085138000	1.051247000	C	8.121659697	2.202064530	0.572016677
H	8.959204000	1.319491000	0.906593000	H	8.876477004	1.447002575	0.328493223
C	7.468722000	2.789176000	0.052003000	C	7.352492294	2.972081036	–0.342136960
H	7.540523000	2.701071000	–1.039490000	H	7.368562729	2.956543988	–1.438866203
C	2.857160000	7.536536000	0.051788000	C	2.973047182	7.353829558	–0.341926795
H	2.785129000	7.624331000	–1.039716000	H	2.956597918	7.369086775	–1.438654394
C	2.136514000	8.240965000	1.050984000	C	2.204586465	8.124464220	0.572301534
H	1.367172000	9.006783000	0.906315000	H	1.450175221	8.879964634	0.328896771
C	2.618048000	7.757445000	2.259558000	C	2.605749113	7.723784476	1.838268009
H	2.360356000	8.009077000	3.293869000	H	2.290017791	8.041087750	2.837853413
H	3.656932000	6.729242000	4.763816000	H	3.625019158	6.704813641	4.509475454
C	2.857653000	2.789633000	–0.050519000	C	2.972922626	2.971853371	0.341589070
H	2.786076000	2.701653000	1.041000000	H	2.956518510	2.956380222	1.438315478
C	2.136577000	2.085449000	–1.049526000	C	2.204229609	2.201591050	–0.572757892
H	1.367144000	1.319749000	–0.904695000	H	1.449558656	1.446313333	–0.329445877
C	2.617648000	2.569164000	–2.258239000	C	2.605425642	2.602434335	–1.838665174
H	2.359489000	2.317757000	–3.292488000	H	2.289582299	2.285406290	–2.838303247
H	3.656113000	3.597966000	–4.762893000	H	3.624728834	3.621778532	–4.509711257
N	5.163904000	5.164045000	6.356566000	N	5.164103677	5.163933521	6.098857427
O	5.914884000	4.367664000	6.917403000	O	5.936160420	4.387986988	6.660766529
O	4.413227000	5.960726000	6.917388000	O	4.392589987	5.940308146	6.660926039
N	5.163093000	5.163239000	–6.355391000	N	5.163661169	5.162955406	–6.098877290
O	4.412326000	4.366643000	–6.916292000	O	4.391931974	4.386829448	–6.660984609
O	5.913784000	5.959920000	–6.916273000	O	5.935488056	5.939161330	–6.660757658

$[Fe(bpp^{pz,H})_2]^{2+}$ (pz = 1–pyrazolyl)
65
$FeC_{28}N_{14}H_{22}$

High-spin				Low-spin			
Fe	5.184478421	5.163017918	–0.000020118	Fe	5.184346943	5.162909197	–0.000005961
N	5.183492378	5.126663981	–2.120199158	N	5.183926182	5.130827108	–1.885971073
N	6.696383844	6.606227524	–0.635445020	N	6.549360047	6.522124750	–0.350220905
C	6.010855527	5.928409965	–2.819111233	C	6.010888602	5.949211311	–2.577542247
C	6.058610862	5.952018258	–4.212785537	C	6.051398561	5.969917876	–3.969634940
H	6.734644448	6.602743581	–4.778506143	H	6.718916011	6.629795597	–4.535561122
C	5.180811777	5.084296082	–4.904551851	C	5.180068609	5.088412221	–4.657243145
N	6.821499925	6.736400036	–1.992787431	N	6.776158948	6.728379379	–1.698063928
C	7.748665656	7.694997787	–2.318443479	C	7.738045916	7.687958753	–1.895627233
H	7.986054983	7.938849284	–3.359326229	H	8.051527222	7.985973669	–2.901672498
C	8.238669800	8.204439218	–1.122508087	C	8.143288527	8.112921146	–0.637469004
H	8.995453270	8.986460172	–0.999472871	H	8.898832191	8.872567826	–0.410921970
C	7.552550365	7.491821259	–0.105670564	C	7.376274343	7.359584119	0.291347034
H	7.647858770	7.581121809	0.983833176	H	7.393123139	7.392988556	1.387569599
C	4.304174999	4.237979097	–4.185730490	C	4.310062619	4.228804773	–3.941702074
C	4.355360708	4.304724333	–2.791566389	C	4.355604777	4.292810101	–2.548967967
N	3.545898313	3.522482510	–1.938993390	N	3.591664614	3.541413378	–1.644160340
N	3.670900446	3.700290001	–0.586878793	N	3.820433191	3.792569249	–0.303994385
N	5.183573993	5.199491730	2.120177564	N	5.183946490	5.195132928	1.885953559
C	6.010989588	4.397836143	2.819135492	C	6.011010762	4.376919327	2.577594400
C	6.058816947	4.374363257	4.212811108	C	6.051617397	4.356409220	3.969688998
H	6.734880694	3.723691956	4.778557846	H	6.719186118	3.696629141	4.535668457
C	5.181025468	5.242126452	4.904538072	C	5.180288977	5.237977929	4.657226330
C	4.304358850	6.088370272	4.185671683	C	4.310277364	6.097518817	3.941611705
C	4.355473189	6.021490145	2.791511796	C	4.355719484	6.033302876	2.548880780
N	3.545940302	6.803625595	1.938912507	N	3.591813883	6.784655213	1.644022041
N	3.670862230	6.625674666	0.586811905	N	3.820458582	6.533265479	0.303882996
N	6.696439587	3.719825541	0.635499500	N	6.549431655	3.803742271	0.350311498
N	6.821608873	3.589773847	1.992847583	N	6.776346263	3.597731361	1.698175150
C	7.748818272	2.631238921	2.318562895	C	7.738432235	2.638365263	1.895848635
H	7.986248128	2.387494357	3.359461490	H	8.052017938	2.340573466	2.901927857
C	8.238814518	2.121709933	1.122661797	C	8.143686028	2.213264948	0.637739602
H	8.995595201	1.339684763	0.999629984	H	8.899353488	1.453726822	0.411230948
C	7.552624325	2.834208461	0.105794959	C	7.376478922	2.966316699	–0.291136672
H	7.647921133	2.744732552	–0.983691901	H	7.393340459	2.932680734	–1.387343774
C	2.817598898	7.494435341	0.025902346	C	2.995620979	7.349787995	–0.366572463
H	2.722627222	7.545829773	–1.066136626	H	2.980694581	7.346932586	–1.463355464
C	2.133093678	8.244478122	1.016588705	C	2.227917020	8.134653508	0.535070354
H	1.378762620	9.024034046	0.865913928	H	1.473743078	8.887243789	0.281949177
C	2.621150222	7.775997663	2.229830833	C	2.630582491	7.751625916	1.807342112
H	2.382860608	8.059330816	3.260446412	H	2.314769105	8.085453380	2.801321573
H	3.611214969	6.769179408	4.691631193	H	3.625309733	6.788730542	4.445619320
C	2.817672867	2.831466737	–0.026013739	C	2.995492186	2.976085975	0.366387201
H	2.722754072	2.779967969	1.066025159	H	2.980633180	2.978778510	1.463172266
C	2.133094455	2.081546171	–1.016742235	C	2.227586065	2.191500174	–0.535325659
H	1.378733941	1.302017579	–0.866093417	H	1.473266305	1.439040581	–0.282259058
C	2.621094976	2.550137638	–2.229963309	C	2.630243213	2.574637260	–1.807568344
H	2.382739316	2.266921332	–3.260595913	H	2.314316745	2.241007588	–2.801578629
H	3.611047434	3.557174342	–4.691721251	H	3.625050544	3.537679594	–4.445767631
N	5.195419543	5.078967859	–6.306037674	N	5.194493445	5.083014793	–6.060523254
N	6.055243795	5.904443225	–6.976057433	N	6.039688130	5.922750379	–6.730277598
C	4.427099302	4.318435139	–7.173095931	C	4.438660054	4.310507341	–6.926413153
C	4.812100896	4.672633213	–8.453132476	C	4.816752792	4.671296508	–8.207551169
C	5.826673382	5.661924364	–8.266171784	C	5.814207830	5.676934117	–8.021046220
H	6.397751906	6.205150140	–9.031015290	H	6.375251753	6.229571491	–8.786700987
H	4.417407517	4.274365402	–9.393779318	H	4.429002991	4.267512887	–9.148746615
H	3.679441954	3.599805644	–6.821841748	H	3.704325467	3.577882612	–6.575954237
N	5.195643687	5.247542369	6.306021891	N	5.194720419	5.243507846	6.060502927
N	6.055132135	4.421759452	6.976093002	N	6.039765665	4.403684209	6.730337738
C	4.427592826	6.008401790	7.173033153	C	4.438980016	6.016189358	6.926320151
C	4.812429839	5.654105440	8.453091799	C	4.816990808	5.655436595	8.207491977
C	5.826632078	4.664422230	8.266193133	C	5.814287417	4.649622088	8.021084054
H	6.397441185	4.120965442	9.031077318	H	6.375198040	4.096916739	8.786789990
H	4.417888966	6.052568176	9.393718751	H	4.429302178	6.059352543	9.148654431
H	3.680249433	6.727335940	6.821733373	H	3.704770797	6.748905761	6.575790388

$[Fe(bpp^{OH,H})_2]^{2+}$
53
$FeC_{22}N_{10}H_{18}O_2$

High-spin				Low-spin			
Fe	5.162787000	5.183717000	–0.002320000	Fe	5.137287213	5.164743875	0.000323466
N	5.143313000	5.179521000	–2.122268000	N	5.144026425	5.176762556	–1.890762251
N	6.672388000	6.637092000	–0.629848000	N	6.502813087	6.530260151	–0.315448770
C	5.965055000	5.988637000	–2.817667000	C	5.973762577	6.008546646	–2.559437980
C	6.005528000	6.025838000	–4.212700000	C	6.020877643	6.060708779	–3.952711259
H	6.689047000	6.689327000	–4.759430000	H	6.700526272	6.741485956	–4.483501169
C	5.121961000	5.164495000	–4.909688000	C	5.150015449	5.194553194	–4.664522357
N	6.781394000	6.789131000	–1.986286000	N	6.735905208	6.767456047	–1.657533462
C	7.699182000	7.758174000	–2.306728000	C	7.698854622	7.731109145	–1.828325669
H	7.924289000	8.021231000	–3.345663000	H	8.017639626	8.053325735	–2.825151782
C	8.199858000	8.252312000	–1.108526000	C	8.098891035	8.127210443	–0.558850921
H	8.953246000	9.037058000	–0.981837000	H	8.853595063	8.881321697	–0.311771983
C	7.528952000	7.519575000	–0.095933000	C	7.327352703	7.352923259	0.348571369
H	7.635634000	7.591705000	0.993805000	H	7.338570094	7.361033476	1.445358941
C	4.249206000	4.310700000	–4.188720000	C	4.276203102	4.319084038	–3.966441697
C	4.310466000	4.362178000	–2.797928000	C	4.316336338	4.352953822	–2.575462196
N	3.507505000	3.569535000	–1.945944000	N	3.548880526	3.583425945	–1.686919204
N	3.639659000	3.731118000	–0.592974000	N	3.773376855	3.803545910	–0.340990075
N	5.182984000	5.179581000	2.124474000	N	5.129756496	5.152673692	1.890642122
C	6.015473000	4.362657000	2.800571000	C	5.953522386	4.319905390	2.565546176
C	6.077420000	4.311005000	4.191417000	C	5.990268120	4.267932470	3.959167734
H	6.762700000	3.647414000	4.733243000	H	6.665264816	3.586585283	4.495089219
C	5.205343000	5.165051000	4.912786000	C	5.114604994	5.134533544	4.664364685
C	4.321899000	6.026352000	4.215719000	C	4.247117960	6.010986435	3.959849778
C	4.362067000	5.988493000	2.820592000	C	4.297878005	5.977470044	2.569197109
N	3.545613000	6.788493000	1.988759000	N	3.538207727	6.747981667	1.674887162
N	3.655427000	6.633914000	0.633199000	N	3.772545964	6.527360179	0.330654977
N	6.683920000	3.733787000	0.595835000	N	6.498807996	3.797835955	0.325578896
N	6.817836000	3.570120000	1.947838000	N	6.721484849	3.560081322	1.669388284
C	7.742554000	2.600290000	2.243329000	C	7.681990795	2.595266143	1.847262913
H	7.985875000	2.329630000	3.276147000	H	7.992772165	2.272354673	2.846392061
C	8.222343000	2.117455000	1.031691000	C	8.090990620	2.198803617	0.580758140
H	8.974532000	1.335121000	0.884535000	H	8.846559075	1.443767369	0.339218747
C	7.532099000	2.857537000	0.037875000	C	7.327426943	2.974327719	–0.332305579
H	7.618890000	2.794494000	–1.054214000	H	7.347012500	2.966386240	–1.428960455
C	2.799143000	7.515138000	0.096275000	C	2.949238729	7.358822485	–0.324084691
H	2.693161000	7.585066000	–0.993672000	H	2.939032285	7.380477237	–1.420668284
C	2.127572000	8.249662000	1.107021000	C	2.177370411	8.122217964	0.592128511
H	1.374179000	9.034080000	0.978311000	H	1.423483725	8.879962683	0.353881430
C	2.627537000	7.757965000	2.306679000	C	2.575821363	7.709809400	1.857000389
H	2.401863000	8.022930000	3.345001000	H	2.256616099	8.018886430	2.857865866
H	3.638533000	6.690329000	4.762087000	H	3.573026566	6.678456670	4.511448583
C	2.790540000	2.853962000	–0.038322000	C	2.943906857	2.973330058	0.307508902
H	2.702166000	2.789168000	1.053546000	H	2.925412820	2.951720857	1.403976013
C	2.101562000	2.115413000	–1.034258000	C	2.177763254	2.211222042	–0.614552416
H	1.349124000	1.332882000	–0.889471000	H	1.420810091	1.454739502	–0.382078996
C	2.583090000	2.600164000	–2.244292000	C	2.586411537	2.623058135	–1.876347173
H	2.341027000	2.331089000	–3.277825000	H	2.274284132	2.314524515	–2.879611139
H	3.564158000	3.647397000	–4.731150000	H	3.605589687	3.652269240	–4.523000698
O	5.263841000	5.110070000	6.247275000	O	5.059180965	5.172904073	6.001637153
H	4.628104000	5.737645000	6.651874000	H	5.691459271	4.535203081	6.395106363
O	5.063840000	5.109363000	–6.243912000	O	5.104563600	5.155588715	–6.002063461
H	5.699871000	5.736514000	–6.648782000	H	5.740059041	5.792710717	–6.391298640

$[Fe(bpp^{OMe,H})_2]^{2+}$
59
$FeC_{24}N_{10}H_{22}O_2$

High-spin				Low-spin			
Fe	5.267243000	5.162911000	0.000727000	Fe	5.257191161	5.189815204	–0.005115780
N	5.282093000	5.067568000	–2.120028000	N	5.274263058	5.110610258	–1.893617020
N	6.778122000	6.595215000	–0.664742000	N	6.588888842	6.574227786	–0.378492964
C	6.114989000	5.851647000	–2.836468000	C	6.089599576	5.932180683	–2.598764963
C	6.177232000	5.830409000	–4.225893000	C	6.145102140	5.915425922	–3.987400629
H	6.860719000	6.465464000	–4.802927000	H	6.803596025	6.578807950	–4.562157086
C	5.307773000	4.936625000	–4.907559000	C	5.303873040	4.990169544	–4.667962259
N	6.914293000	6.688827000	–2.023259000	N	6.824600552	6.753489504	–1.728365011
C	7.837219000	7.643945000	–2.366859000	C	7.761221556	7.734267639	–1.939773093
H	8.081919000	7.861604000	–3.411886000	H	8.076952050	8.017096789	–2.949481956
C	8.314329000	8.189858000	–1.181055000	C	8.141377950	8.201816022	–0.688354051
H	9.064464000	8.980558000	–1.073545000	H	8.871997394	8.988829700	–0.473784934
C	7.623840000	7.500873000	–0.151630000	C	7.385521560	7.450415589	0.250582567
H	7.707713000	7.620333000	0.935890000	H	7.388029180	7.511064382	1.345718183
C	4.424095000	4.110021000	–4.167379000	C	4.446712154	4.126594851	–3.934516801
C	4.465043000	4.223903000	–2.774586000	C	4.477249356	4.235182057	–2.542257217
N	3.647189000	3.466286000	–1.904723000	N	3.722529525	3.488809683	–1.623408877
N	3.751710000	3.690507000	–0.558107000	N	3.930125071	3.780356673	–0.287942487
N	5.282637000	5.258859000	2.122656000	N	5.242513394	5.267138745	1.883431990
C	6.115113000	4.474942000	2.839714000	C	6.085219007	4.492738972	2.599687405
C	6.177632000	4.496210000	4.229153000	C	6.123917049	4.505689612	3.996003010
H	6.861196000	3.860756000	4.805667000	H	6.822711477	3.862603769	4.543040407
C	5.308744000	5.390287000	4.911027000	C	5.224144605	5.383272430	4.658097918
C	4.425303000	6.216776000	4.170625000	C	4.334038569	6.201351396	3.906021601
C	4.466031000	6.102451000	2.777813000	C	4.386664312	6.102452882	2.520772469
N	3.648009000	6.859855000	1.907935000	N	3.609931443	6.808475063	1.587668014
N	3.752933000	6.634762000	0.561740000	N	3.857086857	6.531302930	0.256443313
N	6.776785000	3.731311000	0.668351000	N	6.652759123	3.876263575	0.389490546
N	6.914087000	3.637444000	2.026484000	N	6.877521685	3.711646340	1.743557404
C	7.837373000	2.682367000	2.368993000	C	7.865224090	2.785197228	1.970333963
H	8.083063000	2.464570000	3.413753000	H	8.183495523	2.521881797	2.984489597
C	8.313370000	2.136674000	1.182555000	C	8.289377047	2.338681263	0.725678976
H	9.063454000	1.346045000	1.074194000	H	9.067895011	1.595578965	0.523304945
C	7.622071000	2.825860000	0.153901000	C	7.506608995	3.045671862	–0.225846725
H	7.705115000	2.706584000	–0.933697000	H	7.529777105	2.985067808	–1.320708105
C	2.894842000	7.487011000	–0.017333000	C	3.013695906	7.306935689	–0.440106132
H	2.784521000	7.500024000	–1.109082000	H	3.008452254	7.276144623	–1.536550072
C	2.226735000	8.273410000	0.956192000	C	2.215991204	8.090427578	0.435971706
H	1.472609000	9.049934000	0.789417000	H	1.441298490	8.813277780	0.159189027
C	2.730757000	7.844665000	2.178116000	C	2.619974155	7.749717648	1.720469777
H	2.508733000	8.163459000	3.201879000	H	2.287086802	8.095781045	2.704638287
H	3.738062000	6.914568000	4.661817000	H	3.642552922	6.876450283	4.426131908
C	2.893754000	2.837433000	0.019694000	C	3.117364191	2.961083608	0.394632478
H	2.783181000	2.823597000	1.111412000	H	3.090271818	2.992608403	1.490722155
C	2.225996000	2.051575000	–0.954600000	C	2.379317762	2.134322829	–0.494159972
H	1.471982000	1.274753000	–0.788664000	H	1.641124550	1.369791062	–0.229859254
C	2.730335000	2.481322000	–2.175953000	C	2.787440251	2.494527295	–1.771747796
H	2.508715000	2.163173000	–3.200013000	H	2.492076140	2.127549206	–2.760250523
H	3.736794000	3.412631000	–4.659033000	H	3.786269613	3.402928480	–4.425990977
O	5.392026000	5.384369000	6.244475000	O	5.143470502	5.508365208	5.987726624
O	5.390932000	4.942682000	–6.240819000	O	5.387445497	5.003393172	–6.003216421
C	4.549494000	6.261845000	7.012168000	C	6.007206136	4.722725346	6.826162407
H	3.478028000	6.030437000	6.839863000	H	5.822090877	3.638402472	6.679093905
H	4.804143000	6.071442000	8.069394000	H	7.073104444	4.957751682	6.626022619
H	4.755276000	7.323389000	6.764053000	H	5.755337622	5.004476716	7.863412780
C	4.548214000	4.065544000	–7.008888000	C	4.575767842	4.100149100	–6.772351749
H	3.476826000	4.296958000	–6.836259000	H	4.817285504	3.045132230	–6.527439906
H	4.802775000	4.256533000	–8.066019000	H	3.496605670	4.293480267	–6.599976873
H	4.754111000	3.003922000	–6.761284000	H	4.824201949	4.299992266	–7.829447213

[Fe(bpp^{F,H})$_2$]$^{2+}$
51
$FeC_{22}N_{10}H_{16}F_2$

High-spin				Low-spin			
Fe	5.162907000	5.163005000	–0.006289000	Fe	5.162908848	5.162942994	–0.000007223
N	5.163029000	5.163062000	–2.128278000	N	5.163003129	5.163009550	–1.888678857
N	6.653033000	6.653401000	–0.598817000	N	6.527026549	6.526962584	–0.322563666
C	5.988062000	5.988256000	–2.798794000	C	5.994111764	5.993993292	–2.559718785
C	6.035551000	6.035805000	–4.196596000	C	6.037536569	6.037462764	–3.955827315
H	6.700891000	6.701564000	–4.759816000	H	6.705504767	6.705409505	–4.514146287
C	5.163543000	5.162860000	–4.867880000	C	5.163221579	5.163070374	–4.626911809
N	6.788247000	6.788460000	–1.957871000	N	6.756724385	6.756460885	–1.666991339
C	7.734030000	7.733737000	–2.274929000	C	7.720074976	7.719516805	–1.846046350
H	7.980870000	7.980249000	–3.313149000	H	8.036337458	8.035643393	–2.845712791
C	8.227451000	8.227474000	–1.075419000	C	8.122439487	8.121923810	–0.580288966
H	8.996335000	8.996342000	–0.944808000	H	8.877762923	8.877150885	–0.338731198
C	7.521931000	7.521920000	–0.065368000	C	7.353040187	7.352831509	0.334117627
H	7.609886000	7.609750000	1.025007000	H	7.368276074	7.368256475	1.430811030
C	4.291287000	4.290011000	–4.196790000	C	4.288800830	4.288633773	–3.955994961
C	4.338254000	4.337761000	–2.798978000	C	4.332014105	4.332052466	–2.559886823
N	3.537758000	3.537685000	–1.958237000	N	3.569274558	3.569514081	–1.667295712
N	3.672539000	3.672863000	–0.599152000	N	3.798775783	3.798909837	–0.322810204
N	5.163115000	5.163095000	2.130272000	N	5.163007096	5.163011733	1.888659361
C	5.984103000	4.342113000	2.813564000	C	5.994171363	4.332150482	2.559770762
C	6.033296000	4.293113000	4.211192000	C	6.037705122	4.288888612	3.955884479
H	6.701349000	3.624862000	4.768451000	H	6.705700147	3.621014213	4.514257564
C	5.163767000	5.163596000	4.887110000	C	5.163445406	5.163395260	4.626900890
C	4.293914000	6.033826000	4.211289000	C	4.289034757	6.037786853	3.955910907
C	4.342442000	5.984326000	2.813651000	C	4.332134095	5.994145149	2.559800798
N	3.543286000	6.782991000	1.971355000	N	3.569446884	6.756654791	1.667156766
N	3.690990000	6.634708000	0.619417000	N	3.798836960	6.527027291	0.322695764
N	6.634748000	3.691171000	0.619257000	N	6.527017425	3.798887272	0.322648984
N	6.782889000	3.543164000	1.971173000	N	6.756791716	3.569602751	1.667103999
C	7.730914000	2.595269000	2.267258000	C	7.720185116	2.606603510	1.846282706
H	7.988027000	2.338332000	3.300293000	H	8.036463929	2.290620371	2.845989728
C	8.211622000	2.114508000	1.055628000	C	8.122825359	2.204311264	0.580575932
H	8.979011000	1.347206000	0.908126000	H	8.878430570	1.449338517	0.339093797
C	7.497182000	2.828736000	0.060208000	C	7.353036205	2.972912319	–0.333903105
H	7.573576000	2.752344000	–1.031835000	H	7.368270666	2.957220316	–1.430584575
C	2.828229000	7.496883000	0.060477000	C	2.972842379	7.353046076	–0.333831717
H	2.751459000	7.573026000	–1.031557000	H	2.957345018	7.368506313	–1.430512867
C	2.113974000	8.211180000	1.055986000	C	2.203326579	8.121892498	0.580670781
H	1.346374000	8.978289000	0.908575000	H	1.447744958	8.876895985	0.339210745
C	2.595195000	7.730795000	2.267557000	C	2.606335315	7.719929909	1.846365406
H	2.338373000	7.987897000	3.300623000	H	2.290372770	8.036198209	2.846082368
H	3.626310000	6.702426000	4.768666000	H	3.621240240	6.705837114	4.514314269
C	2.803442000	2.804422000	–0.065903000	C	2.972678138	2.972924509	0.333638422
H	2.715136000	2.716712000	1.024453000	H	2.957224056	2.957317658	1.430318925
C	2.098167000	2.098855000	–1.076119000	C	2.203214013	2.204114491	–0.580936172
H	1.329180000	1.330057000	–0.945705000	H	1.447720751	1.448996610	–0.339573544
C	2.592036000	2.592417000	–2.275514000	C	2.605984453	2.606427107	–1.846596927
H	2.345504000	2.345836000	–3.313791000	H	2.289907833	2.290365684	–2.846343572
H	3.626158000	3.624188000	–4.760183000	H	3.620929941	3.620707577	–4.514454439
F	5.163767000	5.162776000	–6.197375000	F	5.163319998	5.163092306	–5.959291154
F	5.164099000	5.163857000	6.219754000	F	5.163622636	5.163558748	5.959277332

[Fe(bpp^{Cl,H})$_2$]$^{2+}$
51
$FeC_{22}N_{10}H_{16}Cl_2$

High-spin				Low-spin			
Fe	5.163074000	5.163014000	–0.004683000	Fe	5.162923472	5.162960043	–0.000007219
N	5.163092000	5.163053000	–2.127106000	N	5.163009060	5.163017702	–1.887396571
N	6.652509000	6.652396000	–0.598250000	N	6.527010073	6.526957559	–0.321995438
C	5.987459000	5.987281000	–2.798045000	C	5.993929690	5.993732756	–2.558737109
C	6.033581000	6.033626000	–4.196928000	C	6.035596205	6.035345791	–3.955567885
H	6.705373000	6.705797000	–4.743978000	H	6.710219771	6.709814607	–4.497123653
C	5.163177000	5.163113000	–4.882260000	C	5.163236946	5.163053029	–4.639731272
N	6.788104000	6.787589000	–1.957306000	N	6.756765415	6.756433659	–1.666026113
C	7.734201000	7.732904000	–2.273697000	C	7.720125086	7.719487071	–1.844829600
H	7.981624000	7.979795000	–3.311700000	H	8.036598804	8.035779550	–2.844371998
C	8.227507000	8.226058000	–1.073892000	C	8.122241005	8.121712932	–0.578856243
H	8.996643000	8.994591000	–0.942857000	H	8.877532722	8.876919600	–0.337148447
C	7.521413000	7.520769000	–0.064236000	C	7.352797828	7.352639814	0.335352209
H	7.608981000	7.608356000	1.026192000	H	7.367609483	7.367690883	1.432057781
C	4.292727000	4.292584000	–4.197010000	C	4.290765364	4.290736977	–3.955733445
C	4.338755000	4.338865000	–2.798130000	C	4.332211939	4.332324659	–2.558898682
N	3.538009000	3.538564000	–1.957489000	N	3.569246541	3.569568171	–1.666313453
N	3.673391000	3.673792000	–0.598419000	N	3.798816461	3.798956848	–0.322228328
N	5.163202000	5.163153000	2.127971000	N	5.163013433	5.163018400	1.887376778
C	5.983715000	4.343146000	2.812602000	C	5.993987621	4.332416020	2.558779720
C	6.031553000	4.295620000	4.210827000	C	6.035744133	4.290976982	3.955615062
H	6.706142000	3.621368000	4.752171000	H	6.710389734	3.616569868	4.497219525
C	5.163494000	5.163482000	4.901029000	C	5.163425913	5.163363149	4.639720776
C	4.295290000	6.031173000	4.210801000	C	4.290955362	6.035631676	3.955659509
C	4.342813000	5.983303000	2.812581000	C	4.332310681	5.993858496	2.558822047
N	3.543047000	6.782441000	1.970678000	N	3.569393750	6.756591891	1.666192161
N	3.691289000	6.634281000	0.618804000	N	3.798860509	6.526995068	0.322128799
N	6.634739000	3.691654000	0.618824000	N	6.527016659	3.798941419	0.322065363
N	6.783224000	3.543737000	1.970699000	N	6.756845160	3.569659108	1.666120453
C	7.731480000	2.595925000	2.266246000	C	7.720258625	2.606670798	1.845035946
H	7.989185000	2.338740000	3.299083000	H	8.036759203	2.290521710	2.844615661
C	8.211811000	2.115256000	1.054350000	C	8.122564308	2.204489016	0.579108271
H	8.979224000	1.348030000	0.906607000	H	8.878075411	1.449475760	0.337470804
C	7.497110000	2.829335000	0.059211000	C	7.352822524	2.973173002	–0.335163089
H	7.573096000	2.753071000	–1.032867000	H	7.367648187	2.957883040	–1.431856988
C	2.828636000	7.496314000	0.059193000	C	2.973054799	7.352787204	–0.335069986
H	2.752418000	7.572346000	–1.032885000	H	2.957978551	7.367864440	–1.431763529
C	2.113986000	8.210448000	1.054334000	C	2.203604307	8.121730872	0.579228883
H	1.346380000	8.977481000	0.906588000	H	1.448132873	8.876789421	0.337609200
C	2.594648000	7.730116000	2.266229000	C	2.606224974	7.719818419	1.845141872
H	2.337083000	7.987435000	3.299067000	H	2.290015748	8.036224158	2.844732731
H	3.620786000	6.705517000	4.752135000	H	3.616477480	6.710188602	4.497286723
C	2.804421000	2.805421000	–0.064517000	C	2.972921548	2.973191764	0.334901281
H	2.716673000	2.717859000	1.025898000	H	2.957891138	2.957976741	1.431594479
C	2.098498000	2.100089000	–1.074269000	C	2.203469735	2.204326930	–0.579460922
H	1.329371000	1.331528000	–0.943350000	H	1.448046626	1.449197717	–0.337927353
C	2.591965000	2.593235000	–2.274008000	C	2.605917205	2.606509715	–1.845344571
H	2.344698000	2.346328000	–3.312045000	H	2.289610119	2.290282798	–2.844961407
H	3.620947000	3.620463000	–4.744137000	H	3.616230274	3.616277054	–4.497409309
Cl	5.163208000	5.163156000	–6.611107000	Cl	5.163381511	5.163072055	–6.371508933
Cl	5.163675000	5.163685000	6.633241000	Cl	5.163644535	5.163539199	6.371494499

$[Fe(bpp^{Br,H})_2]^{2+}$
51
$FeC_{22}N_{10}H_{16}Br_2$

High-spin				Low-spin			
Fe	5.163050000	5.162990000	–0.004418000	Fe	5.162905971	5.162940403	–0.000007306
N	5.163079000	5.163052000	–2.127196000	N	5.162997306	5.163008987	–1.886689882
N	6.652216000	6.652127000	–0.597847000	N	6.527223998	6.527077418	–0.322215680
C	5.987395000	5.987324000	–2.797765000	C	5.993522792	5.993118728	–2.558703218
C	6.033336000	6.033562000	–4.197588000	C	6.035418398	6.034911067	–3.956094115
H	6.707622000	6.708339000	–4.738705000	H	6.712426936	6.711274311	–4.492867802
C	5.163166000	5.163167000	–4.882413000	C	5.163161719	5.163088860	–4.641187490
N	6.787956000	6.787551000	–1.956932000	N	6.756923894	6.756275438	–1.666431952
C	7.734146000	7.733018000	–2.272912000	C	7.720367563	7.719197489	–1.845106983
H	7.981914000	7.980335000	–3.310728000	H	8.037119452	8.035585594	–2.844534122
C	8.227251000	8.225872000	–1.072928000	C	8.122544948	8.121567930	–0.579224919
H	8.996366000	8.994377000	–0.941613000	H	8.877888464	8.876712051	–0.337503555
C	7.521019000	7.520417000	–0.063512000	C	7.353021276	7.352684096	0.335055430
H	7.608417000	7.607794000	1.026951000	H	7.368033215	7.368080716	1.431762811
C	4.292958000	4.292721000	–4.197706000	C	4.290854040	4.291206970	–3.956261367
C	4.338798000	4.338842000	–2.797881000	C	4.332590447	4.332934684	–2.558882538
N	3.538148000	3.538578000	–1.957177000	N	3.569088681	3.569679487	–1.666747771
N	3.673662000	3.674002000	–0.598071000	N	3.798586390	3.798774385	–0.322470625
N	5.163185000	5.163148000	2.127820000	N	5.162999328	5.163008826	1.886669869
C	5.983693000	4.343125000	2.812292000	C	5.993587802	4.333030303	2.558756851
C	6.031421000	4.295785000	4.211316000	C	6.035593214	4.291449552	3.956152630
H	6.708444000	3.619026000	4.746855000	H	6.712633878	3.615163440	4.492982413
C	5.163579000	5.163585000	4.900984000	C	5.163389815	5.163386555	4.641176971
C	4.295558000	6.031175000	4.211287000	C	4.291078486	6.035206898	3.956175489
C	4.342862000	5.983380000	2.812266000	C	4.332702512	5.993254994	2.558794648
N	3.543106000	6.782492000	1.970367000	N	3.569248803	6.756475203	1.666605446
N	3.691416000	6.634107000	0.618472000	N	3.798636946	6.527146957	0.322353393
N	6.634552000	3.691774000	0.618503000	N	6.527225307	3.798776980	0.322303080
N	6.783146000	3.543689000	1.970400000	N	6.757005137	3.569799837	1.666547332
C	7.731453000	2.595731000	2.265602000	C	7.720514420	2.606957863	1.845349307
H	7.989470000	2.338161000	3.298263000	H	8.037296022	2.290729992	2.844818183
C	8.211568000	2.115270000	1.053559000	C	8.122890720	2.204621079	0.579519151
H	8.978923000	1.348028000	0.905597000	H	8.878467581	1.449681966	0.337876120
C	7.496806000	2.829521000	0.058608000	C	7.353048673	2.973082149	–0.334835715
H	7.572648000	2.753426000	–1.033495000	H	7.368068686	2.957420750	–1.431531026
C	2.828840000	7.496030000	0.058572000	C	2.972864959	7.352913858	–0.334759742
H	2.752723000	7.571841000	–1.033532000	H	2.957581935	7.368343814	–1.431454875
C	2.114149000	8.210367000	1.053517000	C	2.203371662	8.121697022	0.579618421
H	1.346577000	8.977390000	0.905543000	H	1.447880989	8.876728929	0.337995815
C	2.594642000	7.730302000	2.265564000	C	2.606053225	7.719626177	1.845436020
H	2.336779000	7.988034000	3.298223000	H	2.289596943	8.036149389	2.844914576
H	3.618746000	6.708155000	4.746817000	H	3.614209711	6.711660687	4.493011210
C	2.804806000	2.805681000	–0.063879000	C	2.972723867	2.973026494	0.334561546
H	2.717221000	2.718292000	1.026567000	H	2.957489607	2.957441550	1.431256040
C	2.098770000	2.100193000	–1.073417000	C	2.203227978	2.204336271	–0.579890603
H	1.329685000	1.331635000	–0.942245000	H	1.447787891	1.449227215	–0.338367138
C	2.592027000	2.593092000	–2.273318000	C	2.605727544	2.606703884	–1.845673309
H	2.344423000	2.345784000	–3.311175000	H	2.289165647	2.290381505	–2.845182365
H	3.618774000	3.617927000	–4.738930000	H	3.613914808	3.614870784	–4.493155280
Br	5.163197000	5.163293000	–6.770034000	Br	5.163177098	5.163172077	–6.531431688
Br	5.163823000	5.163830000	6.791661000	Br	5.163510092	5.163566756	6.531417304

$[Fe(bpp^{I,H})_2]^{2+}$
51
$FeC_{22}N_{10}H_{16}I_2$

High-spin				Low-spin			
Fe	5.163038000	5.163022000	–0.004326000	Fe	5.162880064	5.162938971	–0.000007283
N	5.163075000	5.163049000	–2.126454000	N	5.162976251	5.163006295	–1.886177039
N	6.652160000	6.652058000	–0.598109000	N	6.527190403	6.527093640	–0.322424656
C	5.986952000	5.986714000	–2.797842000	C	5.992985488	5.992684716	–2.558845814
C	6.032282000	6.032101000	–4.198125000	C	6.034169899	6.033791067	–3.956664992
H	6.710091000	6.710151000	–4.730875000	H	6.714411138	6.713695926	–4.485032959
C	5.163261000	5.163029000	–4.888578000	C	5.163196159	5.163074956	–4.647274944
N	6.787896000	6.787361000	–1.957183000	N	6.756726517	6.756204216	–1.666640722
C	7.734145000	7.732725000	–2.273020000	C	7.720092881	7.719080045	–1.845281869
H	7.982160000	7.980148000	–3.310750000	H	8.036888176	8.035557879	–2.844661476
C	8.226857000	8.226073000	–1.072992000	C	8.122429437	8.121521450	–0.579392719
H	8.995694000	8.994858000	–0.941682000	H	8.877797526	8.876670431	–0.337761663
C	7.521059000	7.520274000	–0.063644000	C	7.353004936	7.352705054	0.334905097
H	7.608451000	7.607599000	1.026825000	H	7.368039347	7.368058856	1.431617010
C	4.294156000	4.293966000	–4.198228000	C	4.292122600	4.292308231	–3.956848857
C	4.339288000	4.339376000	–2.797941000	C	4.333095785	4.333356864	–2.559023063
N	3.538198000	3.538773000	–1.957387000	N	3.569245132	3.569740874	–1.666960872
N	3.673678000	3.674148000	–0.598295000	N	3.798569353	3.798752897	–0.322683010
N	5.163187000	5.163157000	2.126965000	N	5.162977989	5.163008018	1.886157139
C	5.983160000	4.343591000	2.812279000	C	5.993058565	4.333469213	2.558899605
C	6.030131000	4.296891000	4.211745000	C	6.034351283	4.292573105	3.956723443
H	6.710339000	3.616664000	4.738999000	H	6.714631415	3.612751427	4.485147727
C	5.163539000	5.163502000	4.907068000	C	5.163418941	5.163392305	4.647264774
C	4.296772000	6.029935000	4.211754000	C	4.292334953	6.034089974	3.956762923
C	4.343370000	5.982875000	2.812287000	C	4.333200282	5.992821972	2.558935242
N	3.543207000	6.782443000	1.970644000	N	3.569392081	6.756397489	1.666818476
N	3.691424000	6.634149000	0.618736000	N	3.798610417	6.527155486	0.322565803
N	6.634547000	3.691802000	0.618711000	N	6.527206111	3.798771879	0.322512290
N	6.783051000	3.543761000	1.970615000	N	6.756826767	3.569886952	1.666756138
C	7.731343000	2.595835000	2.265689000	C	7.720299370	2.607132114	1.845524145
H	7.989514000	2.338086000	3.298267000	H	8.037146584	2.290836407	2.844945392
C	8.211599000	2.115408000	1.053608000	C	8.122717309	2.204606526	0.579686922
H	8.979021000	1.348228000	0.905665000	H	8.878248966	1.449593001	0.338133594
C	7.496820000	2.829558000	0.058710000	C	7.353083874	2.973109744	–0.334685247
H	7.572640000	2.753510000	–1.033400000	H	7.368138783	2.957503725	–1.431385070
C	2.828883000	7.496135000	0.058759000	C	2.972788582	7.352898898	–0.334601817
H	2.752810000	7.571956000	–1.033350000	H	2.957464644	7.368272750	–1.431301394
C	2.114141000	8.210301000	1.053677000	C	2.203550835	8.121768578	0.579797810
H	1.346503000	8.977270000	0.905749000	H	1.448137535	8.876908591	0.338269644
C	2.594809000	7.730262000	2.265745000	C	2.606246596	7.719474253	1.845620360
H	2.336820000	7.988163000	3.298330000	H	2.289734167	8.036068915	2.845053141
H	3.616692000	6.710284000	4.739015000	H	3.612251200	6.714085223	4.485217127
C	2.804711000	2.805935000	–0.063944000	C	2.972668747	2.973016768	0.334403442
H	2.717126000	2.718661000	1.026513000	H	2.957399864	2.957481435	1.431102366
C	2.099013000	2.100169000	–1.073393000	C	2.203364462	2.204304399	–0.580070347
H	1.330114000	1.331426000	–0.942201000	H	1.447957004	1.449131881	–0.338641542
C	2.592017000	2.593380000	–2.273354000	C	2.605948613	2.606825041	–1.845857905
H	2.344192000	2.345907000	–3.311117000	H	2.289341497	2.290420391	–2.845321230
H	3.616408000	3.615924000	–4.731066000	H	3.611975480	3.612422748	–4.485361555
I	5.163413000	5.163086000	–6.992773000	I	5.163385758	5.163184173	–6.753714107
I	5.163861000	5.163714000	7.013861000	I	5.163716538	5.163571037	6.753701532

[Fe(bpp^{SH,H})$_2$]$^{2+}$
53
$FeC_{22}N_{10}H_{18}S_2$

High-spin				Low-spin			
Fe	5.168389000	5.164610000	–0.009231000	Fe	5.158239543	5.177662071	0.000203643
N	5.163423000	5.170777000	–2.119505000	N	5.139036780	5.180170883	–1.887058642
N	6.660701000	6.657792000	–0.600337000	N	6.514245898	6.546630168	–0.338264934
C	5.982294000	5.996077000	–2.798889000	C	5.957239067	6.011795393	–2.571545469
C	6.021929000	6.048552000	–4.194360000	C	5.984762264	6.055461062	–3.966306318
H	6.699250000	6.731900000	–4.722016000	H	6.659741790	6.740078867	–4.496401995
C	5.151391000	5.184206000	–4.904868000	C	5.109159012	5.185695015	–4.667189630
N	6.789557000	6.796263000	–1.958682000	N	6.728445368	6.778240239	–1.684391120
C	7.733967000	7.741485000	–2.276581000	C	7.685215672	7.745031289	–1.872107859
H	7.977307000	7.991636000	–3.314690000	H	7.989572952	8.064404481	–2.874369527
C	8.233671000	8.232056000	–1.077478000	C	8.100043794	8.148530647	–0.609909367
H	9.003702000	9.000133000	–0.948668000	H	8.854580628	8.906991306	–0.376106912
C	7.532329000	7.524096000	–0.066911000	C	7.343869648	7.375399532	0.311276632
H	7.624093000	7.607650000	1.023457000	H	7.369701939	7.388891376	1.407760920
C	4.286576000	4.312681000	–4.193077000	C	4.248778803	4.312186961	–3.948711216
C	4.337668000	4.351274000	–2.799275000	C	4.305487175	4.350302453	–2.556564178
N	3.537694000	3.543960000	–1.958999000	N	3.552891385	3.581813475	–1.655210056
N	3.676757000	3.671866000	–0.600791000	N	3.795223700	3.809764787	–0.313447218
N	5.167656000	5.159296000	2.123063000	N	5.177336456	5.175301851	1.887636614
C	5.984783000	4.339644000	2.812373000	C	6.014972624	4.349558184	2.555421802
C	6.029913000	4.287207000	4.208267000	C	6.070541910	4.305827809	3.949323185
H	6.711638000	3.602906000	4.729419000	H	6.759935244	3.625118770	4.465799566
C	5.163413000	5.148825000	4.924195000	C	5.205413361	5.171792574	4.667758781
C	4.298594000	6.016151000	4.209918000	C	4.325389627	6.039927617	3.966916724
C	4.347476000	5.974498000	2.815658000	C	4.352447171	6.000226645	2.573880941
N	3.546141000	6.778605000	1.974826000	N	3.576058409	6.762979500	1.688023930
N	3.695008000	6.636395000	0.623455000	N	3.792785825	6.536692339	0.341664596
N	6.642677000	3.691170000	0.617798000	N	6.529869862	3.817823758	0.311320318
N	6.788505000	3.540845000	1.968884000	N	6.773665227	3.588648180	1.652826268
C	7.736640000	2.593661000	2.263906000	C	7.741819194	2.629686617	1.821191298
H	7.993201000	2.334138000	3.296373000	H	8.069835815	2.314017969	2.817130905
C	8.220329000	2.115342000	1.051655000	C	8.133795028	2.228801103	0.550871630
H	8.988774000	1.349032000	0.904289000	H	8.889739856	1.476629818	0.301765820
C	7.506742000	2.830851000	0.057167000	C	7.352471116	2.995226014	–0.354810043
H	7.583786000	2.757817000	–1.035034000	H	7.355922715	2.981193671	–1.451601410
C	2.832225000	7.500129000	0.065922000	C	2.956640097	7.360267388	–0.306212717
H	2.757644000	7.579669000	–1.025981000	H	2.930983268	7.376228494	–1.402653393
C	2.116507000	8.209627000	1.063044000	C	2.193583324	8.124557667	0.616600130
H	1.348373000	8.976818000	0.918689000	H	1.432425315	8.876907797	0.384529594
C	2.597539000	7.723904000	2.273504000	C	2.611260983	7.721179625	1.877948779
H	2.338854000	7.977129000	3.307041000	H	2.303417846	8.034687810	2.881018635
H	3.615867000	6.695209000	4.737302000	H	3.647167132	6.717768191	4.502271393
C	2.810012000	2.800405000	–0.067528000	C	2.978816269	2.979645728	0.351063231
H	2.726623000	2.708155000	1.022799000	H	2.975655332	2.963161149	1.447816192
C	2.101706000	2.099887000	–1.078295000	C	2.203112969	2.209484300	–0.556215796
H	1.333453000	1.329980000	–0.949840000	H	1.453020591	1.450930285	–0.308794337
C	2.591948000	2.600405000	–2.277301000	C	2.591894488	2.616214607	–1.825710791
H	2.341349000	2.358183000	–3.315592000	H	2.266447721	2.300275287	–2.822434938
H	3.605146000	3.635968000	–4.724743000	H	3.563827197	3.630336657	–4.470333193
S	5.099064000	5.202971000	6.682219000	S	5.027272723	5.128046525	–6.424397857
S	5.081889000	5.131384000	–6.657563000	S	5.161226024	5.230986175	6.426337501
H	6.049141000	4.253730000	6.912354000	H	5.971230554	6.080299850	–6.666471916
H	6.031085000	6.081136000	–6.891306000	H	6.116028231	4.284750115	6.648646739

$[Fe(bpp^{SMe,H})_2]^{2+}$
59
$FeC_{24}N_{10}H_{22}S_2$

High-spin				Low-spin			
Fe	5.182547000	5.172280000	–0.000024000	Fe	5.134005667	5.241256357	0.007862993
N	5.169675000	5.210144000	–2.118095000	N	5.048291964	5.264440448	–1.877560928
N	6.690131000	6.638980000	–0.593876000	N	6.444676227	6.645805609	–0.362799210
C	5.985338000	6.039116000	–2.795500000	C	5.819365886	6.122554315	–2.581260461
C	6.025817000	6.110313000	–4.191063000	C	5.796749817	6.185700096	–3.976124317
H	6.714004000	6.797603000	–4.695737000	H	6.439017729	6.896953621	–4.508083187
C	5.149788000	5.264175000	–4.916395000	C	4.919169709	5.303202874	–4.659337088
N	6.797434000	6.825053000	–1.946131000	N	6.603678396	6.898134645	–1.712601273
C	7.710332000	7.806538000	–2.242495000	C	7.528919845	7.890488561	–1.922695223
H	7.933302000	8.097306000	–3.274342000	H	7.787932861	8.229760533	–2.931127973
C	8.208854000	8.273386000	–1.032592000	C	7.980219054	8.289315531	–0.671401374
H	8.958115000	9.058771000	–0.886836000	H	8.723903203	9.063764655	–0.455975518
C	7.542334000	7.512220000	–0.038155000	C	7.277458241	7.486951018	0.266790883
H	7.649510000	7.557802000	1.053010000	H	7.342637511	7.487964550	1.361678658
C	4.283077000	4.388163000	–4.206430000	C	4.106282623	4.399073758	–3.916447732
C	4.341425000	4.403844000	–2.814825000	C	4.210934902	4.421660953	–2.529088747
N	3.543431000	3.586250000	–1.981220000	N	3.509149579	3.625182259	–1.609365157
N	3.676720000	3.718441000	–0.625357000	N	3.792902137	3.845088453	–0.274725630
N	5.182752000	5.135736000	2.121574000	N	5.221002304	5.219957206	1.893496666
C	6.011295000	4.316899000	2.795611000	C	6.100769084	4.410535357	2.523061956
C	6.061370000	4.247351000	4.190990000	C	6.211230835	4.354679986	3.913903099
H	6.760444000	3.568128000	4.691657000	H	6.938754290	3.683605969	4.385052401
C	5.180682000	5.084229000	4.921295000	C	5.353840330	5.192154475	4.675196356
C	4.300290000	5.949782000	4.215564000	C	4.425916346	6.044745777	4.010695915
C	4.350614000	5.933347000	2.823618000	C	4.401748997	6.018878210	2.619524520
N	3.538944000	6.741711000	1.994212000	N	3.575329931	6.770869648	1.768571645
N	3.666657000	6.609035000	0.638331000	N	3.749530911	6.563372103	0.413495695
N	6.706921000	3.722799000	0.590673000	N	6.546711203	3.910806804	0.256330558
N	6.826541000	3.539083000	1.941773000	N	6.844570649	3.676080270	1.585700498
C	7.753071000	2.568620000	2.231878000	C	7.841334028	2.740296821	1.709780006
H	7.987297000	2.281694000	3.262279000	H	8.213101894	2.424940082	2.690267017
C	8.247917000	2.106846000	1.018398000	C	8.196650036	2.359856040	0.422328373
H	9.005526000	1.330512000	0.867328000	H	8.961521997	1.629154149	0.138920006
C	7.564954000	2.859192000	0.028516000	C	7.364289780	3.114420279	–0.447077643
H	7.664193000	2.813725000	–1.063401000	H	7.327707176	3.110103006	–1.543368641
C	2.810032000	7.491779000	0.103624000	C	2.870437416	7.371562282	–0.196237974
H	2.717790000	7.578086000	–0.986364000	H	2.805872104	7.396359204	–1.290832180
C	2.120385000	8.205058000	1.116875000	C	2.121557383	8.106846406	0.760711121
H	1.362557000	8.985637000	0.990910000	H	1.333016383	8.840816743	0.564012888
C	2.609592000	7.698871000	2.315257000	C	2.594113579	7.701837770	2.002334313
H	2.369147000	7.946450000	3.354587000	H	2.313910990	7.997220908	3.018939315
H	3.605963000	6.606673000	4.756196000	H	3.752460599	6.700843498	4.578569808
C	2.832842000	2.825155000	–0.088263000	C	3.021659624	2.987273095	0.408789248
H	2.747059000	2.736177000	1.002059000	H	3.058375655	2.959366899	1.504699472
C	2.146295000	2.105275000	–1.099047000	C	2.234303369	2.206972531	–0.479276670
H	1.398384000	1.315614000	–0.970512000	H	1.513786867	1.425924075	–0.214579256
C	2.623460000	2.618930000	–2.299009000	C	2.567770641	2.637357864	–1.757045792
H	2.380718000	2.369909000	–3.337467000	H	2.216103782	2.323527468	–2.745490212
H	3.592653000	3.724674000	–4.743979000	H	3.419855707	3.707217384	–4.423467167
S	5.091566000	5.135977000	6.670865000	S	4.753273040	5.239765064	–6.405738331
S	5.050260000	5.212252000	–6.664895000	S	5.352100043	5.258733081	6.429612543
C	6.323292000	3.915199000	7.219299000	C	6.638323030	4.070777449	6.922073768
H	6.269314000	3.941360000	8.324557000	H	6.644190038	4.107482984	8.028292071
H	6.064166000	2.897707000	6.868733000	H	6.384568254	3.043965009	6.595164775
H	7.343839000	4.201621000	6.899847000	H	7.633440204	4.375855373	6.545216511
C	6.263816000	6.448219000	–7.220101000	C	5.912789510	6.503559803	–7.010622136
H	7.289295000	6.172910000	–6.906254000	H	6.956532985	6.258122017	–6.734228386
H	6.205635000	6.421312000	–8.325063000	H	5.812131608	6.469934077	–8.112478327
H	5.994957000	7.461931000	–6.866266000	H	5.631098908	7.512561957	–6.652840651

[Fe(bpp^{SOMe,H})$_2$]$^{2+}$
61
$FeC_{24}N_{10}H_{22}S_2O_2$

High-spin				Low-spin			
Fe	5.181026224	5.258616883	0.005022200	Fe	5.183435282	5.261072497	0.004319601
N	5.070941029	5.408555011	–2.117082152	N	5.088161757	5.400998756	–1.874843498
N	6.551497647	6.853498204	–0.563248829	N	6.489600674	6.689740969	–0.276717205
C	5.820548997	6.317447136	–2.770854894	C	5.855922843	6.312351059	–2.519659209
C	5.780893599	6.472269115	–4.163643541	C	5.813703988	6.467828096	–3.909786336
H	6.383290666	7.233127083	–4.678275695	H	6.427790008	7.222686508	–4.420468819
C	4.921295171	5.604195266	–4.857501933	C	4.940057547	5.611158680	–4.604915592
N	6.630229255	7.087607151	–1.910742318	N	6.640080767	7.030285251	–1.607709211
C	7.547879756	8.069889609	–2.192966161	C	7.563207086	8.036375382	–1.758100108
H	7.748602024	8.396858507	–3.218696443	H	7.814872210	8.441634869	–2.743835690
C	8.082642802	8.482439185	–0.979435653	C	8.020419967	8.352608406	–0.486357164
H	8.844290293	9.253537133	–0.822272661	H	8.764409311	9.112154465	–0.224092641
C	7.427613853	7.695799684	0.002985167	C	7.324209075	7.489162934	0.401743056
H	7.553401571	7.709560539	1.093039322	H	7.398129721	7.419677485	1.493921219
C	4.122788239	4.658823050	–4.202422557	C	4.125278553	4.671359703	–3.955939538
C	4.244206135	4.600867715	–2.807785643	C	4.241888806	4.599394687	–2.564375555
N	3.527384354	3.708269917	–1.986687766	N	3.550177610	3.747441496	–1.695329976
N	3.750795773	3.745070314	–0.635698212	N	3.845208782	3.878787667	–0.351605354
N	5.284931180	5.119048931	2.128019614	N	5.276204456	5.126849127	1.884182917
C	6.154080916	4.268590987	2.707974850	C	6.164129144	4.276435737	2.453389331
C	6.262281392	4.126261700	4.098374219	C	6.269979270	4.133316629	3.841330520
H	6.961912298	3.412569655	4.554492442	H	6.982660802	3.428081070	4.291677929
C	5.420112143	4.943720094	4.870438693	C	5.412589580	4.936477470	4.616138852
C	4.498745665	5.826550689	4.294274657	C	4.475769425	5.812341585	4.047744053
C	4.477505764	5.877226727	2.894118266	C	4.449752233	5.877497694	2.650998945
N	3.624588532	6.710444594	2.144181025	N	3.620626457	6.672887170	1.851664833
N	3.720080830	6.677952075	0.778060272	N	3.793757116	6.551824765	0.486219417
N	6.697527706	3.758686562	0.440921907	N	6.601161703	3.922714620	0.160449600
N	6.923964055	3.544043513	1.774861201	N	6.903438137	3.605450774	1.471074633
C	7.926587419	2.625586723	1.968831824	C	7.903083027	2.665479590	1.533095776
H	8.248186397	2.324096198	2.971099072	H	8.277018792	2.288905614	2.490942039
C	8.364520742	2.235388966	0.709867068	C	8.254416621	2.368516051	0.223666235
H	9.155942330	1.513819932	0.480941015	H	9.019541917	1.659035186	–0.108544014
C	7.565948194	2.967982373	–0.205993321	C	7.417804026	3.174879596	–0.594217984
H	7.583742187	2.949995557	–1.303170112	H	7.378937127	3.238868296	–1.688667043
C	2.791434978	7.535004256	0.329950165	C	2.908785220	7.389952067	–0.070565660
H	2.667179649	7.698802047	–0.747711459	H	2.841046310	7.483634986	–1.161144558
C	2.085434519	8.128109579	1.408537638	C	2.156519705	8.058935418	0.932359393
H	1.274837292	8.862576443	1.355424025	H	1.362991790	8.798346072	0.781761446
C	2.644607769	7.580899791	2.555554390	C	2.632549343	7.581180799	2.145181208
H	2.423586641	7.741308379	3.616216044	H	2.352531332	7.807658282	3.179423860
H	3.843887811	6.425750900	4.945309869	H	3.810899221	6.398141251	4.701211760
C	2.935345134	2.820445620	–0.108653815	C	3.081558772	2.976774918	0.280024948
H	2.927433244	2.645570656	0.974602061	H	3.126785533	2.874703345	1.371201005
C	2.175626068	2.177748074	–1.120450551	C	2.287267132	2.255440511	–0.651930411
H	1.432713095	1.382612534	–0.997202783	H	1.570774489	1.456920814	–0.432545726
C	2.579842905	2.767927076	–2.310669622	C	2.607958881	2.769000855	–1.900806119
H	2.271373256	2.592952779	–3.346837005	H	2.248157056	2.522888087	–2.905440033
H	3.447090926	4.018767034	–4.790423806	H	3.438324843	4.044645899	–4.545969903
S	4.657063048	5.746897168	–6.681787871	S	4.678401997	5.766472733	–6.428966916
S	5.354031926	4.806812170	6.712895259	S	5.345713507	4.785902467	6.457664086
C	6.960130044	5.652771460	7.032549543	C	6.934455209	5.658013572	6.791864510
H	7.041875296	5.704426897	8.134903839	H	7.011449940	5.701412630	7.895007401
H	7.785476382	5.045248939	6.613909198	H	7.772946899	5.069977309	6.371418211
H	6.921181370	6.664204133	6.587130477	H	6.879231355	6.673034067	6.356155468
C	6.279960789	5.025920401	–7.176757107	C	6.277646108	5.000328604	–6.930681486
H	6.356988020	4.007801603	–6.751450219	H	6.326915179	3.979507990	–6.507796585
H	6.254507312	4.995474613	–8.282765790	H	6.248788798	4.972304938	–8.036744217
H	7.098235537	5.685483122	–6.828553810	H	7.115543917	5.635354015	–6.583354481
O	3.589173845	4.712863930	–7.024582324	O	3.581208096	4.762298561	–6.770751757
O	4.252329244	5.764027152	7.157727023	O	4.225456780	5.721693461	6.903918830

$[Fe(bpp^{SO2Me,H})_2]^{2+}$
63
$FeC_{24}N_{10}H_{22}S_2O_4$

High-spin				Low-spin			
Fe	5.184614145	5.170390286	0.001175615	Fe	5.137730072	5.238446429	0.007640543
N	5.171961689	5.213180322	–2.126390147	N	5.050375414	5.266799406	–1.874575580
N	6.688113307	6.632667278	–0.585901014	N	6.449427850	6.644721457	–0.353198499
C	5.996426150	6.043112055	–2.789217843	C	5.824326422	6.132126200	–2.568362741
C	6.034464180	6.114510430	–4.191712157	C	5.793996864	6.198693670	–3.968265583
H	6.719974476	6.799448613	–4.704463703	H	6.430482627	6.910583898	–4.507554822
C	5.154027431	5.269011806	–4.881605660	C	4.914253091	5.317029625	–4.617942435
N	6.805817859	6.819139654	–1.938919895	N	6.607765406	6.901726690	–1.702620898
C	7.727669612	7.796812758	–2.228963286	C	7.533984696	7.896033483	–1.909601476
H	7.960588325	8.086451448	–3.259133455	H	7.793325957	8.239487155	–2.916653601
C	8.219342563	8.258419984	–1.015952468	C	7.984119251	8.288808867	–0.657563353
H	8.972448723	9.038742750	–0.863422502	H	8.727641500	9.062276197	–0.438271492
C	7.541891702	7.500455444	–0.025379227	C	7.281489309	7.482809031	0.278818975
H	7.644146395	7.546510035	1.066296546	H	7.348559309	7.481806317	1.373673971
C	4.280857566	4.394004945	–4.210318138	C	4.101350387	4.407322019	–3.911727807
C	4.336641095	4.407906122	–2.812137493	C	4.208009437	4.423096879	–2.519521316
N	3.540300915	3.598240783	–1.980519468	N	3.510639843	3.626113345	–1.605675012
N	3.681996482	3.725337265	–0.623335721	N	3.796566929	3.841687939	–0.270388929
N	5.181844387	5.128394291	2.130080557	N	5.224696526	5.216284052	1.890002822
C	6.017636500	4.307370980	2.789722785	C	6.109215198	4.403247306	2.510658403
C	6.062014073	4.236248056	4.192036840	C	6.218200620	4.351633129	3.907187282
H	6.755796666	3.557531641	4.701948730	H	6.944199033	3.684063392	4.387062938
C	5.176460151	5.073412259	4.885561841	C	5.357753179	5.191491091	4.633844505
C	4.292378917	5.940076993	4.217732098	C	4.428640800	6.044331022	4.004492639
C	4.341609647	5.925484438	2.819328816	C	4.401919972	6.019218152	2.608521327
N	3.533146550	6.726278890	1.990793997	N	3.577539432	6.767766456	1.762078241
N	3.670698192	6.599943789	0.633223743	N	3.751269051	6.560229729	0.406607755
N	6.705258896	3.724670422	0.583597870	N	6.550834009	3.906996287	0.247862420
N	6.831798223	3.540108979	1.935979927	N	6.849889328	3.671154573	1.577291163
C	7.766561344	2.573561203	2.221787559	C	7.847614255	2.733793676	1.699464851
H	8.008210550	2.287450397	3.250922285	H	8.220994795	2.416807941	2.678957892
C	8.257715493	2.117366898	1.006472526	C	8.199759784	2.355832071	0.411964283
H	9.019593731	1.346290658	0.850490161	H	8.963644807	1.625230299	0.125774721
C	7.566392721	2.866775830	0.019014589	C	7.366449177	3.111711224	–0.456700761
H	7.663757187	2.821329600	–1.073137777	H	7.330130775	3.106570279	–1.553072054
C	2.811633197	7.479545965	0.098955916	C	2.871335066	7.365825265	–0.202612859
H	2.723093375	7.571551031	–0.990941950	H	2.804189178	7.391782296	–1.297106820
C	2.111303518	8.184427563	1.112027099	C	2.121495148	8.100408887	0.755431133
H	1.349085433	8.960185086	0.983426917	H	1.331721678	8.832653022	0.557478897
C	2.594630945	7.677605121	2.310601908	C	2.593728931	7.698174303	1.996420512
H	2.345314653	7.917784121	3.349718634	H	2.313433121	7.993265320	3.013210827
H	3.602633725	6.592345377	4.769465552	H	3.759281188	6.695936788	4.582027891
C	2.835039276	2.835807979	–0.086264977	C	3.027734398	2.982208417	0.411271618
H	2.752000350	2.742129522	1.003935421	H	3.064436143	2.949272524	1.507117439
C	2.138680197	2.123523217	–1.096930206	C	2.238920575	2.204117214	–0.478872352
H	1.386070497	1.338878632	–0.965707437	H	1.519552290	1.422094821	–0.214083563
C	2.611275015	2.636540269	–2.297120478	C	2.568365353	2.637206080	–1.755157898
H	2.360688602	2.393930586	–3.335362262	H	2.214990755	2.326487149	–2.744118924
H	3.593728369	3.736728411	–4.759376746	H	3.418089966	3.720846877	–4.429971022
S	5.064302277	5.140590424	6.711395611	S	4.711901173	5.249173227	–6.434320000
S	5.039367556	5.196688845	–6.707130626	S	5.343552942	5.283719137	6.460451582
C	6.270055994	3.962874474	7.329272002	C	6.577756417	4.109767338	7.027777948
H	6.168134111	4.035939313	8.429602000	H	6.533618808	4.195424767	8.130956563
H	6.004424852	2.947031969	6.985050522	H	6.293194595	3.090766587	6.708767694
H	7.283414701	4.272225149	7.015236127	H	7.573977880	4.413403802	6.658768494
C	6.205477671	6.411577412	–7.329041904	C	5.853018018	6.460589562	–7.108228193
H	7.229967755	6.132030292	–7.023172176	H	6.889121444	6.180366543	–6.844625863
H	6.098235751	6.338831001	–8.428886031	H	5.698940079	6.384392370	–8.202288054
H	5.911677307	7.417851625	–6.979526530	H	5.575784360	7.468938573	–6.751495765
O	3.698233077	4.670093465	7.054791608	O	3.318387126	5.682505265	–6.709516933
O	5.477741528	6.513872391	7.096344887	O	5.142246166	3.888594057	–6.845709009
O	5.494443470	3.836783086	–7.093005169	O	5.781282358	6.660158300	6.806582543
O	3.658190954	5.624090374	–7.046314245	O	3.996849737	4.823292220	6.884935743

$[Fe(bpp^{H,Me})_2]^{2+}$
63
$FeC_{26}N_{10}H_{26}$

High-spin				Low-spin			
Fe	5.161982001	5.153199225	–0.004546062	Fe	5.162861218	5.164428499	–0.000007302
N	5.146786231	5.166158724	–2.133645258	N	5.152109521	5.164575377	–1.887831016
N	6.608379997	6.667247474	–0.614642833	N	6.525472302	6.527622555	–0.328851210
C	5.954018488	6.004865838	–2.810240070	C	5.980725712	5.995866439	–2.562071287
C	5.981402044	6.058927197	–4.211856449	C	6.009526093	6.033187599	–3.961900967
H	6.641307820	6.750122820	–4.751441049	H	6.683314576	6.709538228	–4.503823824
C	5.123562042	5.185208173	–4.901916316	C	5.136480105	5.165379875	–4.645503252
H	5.114050734	5.192931443	–6.002064475	H	5.130047729	5.165766730	–5.745501411
N	6.749947305	6.806302117	–1.967666100	N	6.747740626	6.756273452	–1.671297465
C	7.713040179	7.739186439	–2.270917583	C	7.713557812	7.718442953	–1.850762320
H	7.966796448	7.988105227	–3.307005371	H	8.024255177	8.034796906	–2.852312446
C	8.220983129	8.225606507	–1.067796510	C	8.133080110	8.129254915	–0.587950402
C	7.488298854	7.517681093	–0.068947426	C	7.355877910	7.350717678	0.324321832
H	7.565876982	7.600146026	1.023088168	H	7.373002058	7.363650406	1.421501689
C	4.277600197	4.301671823	–4.209641097	C	4.271366143	4.297033513	–3.952524646
C	4.328621243	4.336378427	–2.808078088	C	4.316119406	4.333503831	–2.553094697
N	3.547724688	3.522500765	–1.963312780	N	3.559534534	3.572250407	–1.654049578
N	3.713292393	3.641410819	–0.610979626	N	3.796931459	3.800136092	–0.314055065
N	5.179683632	5.167010812	2.123876006	N	5.173913684	5.164619123	1.887812005
C	5.998744196	4.337560685	2.797519859	C	6.010199643	4.333715217	2.552906056
C	6.052332002	4.304288892	4.198938495	C	6.055530177	4.297544907	3.952314384
H	6.721613446	3.620841680	4.736833227	H	6.735704600	3.621638513	4.486800657
C	5.208101952	5.189151958	4.891765701	C	5.190735504	5.166039268	4.645482782
C	4.349371854	6.062596265	4.202385836	C	4.317435334	6.033762912	3.962070593
C	4.374089797	6.006858250	2.800844835	C	4.345661019	5.996133615	2.562223264
N	3.576802024	6.807723510	1.958776017	N	3.578456074	6.756511182	1.671562368
N	3.715744502	6.667252950	0.605588177	N	3.800307323	6.527663220	0.329071750
N	6.610839688	3.640666025	0.599899321	N	6.528769206	3.800092405	0.313810601
N	6.778060170	3.522536220	1.952115558	N	6.766541228	3.572373312	1.653754527
C	7.745028075	2.592454305	2.251565506	C	7.732157544	2.608212250	1.822786716
H	8.018568687	2.360005781	3.286437421	H	8.055131876	2.292992478	2.820805981
C	8.228821500	2.085956135	1.046745495	C	8.135931356	2.195191617	0.555525517
C	7.478489861	2.779719453	0.051062612	C	7.349699760	2.974573347	–0.348218537
H	7.534874745	2.679822706	–1.040848932	H	7.354813846	2.961456641	–1.445502181
C	2.835076430	7.517451176	0.060861597	C	2.969788367	7.350787465	–0.323924114
H	2.755479808	7.598880361	–1.031102450	H	2.952284135	7.363641490	–1.421090212
C	2.104519343	8.226675924	1.060412878	C	2.192943192	8.129527599	0.588466915
C	2.614661843	7.741313101	2.263023171	C	2.612811543	7.718825137	1.851202352
H	2.363075890	7.991292902	3.299383081	H	2.302447187	8.035335790	2.852806831
H	3.691192733	6.754696925	4.742912336	H	3.643924230	6.710305775	4.504106825
H	5.219724883	5.198287658	5.991879370	H	5.197571029	5.166758172	5.745478093
C	2.844165327	2.781597096	–0.062554674	C	2.975831130	2.974679961	0.347841088
H	2.786464289	2.682402087	1.029343822	H	2.970416065	2.961702426	1.445125506
C	2.094636653	2.087766835	–1.058724772	C	2.189875530	2.195162383	–0.556024884
C	2.580368345	2.593030950	–2.263277540	C	2.593991603	2.608035157	–1.823228910
H	2.307979412	2.359878605	–3.298298094	H	2.271286323	2.292693498	–2.821296935
H	3.608839768	3.617769809	–4.747612174	H	3.591418532	3.620986025	–4.487129640
C	1.033803397	1.048163033	–0.853430772	C	1.155888731	9.158143750	0.250006616
H	0.612298522	0.712870204	–1.819260352	H	1.586957868	9.974476938	–0.365393097
H	1.441651397	0.159054703	–0.329990982	H	0.325495936	8.710823105	–0.334400372
H	0.203325747	1.441910696	–0.232278231	H	0.728038072	9.606535420	1.165832567
C	9.288366634	9.259079228	–0.865579814	C	9.165780311	1.163066088	0.205703627
H	8.903494417	10.123060147	–0.285899912	H	9.611671034	0.722850737	1.116930055
H	10.146895420	8.841442291	–0.300483489	H	8.721093521	0.340947202	–0.392067820
H	9.666242098	9.636444368	–1.833921309	H	9.984198013	1.603981042	–0.399969765
C	1.036665638	9.259833539	0.859106158	C	9.170130801	9.157823698	–0.249333567
H	1.418648037	10.120277340	0.272328027	H	8.738849962	9.974519687	0.365438774
H	0.174393824	8.839686414	0.301606427	H	10.000088106	8.710612161	0.335776365
H	0.664835618	9.642676581	1.827639589	H	9.598610002	9.605732489	–1.165102624
C	9.288636385	1.045497083	0.840618137	C	1.159948735	1.163062148	–0.206350667
H	9.708869187	0.707906624	1.806197977	H	0.714397880	0.722632970	–1.117639854
H	8.880281205	0.157877571	0.315070893	H	1.604459531	0.341093710	0.391758425
H	10.120193924	1.439530962	0.221065981	H	0.341296345	1.604072423	0.398935974

[Fe(bpp^{H,CH2OH})$_2$]$^{2+}$
67
$FeC_{26}N_{10}H_{26}O_4$

High-spin				Low-spin			
Fe	5.169396000	5.197088000	0.044426000	Fe	5.197175119	5.183905859	–0.011762293
N	5.178901000	5.147916000	–2.085040000	N	5.175283283	5.174261882	–1.899354788
N	6.681820000	6.634704000	–0.589967000	N	6.577426328	6.525412141	–0.355339033
C	6.013680000	5.946490000	–2.775793000	C	6.010662940	5.990126357	–2.583673768
C	6.063345000	5.954003000	–4.177500000	C	6.030421797	6.021203504	–3.983469685
H	6.750351000	6.608007000	–4.729455000	H	6.710781838	6.684670329	–4.533059389
C	5.190437000	5.083018000	–4.852200000	C	5.139244519	5.164210238	–4.657403830
H	5.195472000	5.056797000	–5.952057000	H	5.125026338	5.160004379	–5.757280718
N	6.816788000	6.756269000	–1.946309000	N	6.796753328	6.742706610	–1.701098332
C	7.755787000	7.705606000	–2.268867000	C	7.771546300	7.692019654	–1.893345584
H	8.009175000	7.935583000	–3.309393000	H	8.079073625	7.999903737	–2.898528953
C	8.237932000	8.230938000	–1.073214000	C	8.205377563	8.094204904	–0.633294492
C	7.535216000	7.520692000	–0.058911000	C	7.424364094	7.337809222	0.289685734
H	7.630425000	7.614478000	1.029673000	H	7.449098176	7.366156979	1.385662683
C	4.311404000	4.244869000	–4.144869000	C	4.265487423	4.312915823	–3.954239425
C	4.349560000	4.318046000	–2.744698000	C	4.321448342	4.354604089	–2.555640737
N	3.538830000	3.547787000	–1.886082000	N	3.557102969	3.610451198	–1.648022726
N	3.666631000	3.727713000	–0.536339000	N	3.810695257	3.836881855	–0.310598775
N	5.157167000	5.240933000	2.174837000	N	5.220250826	5.200571983	1.876152007
C	5.989577000	4.445357000	2.872130000	C	6.052714451	4.368632206	2.543922645
C	6.027387000	4.435400000	4.274186000	C	6.108590260	4.345463809	3.942865634
H	6.713202000	3.783158000	4.829710000	H	6.786265866	3.667893285	4.478438239
C	5.145096000	5.301256000	4.942896000	C	5.258022960	5.229902595	4.633750303
C	4.269230000	6.137387000	4.228965000	C	4.389335588	6.100176374	3.947848237
C	4.319416000	6.066610000	2.829165000	C	4.407091490	6.048343491	2.548397768
N	3.514618000	6.836054000	1.963530000	N	3.641622058	6.809037525	1.656406652
N	3.651959000	6.653836000	0.614433000	N	3.852418943	6.566131662	0.314671851
N	6.685993000	3.769101000	0.693246000	N	6.548659932	3.805832320	0.306314669
N	6.804165000	3.640727000	2.049544000	N	6.796576163	3.591444194	1.646661309
C	7.738638000	2.689482000	2.379090000	C	7.751513973	2.619269535	1.820844667
H	7.980974000	2.443885000	3.417905000	H	8.084936834	2.306011541	2.815202515
C	8.247609000	2.180887000	1.187344000	C	8.131836877	2.187353459	0.554255616
C	7.555698000	2.894296000	0.167593000	C	7.350225380	2.959266873	–0.355222788
H	7.659121000	2.810286000	–0.921946000	H	7.342104162	2.930704657	–1.452015311
C	2.797321000	7.512925000	0.042503000	C	3.028822280	7.394144130	–0.342699206
H	2.702863000	7.557420000	–1.049343000	H	3.005522018	7.397918091	–1.439618551
C	2.091564000	8.266547000	1.022714000	C	2.271535253	8.183101060	0.571655350
C	2.573142000	7.796762000	2.241673000	C	2.689088656	7.783754280	1.836468424
H	2.318218000	8.073038000	3.270414000	H	2.383961750	8.121428065	2.831301084
H	3.579890000	6.813565000	4.750538000	H	3.727170940	6.790038057	4.487108534
H	5.140156000	5.325717000	6.042778000	H	5.273113973	5.241674873	5.733571953
C	2.817521000	2.857506000	0.028807000	C	2.985121221	3.023146495	0.362306311
H	2.728323000	2.808004000	1.121795000	H	2.991953124	3.010220634	1.459420030
C	2.124477000	2.099853000	–0.958019000	C	2.181448678	2.261363254	–0.536644019
C	2.612631000	2.574464000	–2.172118000	C	2.574257636	2.663884771	–1.808939563
H	2.369366000	2.283865000	–3.198978000	H	2.231211122	2.347153067	–2.798902872
H	3.628498000	3.565335000	–4.670507000	H	3.570317450	3.645708897	–4.480397671
C	1.069244000	1.036422000	–0.773563000	C	1.228825705	9.220234804	0.268484575
H	0.106091000	1.523144000	–0.506844000	H	1.630850065	9.929721884	–0.496106384
H	1.341782000	0.390830000	0.093596000	H	0.350053746	8.719428569	–0.205716346
C	9.253662000	9.319326000	–0.871071000	C	9.177010190	1.140928629	0.248367671
H	9.864704000	9.427207000	–1.798535000	H	10.104092547	1.645826394	–0.099939606
H	8.716045000	10.288135000	–0.732353000	H	8.831055955	0.507266342	–0.601606586
C	1.074038000	9.342704000	0.769913000	C	9.278732605	9.093527543	–0.274621906
H	0.462957000	9.493210000	1.691353000	H	10.231779303	8.552884451	–0.089589156
H	1.610391000	10.304681000	0.585916000	H	9.461072921	9.769538405	–1.141309626
C	9.291098000	1.098525000	1.053258000	C	1.104585958	1.252213128	–0.215822327
H	8.791204000	0.143688000	0.780949000	H	0.197974130	1.790522646	0.136585945
H	9.973214000	1.344959000	0.206697000	H	1.435851442	0.612720657	0.635570366
O	9.988408000	0.857145000	2.263528000	O	0.711723950	0.494739977	–1.346542540
H	10.555964000	1.634412000	2.436919000	H	1.445194219	–0.114214570	–1.563705143
O	0.830328000	0.290825000	–1.955301000	O	9.535583959	0.378259985	1.387153387
H	1.617655000	–0.266456000	–2.116049000	H	8.777290987	–0.198558508	1.607111146

O	10.043222000	8.997860000	0.266373000	O	8.988884646	9.799636445	0.922297031
H	10.528799000	9.806634000	0.518851000	H	8.207066847	10.361328712	0.749888543
O	0.285063000	8.967068000	–0.351166000	O	0.880996051	9.869730720	1.478354076
H	–0.199503000	9.763036000	–0.643094000	H	0.109047467	10.438269696	1.295302138

$[Fe(bpp^{H,tBu})_2]^{2+}$
99
$FeC_{38}N_{10}H_{50}$

High-spin				Low-spin			
Fe	5.163267854	5.163594185	–0.003530769	Fe	5.166633622	5.162876994	–0.000004761
N	5.164682304	5.155654348	–2.128165620	N	5.166858927	5.152801607	–1.887978300
N	6.657253202	6.648801596	–0.601417050	N	6.530181290	6.524971256	–0.329180102
C	5.989934171	5.979330395	–2.798811316	C	5.998965441	5.980964486	–2.562077991
C	6.029357830	6.016490214	–4.201471505	C	6.036186949	6.010065239	–3.961952398
H	6.703480179	6.689463725	–4.746168044	H	6.713043158	6.683747517	–4.503403953
C	5.161992284	5.148243614	–4.886081712	C	5.168262977	5.137864793	–4.646281055
H	5.160901204	5.145141899	–5.986422836	H	5.168890476	5.131763304	–5.746250363
N	6.789745924	6.779619309	–1.958666137	N	6.760065765	6.747048699	–1.671166354
C	7.736295643	7.725806304	–2.275698897	C	7.724601518	7.710839120	–1.849748760
H	7.974333779	7.962327436	–3.317358840	H	8.035254786	8.015638675	–2.853757977
C	8.242135990	8.233942533	–1.081118391	C	8.136410643	8.130389616	–0.586708934
C	7.526143931	7.518345232	–0.072676862	C	7.354120913	7.353239859	0.324224124
H	7.606251681	7.599632081	1.019288009	H	7.357939541	7.363780276	1.421388566
C	4.295662203	4.283352829	–4.194794377	C	4.299376212	4.272965402	–3.953358358
C	4.338513325	4.328825126	–2.793232948	C	4.335913118	4.317835731	–2.554345610
N	3.540154805	3.532325944	–1.945199027	N	3.573671029	3.560552000	–1.654705145
N	3.677106535	3.671329621	–0.590105508	N	3.802417262	3.797195228	–0.316178033
N	5.164247778	5.172233343	2.130267759	N	5.166929181	5.173138786	1.887963676
C	5.986238386	4.353560862	2.815502250	C	5.999161245	4.345170536	2.562133191
C	6.028605570	4.316647456	4.216974032	C	6.036575508	4.316360929	3.962008937
H	6.706037636	3.641650550	4.755493063	H	6.713529765	3.642822543	4.503516214
C	5.164474133	5.183225674	4.908013280	C	5.168715052	5.188683560	4.646267589
C	4.299939529	6.044591234	4.210091294	C	4.299745062	6.053442932	3.953274989
C	4.342839271	5.995876859	2.809637454	C	4.336097833	6.008277793	2.554263114
N	3.544313937	6.790217054	1.960302084	N	3.573825092	6.765447608	1.654564726
N	3.692630113	6.636339760	0.612476346	N	3.802432008	6.528528722	0.316066443
N	6.637051331	3.695058423	0.624112736	N	6.530239669	3.800785634	0.329271867
N	6.784075395	3.553054538	1.974229409	N	6.760245110	3.578981849	1.671284210
C	7.732685014	2.604212167	2.270277903	C	7.724884671	2.615318021	1.849999497
H	7.982234757	2.358180273	3.306889127	H	8.035627301	2.310743073	2.854049144
C	8.223899602	2.106985941	1.063230628	C	8.136659317	2.195552878	0.587018104
C	7.498334393	2.830337893	0.069586626	C	7.354227021	2.972448981	–0.323997400
H	7.565494003	2.759032166	–1.023839045	H	7.358019780	2.961643690	–1.421151773
C	2.829028309	7.497421754	0.050330542	C	2.975447016	7.351256677	–0.345219916
H	2.759983478	7.561356666	–1.042008715	H	2.968108971	7.351733915	–1.441191130
C	2.103862479	8.227108871	1.035145546	C	2.195854308	8.136711370	0.556271593
C	2.596792776	7.738727330	2.248883429	C	2.611486846	7.729055415	1.825569607
H	2.348195088	7.991369722	3.285024631	H	2.303951820	8.042884385	2.828563044
H	3.622658068	6.723821703	4.743433426	H	3.623574900	6.733241320	4.487901252
H	5.164547552	5.187688727	6.008057898	H	5.169490541	5.195018293	5.746235431
C	2.808455069	2.802456996	–0.054135635	C	2.975430957	2.974392918	0.345017642
H	2.729353878	2.725325739	1.036752478	H	2.968191678	2.973702121	1.440989897
C	2.091136337	2.082451542	–1.054161869	C	2.195696501	2.189172240	–0.556555664
C	2.595208946	2.585839847	–2.255192651	C	2.611237687	2.597050624	–1.825813606
H	2.356299992	2.345086816	–3.296581757	H	2.303600147	2.283434693	–2.828843047
H	3.620646198	3.607365637	–4.734601598	H	3.623131418	3.593287916	–4.488043380
C	1.021298491	1.011154637	–0.872790219	C	1.142784220	9.188405790	0.221317696
C	0.769877532	0.762258687	0.629738571	C	0.992697103	9.324514840	–1.308584435
H	1.686973368	0.408428419	1.145289966	H	1.942171358	9.642622817	–1.787336161
H	–0.008571500	–0.017373726	0.759106528	H	0.226024135	10.090197720	–1.544644431
H	0.413342298	1.679720184	1.142760255	H	0.668838190	8.371176514	–1.775655289
C	1.498119353	–0.304534962	–1.538999306	C	1.576058414	10.551437594	0.818940054
H	2.441437455	–0.666433203	–1.081292922	H	2.549829808	10.880922779	0.402217835
H	1.673777965	–0.170097684	–2.626184679	H	1.675288913	10.497606627	1.922648108
H	0.729892467	–1.096076119	–1.413963522	H	0.820928616	11.329498298	0.581813723
C	–0.293892078	1.484297693	–1.542534909	C	–0.215354471	8.763581389	0.835718239
H	–0.658399528	2.427888441	–1.087446371	H	–0.550659753	7.786534277	0.431454950

H	–1.084348585	0.714935899	–1.417563325	H	–0.994177644	9.517965032	0.598690667
H	–0.157697743	1.658058789	–2.629821158	H	–0.151549908	8.674754193	1.939799700
C	9.287358186	1.039939147	0.839817781	C	9.180991378	9.178306182	–0.228489088
C	9.813604702	0.519446704	2.193886682	C	9.795164191	9.783309907	–1.508540871
H	10.276470341	1.332897977	2.790726152	H	10.299226775	9.009728580	–2.124706069
H	10.587301594	–0.257144632	2.027180816	H	10.554298954	10.544980296	–1.238586131
H	9.002126518	0.061769597	2.797377249	H	9.026008770	10.282276621	–2.134313882
C	10.458748907	1.653974749	0.031527575	C	10.299323032	8.512332258	0.613599019
H	10.922160063	2.501269815	0.577338920	H	10.802608787	7.704042194	0.044412243
H	10.121060936	2.025945652	–0.957315946	H	9.901432622	8.075359075	1.552193999
H	11.241691907	0.887976281	–0.146790719	H	11.063846927	9.266867042	0.891043359
C	8.666141820	–0.134115821	0.040889945	C	8.507610299	10.302185272	0.600220946
H	7.820537449	–0.592441919	0.593561637	H	7.703012267	10.800259163	0.021312552
H	9.429276233	–0.919844562	–0.137306037	H	9.259248538	11.069509003	0.877713840
H	8.289870792	0.199442037	–0.947722435	H	8.064073008	9.910463165	1.538308461
C	1.035065807	9.295553210	0.831123689	C	1.142561897	1.137504442	–0.221723494
C	0.796465156	9.526633357	–0.676255904	C	0.992715129	1.000978249	1.308164468
H	1.717795559	9.874964154	–1.187998534	H	1.942239480	0.682652402	1.786671232
H	0.018739602	10.304192818	–0.821383955	H	0.226009312	0.235300116	1.544134111
H	0.444880810	8.602964130	–1.181500123	H	0.669016143	1.954216497	1.775550424
C	1.505122673	10.619465111	1.485791970	C	1.575562374	–0.225404211	–0.819816888
H	2.452231701	10.976585708	1.032141674	H	2.549368096	–0.555133687	–0.403367085
H	1.671412783	10.497947300	2.575962422	H	1.674600992	–0.171273047	–1.923527806
H	0.737661848	11.409009026	1.344542321	H	0.820379872	–1.003441141	–0.582778506
C	–0.285825405	8.829978895	1.494921465	C	–0.215628189	1.562679634	–0.835775410
H	–0.646094507	7.880876029	1.047941433	H	–0.550740266	2.539650746	–0.431170173
H	–1.075474962	9.597376463	1.353834259	H	–0.994504481	0.808323023	–0.598837013
H	–0.159032418	8.669091556	2.585326620	H	–0.151991558	1.651821196	–1.939840462
C	9.308548145	9.301922844	–0.879738066	C	9.181327270	1.147665422	0.228968372
C	9.819082217	9.810279525	–2.244164836	C	9.795631363	0.543015421	1.509123535
H	10.274330393	8.991317143	–2.839334080	H	10.299658070	1.316787892	2.125077385
H	10.595267247	10.587692652	–2.093532684	H	10.554813006	–0.218651137	1.239292580
H	9.000798245	10.263361506	–2.841913717	H	9.026563579	0.044120652	2.135062062
C	10.488255868	8.693127018	–0.079529862	C	10.299545910	1.813559172	–0.613329239
H	10.944814587	7.841063561	–0.623643908	H	10.802793038	2.622018837	–0.044348955
H	10.161098258	8.329494998	0.915937201	H	9.901574010	2.250290977	–1.552002262
H	11.273822296	9.459701351	0.084356892	H	11.064125964	1.059035427	–0.890648855
C	8.696585268	10.482536758	–0.083247749	C	8.507997339	0.023539874	–0.599453374
H	7.844980178	10.936760648	–0.630022556	H	7.703455858	–0.474451827	–0.020397150
H	9.461798859	11.269450205	0.080480904	H	9.259688047	–0.743787690	–0.876789242
H	8.331428989	10.156960019	0.912175750	H	8.064403182	0.415016913	–1.537616134

[Fe(bpp^{H,CO2Et})$_2$]$^{2+}$
87
$FeC_{34}N_{10}H_{34}O_8$

High-spin				Low-spin			
Fe	5.225199000	4.733541000	0.029617000	Fe	5.105648594	4.768009519	–0.097886176
N	4.864047000	4.744629000	–2.072022000	N	4.859504007	4.841136701	–1.970191303
N	6.536831000	6.261570000	–0.805258000	N	6.440073078	6.126931460	–0.541574093
C	5.526675000	5.600587000	–2.870659000	C	5.606146277	5.689195725	–2.713792603
C	5.319427000	5.661984000	–4.254907000	C	5.458025969	5.786571929	–4.101234843
H	5.865908000	6.368483000	–4.892782000	H	6.067773201	6.478438696	–4.696723349
C	4.375510000	4.774690000	–4.799025000	C	4.490505314	4.959539729	–4.702570476
H	4.179299000	4.788345000	–5.880880000	H	4.342135956	5.007674167	–5.790998616
N	6.440675000	6.418473000	–2.166196000	N	6.494915600	6.408704468	–1.897811203
C	7.305552000	7.370047000	–2.621250000	C	7.435158392	7.359820041	–2.166634819
H	7.379558000	7.645334000	–3.678734000	H	7.631317418	7.720968923	–3.181797210
C	7.996999000	7.851262000	–1.508383000	C	8.012427032	7.708582701	–0.945766331
C	7.474877000	7.124433000	–0.401798000	C	7.355725864	6.910595058	0.036121001
H	7.744391000	7.203780000	0.657941000	H	7.519182611	6.884068586	1.119643195
C	3.678195000	3.870121000	–3.980471000	C	3.706946670	4.073792562	–3.938704272
C	3.964837000	3.900961000	–2.609766000	C	3.932268437	4.051592917	–2.558481480
N	3.353327000	3.060109000	–1.649792000	N	3.278085796	3.260915998	–1.598485036
N	3.747794000	3.178015000	–0.338956000	N	3.691034019	3.432731168	–0.285926342
N	5.578708000	4.853038000	2.127934000	N	5.351436247	4.704776031	1.774842702
C	6.511645000	4.072875000	2.702737000	C	6.250994752	3.846457319	2.307142074
C	6.795516000	4.115232000	4.073762000	C	6.475236402	3.767024324	3.685416087

H	7.560111000	3.472638000	4.528759000	H	7.208090225	3.067683824	4.108958320
C	6.056893000	5.024003000	4.850665000	C	5.721227575	4.624387100	4.508641844
C	5.076459000	5.844352000	4.267378000	C	4.782671663	5.522478293	3.966218229
C	4.876034000	5.713267000	2.886985000	C	4.632886925	5.524766197	2.575418228
N	3.928656000	6.458693000	2.146890000	N	3.769260347	6.327090221	1.811393990
N	3.842181000	6.239792000	0.793623000	N	3.815495041	6.135651598	0.439299408
N	6.768722000	3.262079000	0.465474000	N	6.476146618	3.377723657	–0.002353667
N	7.160467000	3.216301000	1.781799000	N	6.879498907	3.101644436	1.295065475
C	8.166542000	2.313370000	1.972672000	C	7.834916016	2.126646975	1.318892421
H	8.610376000	2.130909000	2.956659000	H	8.278042240	1.770980292	2.254879512
C	8.441729000	1.750029000	0.724775000	C	8.059666912	1.756031734	–0.007402525
C	7.538223000	2.378367000	–0.176486000	C	7.186839720	2.566141526	–0.789553940
H	7.425154000	2.209439000	–1.254487000	H	7.062600115	2.576918547	–1.879191490
C	2.868130000	7.042808000	0.353781000	C	2.925691046	6.985183454	–0.082877125
H	2.598183000	7.065954000	–0.708650000	H	2.762226507	7.036531358	–1.165310219
C	2.312331000	7.792663000	1.428375000	C	2.295185766	7.736544307	0.951314965
C	3.021756000	7.389890000	2.560844000	C	2.860343038	7.288221381	2.144940251
H	2.933302000	7.706437000	3.605512000	H	2.675902366	7.586361761	3.182513387
H	4.498162000	6.553336000	4.873621000	H	4.195476007	6.192339330	4.607733344
H	6.249145000	5.094117000	5.931046000	H	5.869660217	4.593155788	5.597666436
C	3.015682000	2.293689000	0.344905000	C	2.952492205	2.594051464	0.445267761
H	3.139327000	2.176224000	1.428680000	H	3.077305381	2.525030668	1.532563486
C	2.134436000	1.591925000	–0.523578000	C	2.051817347	1.870941393	–0.388655747
C	2.382845000	2.110851000	–1.795994000	C	2.289088767	2.323901916	–1.687102178
H	1.942360000	1.869516000	–2.768658000	H	1.832781209	2.049886676	–2.644004669
H	2.942409000	3.174797000	–4.404460000	H	2.951223968	3.430183596	–4.407939789
C	1.178761000	0.533082000	–0.113848000	C	1.080911597	0.853720008	0.085326112
O	1.047873000	0.153656000	1.042099000	O	0.971676576	0.529595077	1.260110688
O	0.490781000	0.056954000	–1.165979000	O	0.362161276	0.350329509	–0.935737683
C	–0.480088000	–0.997470000	–0.891779000	C	–0.647852242	–0.655609586	–0.616502684
H	0.057785000	–1.854764000	–0.436361000	H	–0.280046328	–1.265024729	0.232434733
H	–1.203102000	–0.618849000	–0.140292000	H	–0.697249089	–1.287034104	–1.524133342
C	9.438871000	0.712213000	0.363782000	C	8.994033607	0.738851667	–0.550025365
O	9.584618000	0.291625000	–0.775752000	O	9.096894589	0.488636528	–1.743461957
O	10.143877000	0.315646000	1.441018000	O	9.696547464	0.142665045	0.428323683
C	11.154211000	–0.720540000	1.242695000	C	10.647584245	–0.885347997	0.017755558
H	11.569081000	–0.614175000	0.221321000	H	10.087076219	–1.686885688	–0.506601012
H	11.940741000	–0.479707000	1.983026000	H	11.352765076	–0.436683808	–0.711990583
C	1.226706000	8.804555000	1.417258000	C	9.087939105	8.717015228	–0.773431652
O	0.838602000	9.396566000	2.415045000	O	9.577330679	9.351806529	–1.697526392
O	0.737229000	8.978525000	0.176881000	O	9.443507248	8.833125419	0.518148120
C	–0.336595000	9.954196000	0.027930000	C	10.496317091	9.795818421	0.822349457
H	–1.165769000	9.665255000	0.706162000	H	11.384054667	9.547709109	0.204602028
H	0.042472000	10.941391000	0.364889000	H	10.144965939	10.802317859	0.513583800
C	9.038413000	8.908050000	–1.541571000	C	1.252370558	8.787685805	0.847346994
O	9.399902000	9.475531000	–2.563270000	O	0.789184463	9.382524166	1.810649474
O	9.521945000	9.152105000	–0.310632000	O	0.892871652	8.990430101	–0.432445577
C	10.553623000	10.177350000	–0.203881000	C	–0.129668848	10.002599886	–0.672242606
H	11.394236000	9.896313000	–0.871315000	H	–1.019550243	9.750780800	–0.059160069
H	10.133139000	11.133017000	–0.580156000	H	0.258326573	10.978502584	–0.313215546
C	–1.150029000	–1.367748000	–2.199961000	C	10.786538337	9.718732979	2.307743340
H	–0.410823000	–1.738613000	–2.937933000	H	9.888105215	9.968325173	2.907097538
H	–1.890593000	–2.171979000	–2.016988000	H	11.135568112	8.707208375	2.596750394
H	–1.682977000	–0.499798000	–2.637789000	H	11.583606126	10.445271725	2.562852739
C	10.570994000	–2.106328000	1.470539000	C	11.352240368	–1.394260195	1.259306686
H	9.799541000	–2.348332000	0.712859000	H	10.632433499	–1.836006690	1.977442103
H	11.379694000	–2.861123000	1.390432000	H	12.079222473	–2.179962940	0.971575767
H	10.120638000	–2.189992000	2.479955000	H	11.906822224	–0.580121402	1.767962083
C	10.975340000	10.266406000	1.249229000	C	–1.989988722	–0.011666254	–0.305123743
H	10.121823000	10.545630000	1.898985000	H	–2.332914426	0.626921737	–1.144136265
H	11.392156000	9.303206000	1.606169000	H	–2.746456178	–0.806192229	–0.143593129
H	11.759090000	11.043048000	1.356425000	H	–1.937664277	0.603080085	0.615478929
C	–0.758821000	9.966196000	–1.427713000	C	–0.436252723	10.016268651	–2.156350038
H	0.082870000	10.255982000	–2.088193000	H	0.464489647	10.267852662	–2.751332392
H	–1.132777000	8.972308000	–1.745593000	H	–0.822240978	9.034113589	–2.495647742
H	–1.575390000	10.702829000	–1.566154000	H	–1.210622842	10.781936160	–2.362811781

$[Fe(bpp^{H,Cl})_2]^{2+}$
51
$FeC_{22}N_{10}H_{14}Cl_4$

High-spin				Low-spin			
Fe	5.162836000	5.162922000	–0.005888000	Fe	5.162859731	5.162976578	–0.000010709
N	5.163066000	5.163018000	–2.127148000	N	5.162971915	5.163039324	–1.889022522
N	6.653015000	6.653192000	–0.595212000	N	6.526746782	6.526786389	–0.322203504
C	5.987447000	5.987248000	–2.796758000	C	5.993533784	5.993511194	–2.560078929
C	6.029972000	6.029560000	–4.197374000	C	6.031648261	6.031565165	–3.958409815
H	6.704231000	6.703951000	–4.740196000	H	6.708056096	6.707977203	–4.497028262
C	5.163899000	5.162730000	–4.886039000	C	5.163336445	5.163059044	–4.646995345
H	5.164227000	5.162547000	–5.986235000	H	5.163448278	5.163009662	–5.746859390
N	6.788797000	6.788623000	–1.951716000	N	6.756766804	6.756671215	–1.663168774
C	7.731253000	7.730525000	–2.275306000	C	7.717659311	7.717313384	–1.849604803
H	7.986511000	7.985268000	–3.308727000	H	8.041349189	8.040981865	–2.843643941
C	8.218724000	8.219042000	–1.065273000	C	8.114131318	8.113773959	–0.575036774
C	7.516941000	7.516622000	–0.048071000	C	7.347129573	7.347030004	0.346833089
H	7.610635000	7.610406000	1.040573000	H	7.368546720	7.368607009	1.442550134
C	4.297387000	4.296066000	–4.197707000	C	4.294830194	4.294519650	–3.958661056
C	4.339077000	4.338665000	–2.797078000	C	4.332603218	4.332564094	–2.560306830
N	3.537210000	3.537463000	–1.952348000	N	3.569241045	3.569307267	–1.663539701
N	3.672332000	3.672964000	–0.595789000	N	3.798983274	3.799144090	–0.322503736
N	5.163167000	5.163083000	2.126723000	N	5.162984555	5.163053619	1.888996264
C	5.983395000	4.343043000	2.810402000	C	5.993609140	4.332710618	2.560121355
C	6.027593000	4.299541000	4.210175000	C	6.031840208	4.294861754	3.958455735
H	6.704457000	3.622402000	4.746702000	H	6.708280230	3.618523840	4.497126175
C	5.164390000	5.164073000	4.904899000	C	5.163584483	5.163481416	4.646977158
C	4.300565000	6.028099000	4.210317000	C	4.295052909	6.031940675	3.958575454
C	4.343519000	5.983600000	2.810532000	C	4.332713199	5.993681811	2.560218356
N	3.542737000	6.783574000	1.965008000	N	3.569349993	6.756860367	1.663406307
N	3.690019000	6.635466000	0.615402000	N	3.798988988	6.526811734	0.322391895
N	6.635444000	3.690101000	0.615169000	N	6.526732231	3.799128025	0.322274089
N	6.783455000	3.542480000	1.964740000	N	6.756822945	3.569448401	1.663263310
C	7.728039000	2.597886000	2.268511000	C	7.717735513	2.608842821	1.849817815
H	7.993073000	2.333329000	3.296839000	H	8.041441924	2.285316315	2.843898634
C	8.203536000	2.122039000	1.047179000	C	8.114241015	2.212251353	0.575298570
C	7.492955000	2.832224000	0.043737000	C	7.347103222	2.978762503	–0.346638448
H	7.575815000	2.748901000	–1.046520000	H	7.368518596	2.956912817	–1.442341548
C	2.831927000	7.492860000	0.044128000	C	2.978539712	7.347141400	–0.346464173
H	2.748438000	7.575750000	–1.046112000	H	2.956760098	7.368694064	–1.442166098
C	2.121606000	8.203129000	1.047704000	C	2.211741303	8.113939152	0.575520651
C	2.598015000	7.727984000	2.268950000	C	2.608653018	7.717674102	1.850015774
H	2.333458000	7.992828000	3.297328000	H	2.285273143	8.041472462	2.844114701
H	3.624206000	6.705619000	4.747001000	H	3.618849612	6.708482250	4.497297938
H	5.164860000	5.164559000	6.004808000	H	5.163770088	5.163690635	5.746840958
C	2.807955000	2.809711000	–0.049081000	C	2.978514037	2.978778739	0.346289126
H	2.713503000	2.715946000	1.039495000	H	2.956812248	2.957064658	1.441993954
C	2.106364000	2.107529000	–1.066569000	C	2.211670309	2.212103724	–0.575752476
C	2.594725000	2.595730000	–2.276366000	C	2.608472835	2.608568157	–1.850224487
H	2.339879000	2.341050000	–3.309906000	H	2.285028452	2.284911616	–2.844349302
H	3.623509000	3.621521000	–4.740812000	H	3.618571449	3.618076712	–4.497436943
Cl	0.900632000	0.902255000	–0.846536000	Cl	9.296927515	1.029902941	0.181094181
Cl	9.406807000	0.918661000	0.802806000	Cl	1.028981769	1.029706985	–0.181708435
Cl	0.917716000	9.405934000	0.803539000	Cl	9.296742005	9.296171309	–0.180696804
Cl	9.423661000	9.425060000	–0.844701000	Cl	1.029042138	9.296298360	0.181380784

$[Fe(bpp^{H,Br})_2]^{2+}$
51
$FeC_{22}N_{10}H_{14}Br_4$

High-spin				Low-spin			
Fe	5.162826000	5.162938000	–0.005889000	Fe	5.162843206	5.162942230	–0.000009309
N	5.163036000	5.163057000	–2.127272000	N	5.162954775	5.163009680	–1.889159694
N	6.652791000	6.652763000	–0.594695000	N	6.526602233	6.526589917	–0.321983631
C	5.987239000	5.987441000	–2.796877000	C	5.993552349	5.993397279	–2.560274602
C	6.029666000	6.029868000	–4.197429000	C	6.031674236	6.031456759	–3.958539980
H	6.703724000	6.704569000	–4.740129000	H	6.708137287	6.707823761	–4.497157853
C	5.163771000	5.162882000	–4.886160000	C	5.163315089	5.163044685	–4.647198150
H	5.164054000	5.162746000	–5.986349000	H	5.163427874	5.162998940	–5.747054568
N	6.788697000	6.788868000	–1.951474000	N	6.757038411	6.756704383	–1.663111653
C	7.731468000	7.731083000	–2.273009000	C	7.718248888	7.717406494	–1.847569818
H	7.984847000	7.984502000	–3.307428000	H	8.040298165	8.039045996	–2.843096922
C	8.219271000	8.219202000	–1.063371000	C	8.114769742	8.113963710	–0.573439385
C	7.516985000	7.516086000	–0.047632000	C	7.347140066	7.346892052	0.346894643
H	7.608211000	7.606708000	1.041680000	H	7.365411037	7.365420116	1.442951986
C	4.297489000	4.296000000	–4.197770000	C	4.294757993	4.294596824	–3.958798512
C	4.339176000	4.338607000	–2.797208000	C	4.332540256	4.332633848	–2.560509349
N	3.537285000	3.537258000	–1.952117000	N	3.568904605	3.569241243	–1.663496976
N	3.672590000	3.673364000	–0.595280000	N	3.799085372	3.799274702	–0.322295566
N	5.163153000	5.163122000	2.126938000	N	5.162963870	5.163021085	1.889135950
C	5.983323000	4.343041000	2.810599000	C	5.993632212	4.332775098	2.560325567
C	6.027531000	4.299550000	4.210311000	C	6.031865265	4.294927451	3.958594926
H	6.704350000	3.622367000	4.746850000	H	6.708365832	3.618644514	4.497270555
C	5.164395000	5.164166000	4.905039000	C	5.163555363	5.163451801	4.647181988
C	4.300626000	6.028246000	4.210431000	C	4.294982487	6.031824052	3.958708294
C	4.343569000	5.983704000	2.810708000	C	4.332656098	5.993565276	2.560416596
N	3.542700000	6.783787000	1.964828000	N	3.569063803	6.756916464	1.663351289
N	3.690326000	6.635174000	0.614986000	N	3.799118751	6.526633096	0.322175583
N	6.635095000	3.690416000	0.614788000	N	6.526605267	3.799269986	0.322067080
N	6.783446000	3.542323000	1.964600000	N	6.757135895	3.569395004	1.663222064
C	7.728294000	2.597359000	2.266304000	C	7.718419511	2.608782403	1.847809879
H	7.991626000	2.334379000	3.295672000	H	8.040508044	2.287320215	2.843382241
C	8.203747000	2.121630000	1.045331000	C	8.114996154	2.212101759	0.573733293
C	7.492622000	2.832521000	0.043387000	C	7.347192065	2.978895601	–0.346676167
H	7.572720000	2.752210000	–1.047504000	H	7.365459183	2.960094371	–1.442720608
C	2.832174000	7.492527000	0.043719000	C	2.978524732	7.347034317	–0.346523226
H	2.751442000	7.572369000	–1.047159000	H	2.959909988	7.365538514	–1.442567382
C	2.121275000	8.203496000	1.045777000	C	2.211172015	8.114236036	0.573923290
C	2.597667000	7.728529000	2.266676000	C	2.608091190	7.717836911	1.847982391
H	2.334797000	7.991813000	3.296085000	H	2.286368689	8.039624904	2.843568198
H	3.624319000	6.705831000	4.747112000	H	3.618726355	6.708323213	4.497430056
H	5.164873000	5.164676000	6.004942000	H	5.163737147	5.163661348	5.747038196
C	2.808102000	2.810063000	–0.048655000	C	2.978426540	2.978866254	0.346322863
H	2.716267000	2.719305000	1.040589000	H	2.959891791	2.960176501	1.442366864
C	2.106087000	2.107116000	–1.064688000	C	2.210975649	2.211850591	–0.574193189
C	2.594594000	2.595117000	–2.274091000	C	2.607772225	2.608486681	–1.848220401
H	2.341663000	2.341733000	–3.308629000	H	2.285943901	2.286900768	–2.843837941
H	3.623748000	3.621230000	–4.740783000	H	3.618438982	3.618208072	–4.497579027
Br	0.791288000	0.792793000	–0.826577000	Br	0.921520976	9.403367856	0.144059995
Br	9.515592000	0.809625000	0.779408000	Br	9.404417312	9.403046425	–0.143367187
Br	0.808670000	9.514782000	0.780047000	Br	0.921267663	0.922739516	–0.144454781
Br	9.533742000	9.533758000	–0.824649000	Br	9.404806504	0.923145431	0.143821652

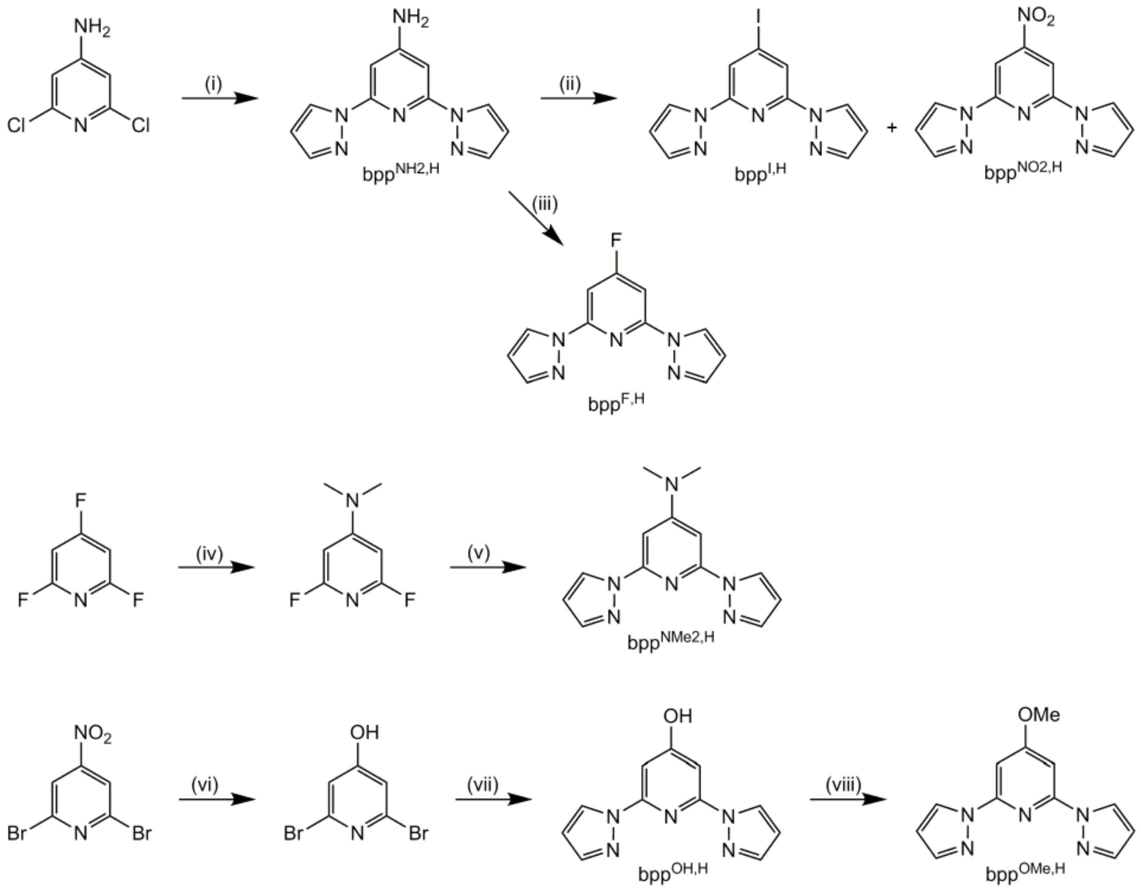

Scheme S1. Syntheses of the new bpp^{X,H} ligands in this work. Reagents and conditions: (i) Na[pz] (5 equiv), diglyme, 130 °C, N_2, 7 days, 94 %; (ii) isopentyl nitrite, I_2, KI, CH_2Cl_2, N_2, reflux, 5 h then $Na_2S_2O_{3(aq)}$, 52 % (bpp^{I,H})[22] and 1 % (bpp^{NO2}); (iii) $NaNO_2$, $HBF_{4(aq)}$, MeCN, N_2, reflux, 0.5 h then $NaOH_{(aq)}$, 20 %; (iv) Me_2NH, H_2O, rt, 1 h, 67 %;[23] (v) Na[pz] (3 equiv), diglyme, 130 °C, N_2, 36 h, 49 %. (vi) ^{n}Bu$_4$NOH$_{(aq)}$, THF, 48 h then $HCl_{(aq)}$, 70 %;[24] (vii) K[pz] (4 equiv), diglyme, 180 °C, N_2, 3-7 days, 11 %;[25] (viii) MeI, K_2CO_3, acetone, reflux, 24 h, 67 %.

Ligand synthesis

The following ligands were prepared according to the literature procedure: bpp^{Cl,H};[26] bpp^{Br,H};[27] bpp^{I,H};[22] bpp^{OH,H} [25] and bpp^{CO2H,H}.[28] The starting material 2,6-difluoro-4-dimethylaminopyridine was also prepared by the published method.[23] All other reagents were purchased commercially and used as supplied unless otherwise stated, although diglyme was always dried over sodium before use.

Synthesis of 4-amino-2,6-di(pyrazol-1-yl)pyridine (bpp^{NH2,H}). A new route was followed to this compound, which is simpler and higher yielding than the published synthesis.[22] A mixture of pyrazole (20.1 g, 0.30 mol) and potassium hydride (12.3 g, 0.30 mol) in diglyme (150 cm^3) was stirred vigorously under N_2 at 70 ^{o}C for 4 hours. 4-Amino-2,6-dichloropyridine (10.3 g, 63 mmol) was then added in one portion, and the mixture was stirred at 130 ^{o}C for 7 days. The solution was allowed to cool and quenched with cold water (500 cm^3) to yield a brown precipitate. This was filtered, washed with water and dried over P_2O_5. Yield 12.9 g (91 %). Found C, 58.7; H, 4.50; N, 37.1 %. Calcd for $C_{11}H_{10}N_6$ C, 58.4; H, 4.46; N, 37.1 %. ES mass spectrum: *m/z* 249.1 [Na(bpp^{NH2,H})]$^+$. ^{1}H NMR spectrum ({CD_3}$_2$SO): δ 6.56 (pseudo-t, 2.4 Hz, 2H, Pz H^4), 6.76 (s, 2H, NH_2), 7.10 (s, 2H, Py $H^{3/5}$), 7.81 (d, 2.4 Hz, 2H, Pz H^3), 8.86 (2, 2.4 Hz, 2H, Pz H^5). ^{13}C NMR spectrum ({CD_3}$_2$SO): δ 93.7 (2C, Py $C^{3/5}$), 107.8 (2C, Pz C^4), 127.9 (2C, Pz C^5), 142.0 (2C, Pz C^3), 150.8 (2C, Py $C^{2/6}$), 159.2 (1C, Py C^4).

Synthesis of 4-(dimethylamino)-2,6-di(pyrazol-1-yl)pyridine (bpp^{NMe2,H}). A suspension of pyrazole (6.1 g, 0.089 mol) and sodium hydride (60% dispersion in mineral oil, 3.6 g, 0.089 mol) in diglyme (80 cm^3) was stirred under N_2 at room temperature for 1 hr. 2,6-Difluoro-4-dimethylaminopyridine (4.7 g, 0.029 mol)[23] was then added, and the mixture was stirred at 130 ºC for 36 hours. Addition of water to the cooled reaction mixture afforded an off-white precipitate, which was washed with water and hexanes then dried *in vacuo*. Yield 3.7 g, 49 %. Found C, 57.2; H, 5.20; N, 31.5 %. Calcd for $C_{13}H_{14}N_6.H_2O$ C, 57.3; H, 5.92; N, 30.8 %. Mp 166−167 ºC. ES mass spectrum: *m/z* 277.1 [Na(bpp^{NMe2,H})]$^+$, 531.2 [Na(bpp^{NMe2,H})$_2$]$^+$. ^{1}H NMR spectrum ({CD_3}$_2$SO): δ 3.12 (s, 6H, N{CH_3}$_2$), 6.55 (pseudo-t, 1.8 Hz, 2H, Pz H^4), 7.05 (s, 2H, Py $H^{3/5}$), 7.79 (s, 2H, Pz H^3), 8.82 (d, 2.5 Hz, 2H, Pz H^5). ^{13}C NMR spectrum ({CD_3}$_2$SO): δ 30.7 (2C, N{CH_3}$_2$), 91.2 (2C, Py $C^{3/5}$), 107.7 (2C, Pz C^4), 127.9 (2C, Pz C^5), 141.9 (2C, Pz C^3), 150.5 (2C, Py $C^{2/6}$), 158.0 (1C, Py C^4).

Synthesis of 4-nitro-2,6-di(pyrazol-1-yl)pyridine (bpp^{NO2,H}). This compound was isolated from a synthesis of bpp^{I,H} from bpp^{NH2,H}, according to the literature procedure.[22] Purification of the crude product by silica gel column chromatography ($CHCl_3$ eluent) yielded bpp^{NO2} as a clearly visible red band (R_f 0.33). The isolated compound is a yellow solid. Yield 71 mg, 1 %. Found C, 51.8; H, 3.10; N, 32.9 %. Calcd for $C_{11}H_8N_6O_2$ C, 51.6; H, 3.15; N, 32.8 %. Mp 197−199 °C. ES mass spectrum: *m/z* 279.1 [Na(bpp^{NO2,H})]$^+$. ^{1}H NMR spectrum ($CDCl_3$): δ 6.57 (dd, 1.6 and 2.7 Hz, 2H, Pz H^4), 7.83 (d, 1.6 Hz, 2H, Pz H^4), 8.53 (s, 2H, Py $H^{3/5}$), 8.56 (d, 2.7, 2H, Pz H^5); ^{13}C NMR spectrum ($CDCl_3$): δ 103.8 (2C, Py $C^{3/5}$), 109.2 (2C, Pz C^4), 127.5 (2C, Pz C^5), 143.6 (2C, Pz C^3), 151.9 (2C, Py $C^{2/6}$), 158.0 (1C, Py C^4).

Synthesis of 4-fluoro-2,6-di(pyrazol-1-yl)pyridine (bpp^{F,H}). 48% Aqueous HBF_4 (10 cm^3, 54.7 mmol) was syringed into a Schlenk tube containing bpp^{NH2,H} (0.29 g, 1.28 mmol) under anaerobic conditions. The acidified contents were cooled to 0°C, and a degassed aqueous solution of $NaNO_2$ (0.20 g, 2.83 mmol) was gradually added with stirring causing the precipitation of a bright yellow solid. Addition of MeCN (15 cm^3) led to immediate evolution of N_2, and the mixture was then heated at 80°C for 0.5 h until the evolution ceased. Once cool, the MeCN was removed *in vacuo* and the remaining yellow solution poured into H_2O (30 cm^3) and neutralized with aqueous NaOH causing the formation of a pale yellow suspension. Extraction with $CHCl_3$ (3 x 50 cm^3), drying with $MgSO_4$, filtration and removal of the volatiles yielded a crude pale yellow solid which was purified through silica gel column chromatography (eluent: DCM; Rf value: 0.62). Colorless powder. Yield 58.0 mg, 20 %. Found C, 56.6; N, 3.75; N, 29.0 %. Calcd for $C_{11}H_8FN_5.½H_2O$ C, 55.5; H, 3.81; N, 29.4 %. Mp 106−108 °C. ES mass spectrum: *m/z* 230.1 [H(bpp^{F,H})]$^+$. ^{1}H NMR spectrum ($CDCl_3$): δ 6.52 (dd, 1.7 and 2.6, 2H, Pz H^4), 7.59 (d, 9.3 Hz, 2H, Py $H^{3/5}$), 7.77 (d, 1.7 Hz, 2H, Pz H^3), 8.55 (d, 2.6 Hz, 2H, Pz H^5). ^{13}C NMR spectrum ($CDCl_3$): δ 97.7 (d, 23.7 Hz, 2C, Py $C^{3/5}$), 108.4 (2C, Pz C^4), 127.3 (2C, Pz C^5), 142.8 (2C, Pz C^3), 152.1 (d, 13.4 Hz, 2C, Py $C^{2/6}$), 171.8 (d, 258 Hz, 1C, Py C^4). ^{19}F NMR spectrum ($CDCl_3$): δ −95.6 (t, 9.3 Hz).

Synthesis of 4-methoxy-2,6-di(pyrazol-1-yl)pyridine (bpp^{OMe,H}). MeI (0.19 g, 1.30 mmol) was added to a stirred suspension of K_2CO_3 (0.18 g, 1.30 mmol) and bpp^{OH,H} (0.18 g, 0.80 mmol)[25] in acetone (10 cm^3) under N_2, and the resultant mixture was refluxed for 24 h. The solution was concentrated to *ca.* 25 % its original volume, and then diluted to 50 cm^3 with $CHCl_3$. Aqueous NaOH (2 x 25 cm^3) was then used to wash the resultant suspension, and the volatiles were removed *in vacuo*. The resulting yellow solid was triturated in hexane (10 cm^3), collected on a glass frit and washed with a further few drops of hexane before drying *in vacuo*. Yield 0.13 g, 67 %. Found: C, 59.7; H, 4.65; N, 28.9 %. Calcd for $C_{12}H_{11}N_5O$ C, 59.7; H, 4.60; N, 29.0 %. Mp 106−108 °C. ES mass spectrum: *m/z* 264.1 [Na(bpp^{OMe,H})]$^+$. ^{1}H NMR spectrum ($CDCl_3$): δ 4.00 (s, 3H, OCH_3), 6.49 (dd, 1.7 and 2.4 Hz, 2H, Pz H^4), 7.41 (s, 2H, Py $H^{3/5}$), 7.75 (d, 1.7 Hz, 2H, Pz H^3), 8.56 (d, 2.4 Hz, 2H, Pz H^5); ^{13}C NMR (CD_3Cl) δ 56.1 (1C, OCH_3), 95.7 (2C, Py $C^{3/}$), 107.8 (2C, Pz C^4), 127.3 (2C, Pz C^5), 142.2 (2C, Pz C^3), 151.5 (2C, Py $C^{2/6}$), 170.0 (1C, Py C^4).

Synthesis of 2,6-di(4-{*tert*butyl}pyrazol-1-yl)pyridine (bpp^{H,tBu}). A solution of 4-*tert*butylpyrazole (0.50 g, 4.03 mmol) in *N*,*N*-dimethylformamide (70 cm^3) under N_2 was cooled to 0 °C in an ice bath. Solid NaH (60 wt % in mineral oil; 0.18 g, 4.40 mmol) was added slowly to the stirred solution. After 20 min 2,6-difluoropyridine (0.23 g, 4.03 mmol) was added, and the mixture was stirred at room temperature overnight. Water was added to quench the mixture which was then diluted to 200 cm^3 with additional water. This afforded a white precipitate which was collected, washed with water and dried *in vacuo*. Yield 0.39 g, 60 %. Found: C, 69.9; H, 8.00; N, 21.5 %. Calcd for $C_{19}H_{25}N_5$ C, 70.6; H, 7.79; N, 21.7 %. Mp 182−184 °C. ES mass spectrum: *m/z* 346.2 [Na(bpp^{H,tBu})]$^+$. ^{1}H NMR spectrum ({CD_3}$_2$SO): δ 1.33 (s, 18H, C{CH_3}$_3$), 7.74 (d, 8.1 Hz, 2H, Py $H^{3/5}$), 7.81 (s, 2H, Pz H^3), 8.06 (t, 8.1 Hz, 1H, Py H^4), 8.60 (s, 2H, Pz H^5); ^{13}C NMR ({CD_3}$_2$SO): δ 29.3 (2C, C{CH_3}$_3$), 31.3 (6C, C{CH_3}$_3$), 108.3 (2C, Py $C^{3/5}$), 123.3 (2C, Pz C^5), 135.1 (2C, Pz C^4), 140.6 (2C, Pz C^3), 142.1 (1C, Py C^4), 149.8 (2C, Py $C^{2/6}$).

Synthesis of the complexes

$[Fe(bpp^{Me,H})_2][BF_4]_2$,[29] $[Fe(bpp^{Br,H})_2][BF_4]_2$,[27] $[Fe(bpp^{I,H})_2][BF_4]_2$,[27] $[Fe(bpp^{OH,H})_2][BF_4]_2$,[25] $[Fe(bpp^{pz,H})_2][BF_4]_2$[30] and $[Fe(bpp^{H,I})_2][BF_4]_2$[19] were prepared by the literature methods. The method described below, for $[Fe(bpp^{NH2,H})_2][BF_4]_2$, was used to make the new complexes in this work. A solution of $bpp^{NH2,H}$ (0.20 g, 0.88 mmol) and $Fe[BF_4]_2 \cdot 6H_2O$ (0.15 g, 0.44 mmol) in nitromethane (15 cm^3) was refluxed until all the solid had dissolved (*ca.* 30 mins). The cooled solution was concentrated *in vacuo* to *ca.* 5 cm^3. Diffusion of diethyl ether vapor into the filtered solution afforded yellow crystals of the product which were collected by filtration, washed with excess diethyl ether and air-dried before analysis. The other complex salts were prepared using analogous reactions, with appropriate amounts of the relevant $bpp^{X,Y}$ ligand, and $Fe[ClO_4]_2 \cdot 6H_2O$ or $Fe[PF_6]_2$ (prepared *in situ* from $FeCl_2 \cdot 4H_2O$ and 2 equiv $AgPF_6$) as required.

$[Fe(bpp^{NH2,H})_2][BF_4]_2$. Yellow solid. Yield 0.26 g, 87 %. Found C, 38.4; H, 2.90; N, 24.2 %. Calcd for $C_{22}H_{20}B_2F_8FeN_{12}$ (681.94) C, 38.7; H, 2.96; N, 24.6 %.

$[Fe(bpp^{NH2,H})_2][ClO_4]_2$ Yellow solid. Yield 0.26 g, 84 %. Found C, 37.5; H, 2.80; N, 23.8 %. Calcd for $C_{22}H_{20}Cl_2FeN_{12}O_8$ (707.23) C, 37.4; H, 2.85; N, 23.8 %.

$[Fe(bpp^{NMe2,H})_2][BF_4]_2 \cdot ½H_2O$ Yellow solid. Yield 0.26 g, 78 %. Found C, 41.6; H, 3.80; N, 22.4 %. Calcd for $C_{26}H_{28}B_2F_8FeN_{12} \cdot ½H_2O$ (747.05) C, 41.8; H, 3.91; N, 22.5 %. The presence of a fractional equivalent of water in this material, and in the ClO_4^- salt listed below, was confirmed crystallographically.

$[Fe(bpp^{NMe2,H})_2][ClO_4]_2 \cdot ½H_2O$ Yellow solid. Yield 0.32 g, 95 %. Found C, 39.9; H, 3.70; N, 21.8 %. Calcd for $C_{26}H_{28}Cl_2FeN_{12}O_8 \cdot ½H_2O$ (772.34) C, 40.4; H, 3.78; N, 21.8 %.

$[Fe(bpp^{NO2,H})_2][BF_4]_2$ Dark red solid. Yield 0.30 g, 91 %. Found C, 35.2; H, 2.10; N, 22.1 %. Calcd for $C_{22}H_{16}B_2F_8FeN_{12}O_4$ (741.90) C, 35.6; H, 2.17; N, 22.6 %.

$[Fe(bpp^{OMe,H})_2][PF_6]_2$ Yellow solid. Yield 0.29 g, 80 %. Found C, 34.3; H, 2.60; N, 16.6 %. Calcd for $C_{24}H_{22}F_{12}FeN_{10}O_2P_2$ (828.28) C, 34.8; H, 2.67; N, 16.9 %.

$[Fe(bpp^{F,H})_2][BF_4]_2$ Yellow solid. Yield 0.24 g, 78 %. Found C, 38.6; H, 2.35; N, 20.1 %. Calcd for $C_{22}H_{16}B_2F_{10}FeN_{10}$ (687.89) C, 38.4; H, 2.34; N, 20.4 %.

$[Fe(bpp^{Cl,H})_2][BF_4]_2$ Dark brown solid. Yield 0.26 g, 83 %. Found C, 36.8; H, 2.25; N, 19.5 %. Calcd for $C_{22}H_{16}B_2Cl_2F_8FeN_{10}$ (720.80) C, 36.7; H, 2.23; N, 19.4 %.

$[Fe(bpp^{CO2H,H})_2][BF_4]_2 \cdot {}^3/_2H_2O$ Dark brown solid. Yield 0.24 g, 71 %. Found C, 37.8; H, 2.65; N, 17.9 %. Calcd for $C_{24}H_{18}B_2F_8FeN_{10}O_4 \cdot {}^3/_2H_2O$ (766.95) C, 37.6; H, 2.76; N, 18.3 %. The ClO_4^- salt of this complex has been previously published.[31]

$[Fe(bpp^{H,tBu})_2][BF_4]_2 \cdot 2H_2O$ Orange solid. Yield 0.19 g, 47 %. Found C, 49.9; H, 5.70; N, 15.3 %. Calcd for $C_{38}H_{50}B_2F_8FeN_{10} \cdot 2H_2O$ (912.37) C, 50.0; H, 5.96; N, 15.4 %.

[22] C. Rajadurai, F. Schramm, S. Brink, O. Fuhr, M. Ghafari, R. Kruk, M. Ruben, *Inorg. Chem.* **2006**, *45*, 10019.
[23] M. Schlosser, C. Bobbio, T. Rausis, *J. Org. Chem.* **2005**, *70*, 2494.
[24] A. Vidonne, D. Philp, *Tetrahedron*, **2008**, *64*, 8464.
[25] L. J. Kershaw Cook, M. A. Halcrow, *Magnetochemistry* **2015**, *1*, 3.
[26] L. Pukenas, F. Benn, E. Lovell, A. Santoro, L. J. Kershaw Cook, M. A. Halcrow, S. D. Evans, *J. Mater. Chem. C* **2015**, *3*, 7890.
[27] L. J. Kershaw Cook, H. J. Shepherd, T. P. Comyn, C. Baldé, O. Cespedes, G. Chastanet, M. A. Halcrow, *Chem. Eur. J.* **2015**, *21*, 4805.
[28] T. Vermonden, D. Branowska, A. T. M. Marcelis, E. J. R. Sudhölter, *Tetrahedron* **2003**, *59*, 5039.
[29] L. J. Kershaw Cook, F. L. Thorp-Greenwood, T. P. Comyn, O. Cespedes, G. Chastanet, M. A. Halcrow, *Inorg. Chem.* **2015**, *54*, 6319.
[30] L. J. Kershaw Cook, R. Mohammed, G. Sherborne, T. D. Roberts, S. Alvarez, M. A. Halcrow, *Coord. Chem. Rev.* **2015**, *289–290*, 2.
[31] A. Abhervé, M. Clemente-León, E. Coronado, C. J. Gómez-García, M. López-Jordà, *Dalton Trans.* **2014**, *43*, 9406.

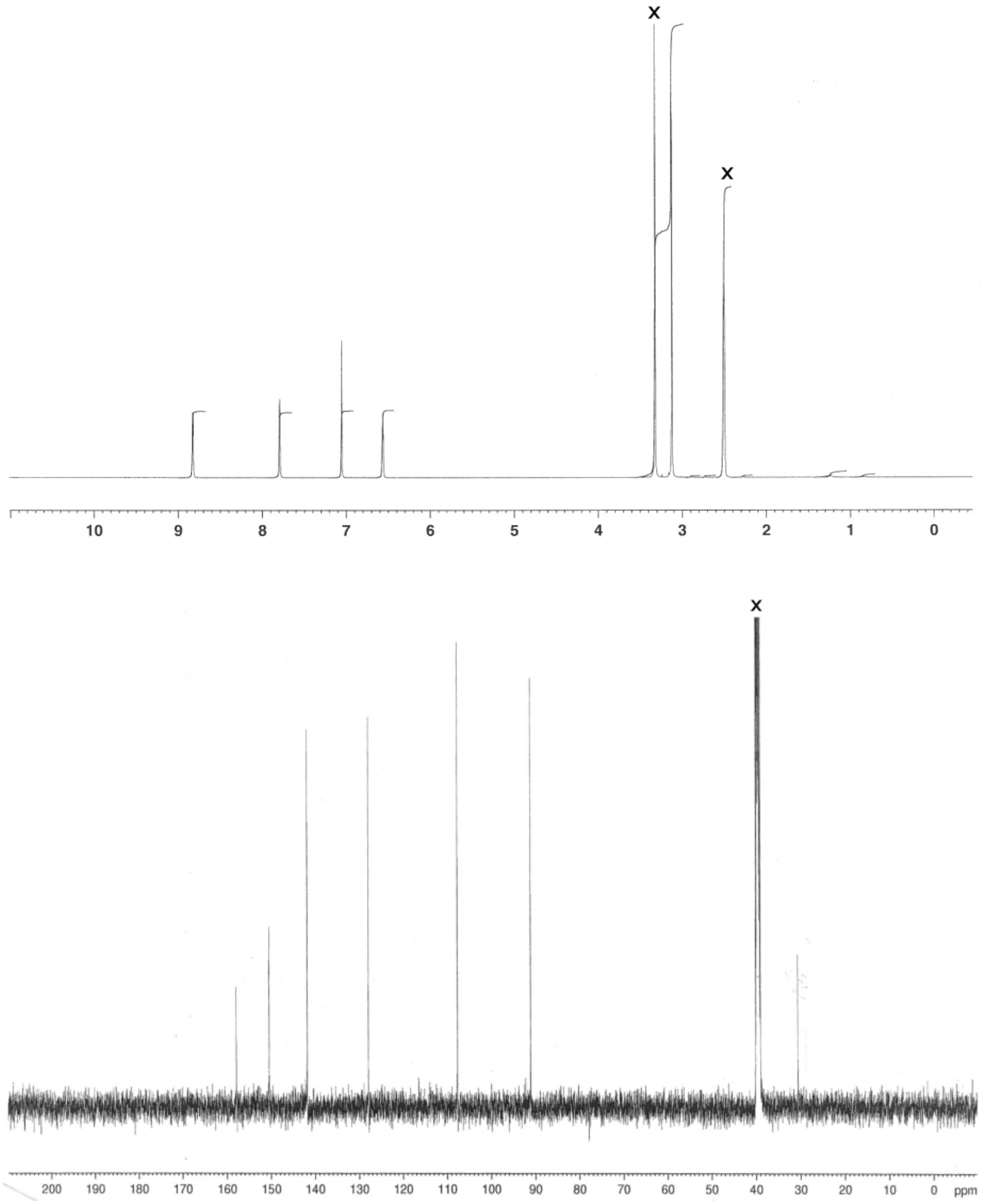

Figure S6 ^{1}H (top) and ^{13}C (bottom) NMR spectra of bpp^{NMe2,H} ($\{CD_3\}_2SO$).

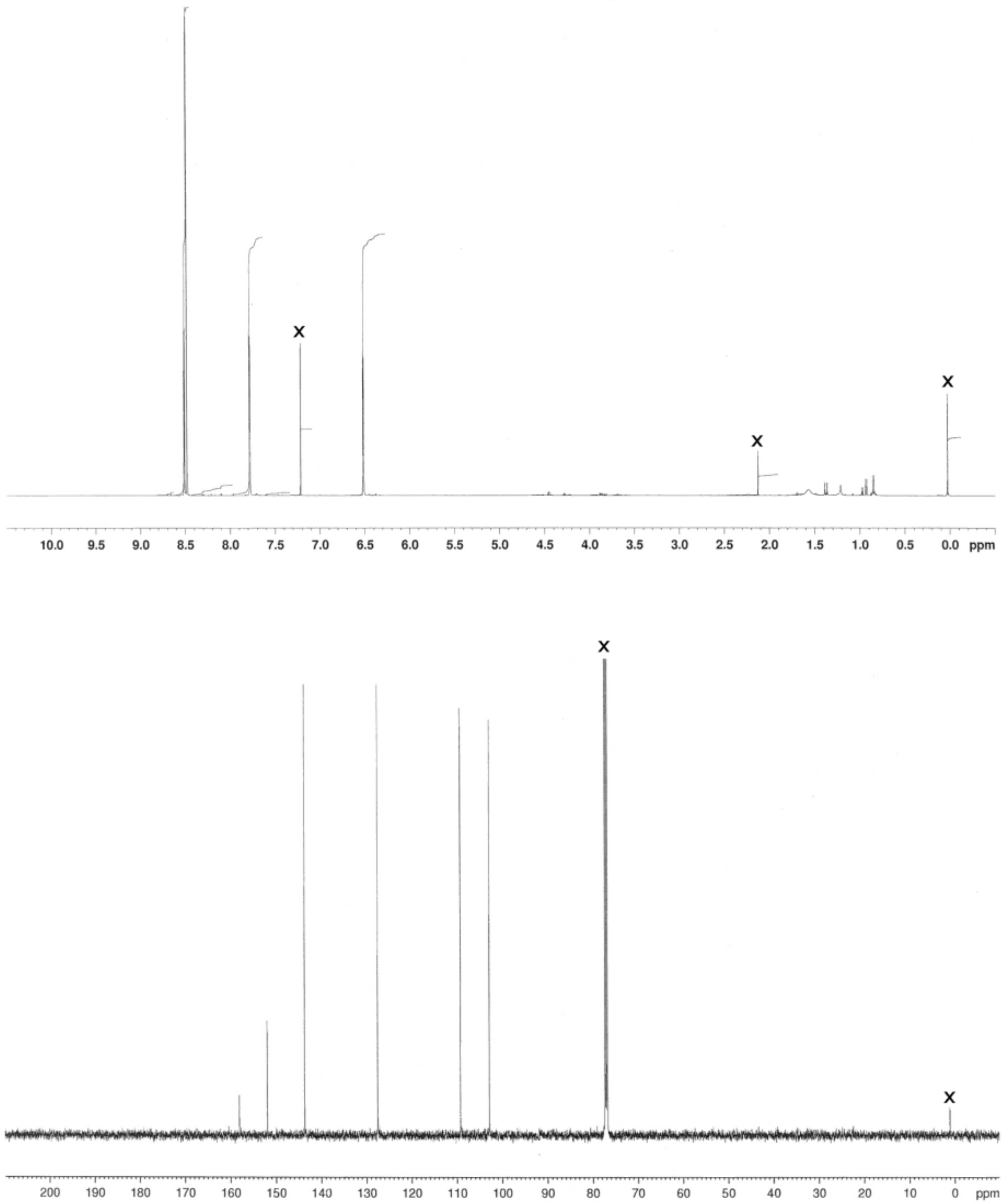

Figure S7 ^{1}H (top) and ^{13}C (bottom) NMR spectra of bpp^{NO2,H} ($CDCl_3$).

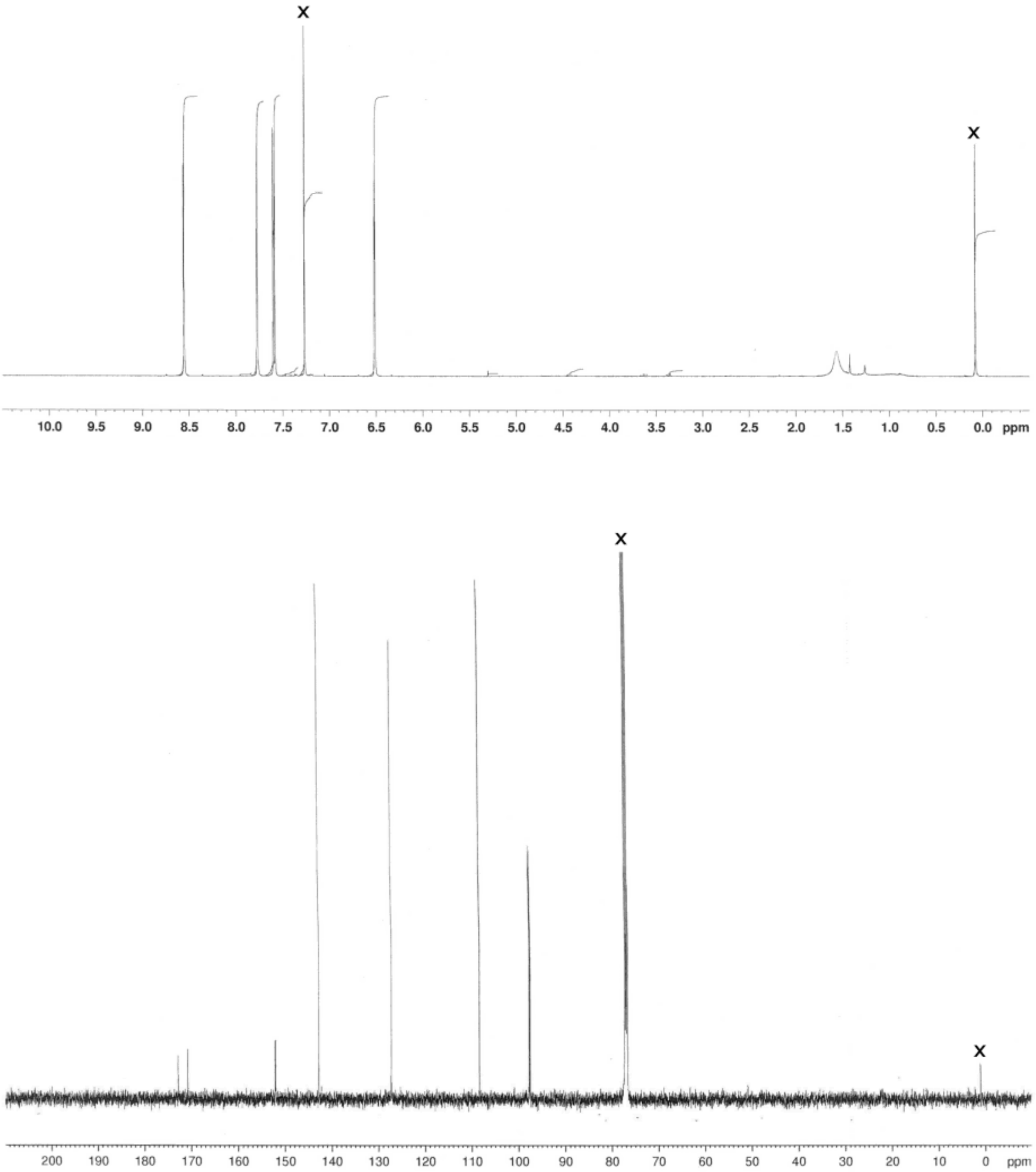

Figure S8 ^{1}H (top) and ^{13}C (bottom) NMR spectra of bpp^{F,H} ($CDCl_3$).

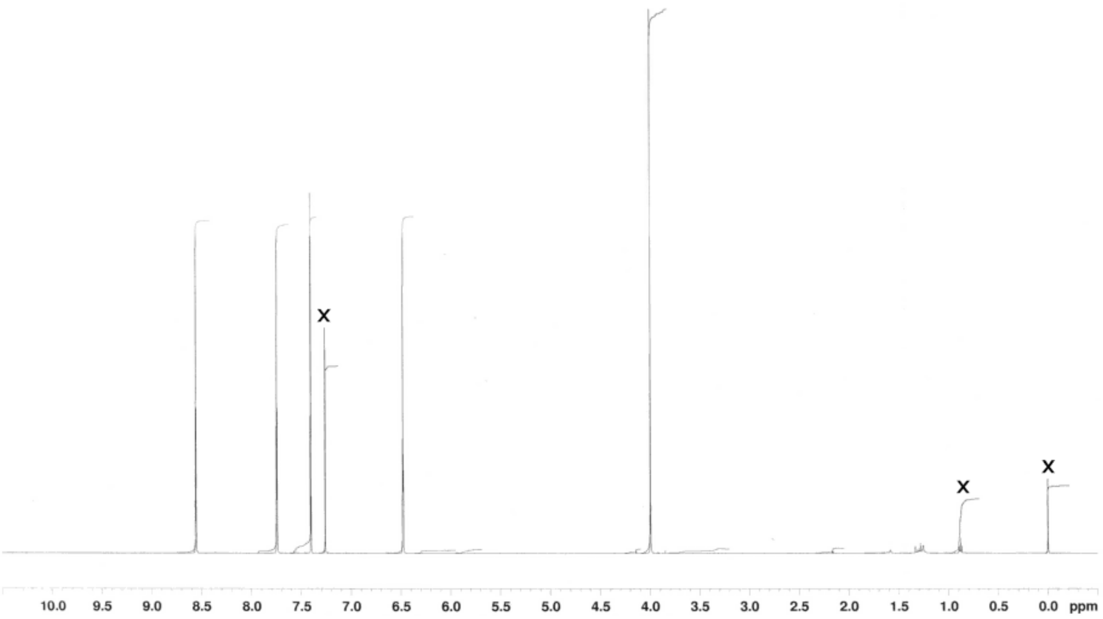

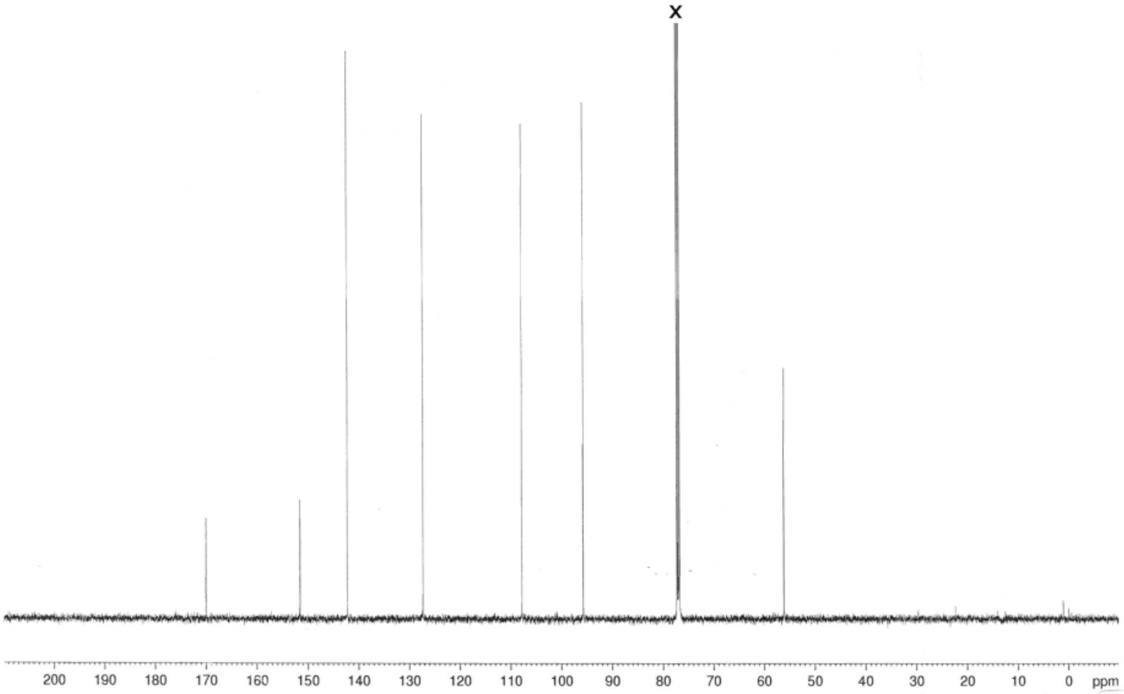

Figure S9 ^{1}H (top) and ^{13}C (bottom) NMR spectra of $bpp^{OMe,H}$ ($CDCl_3$).

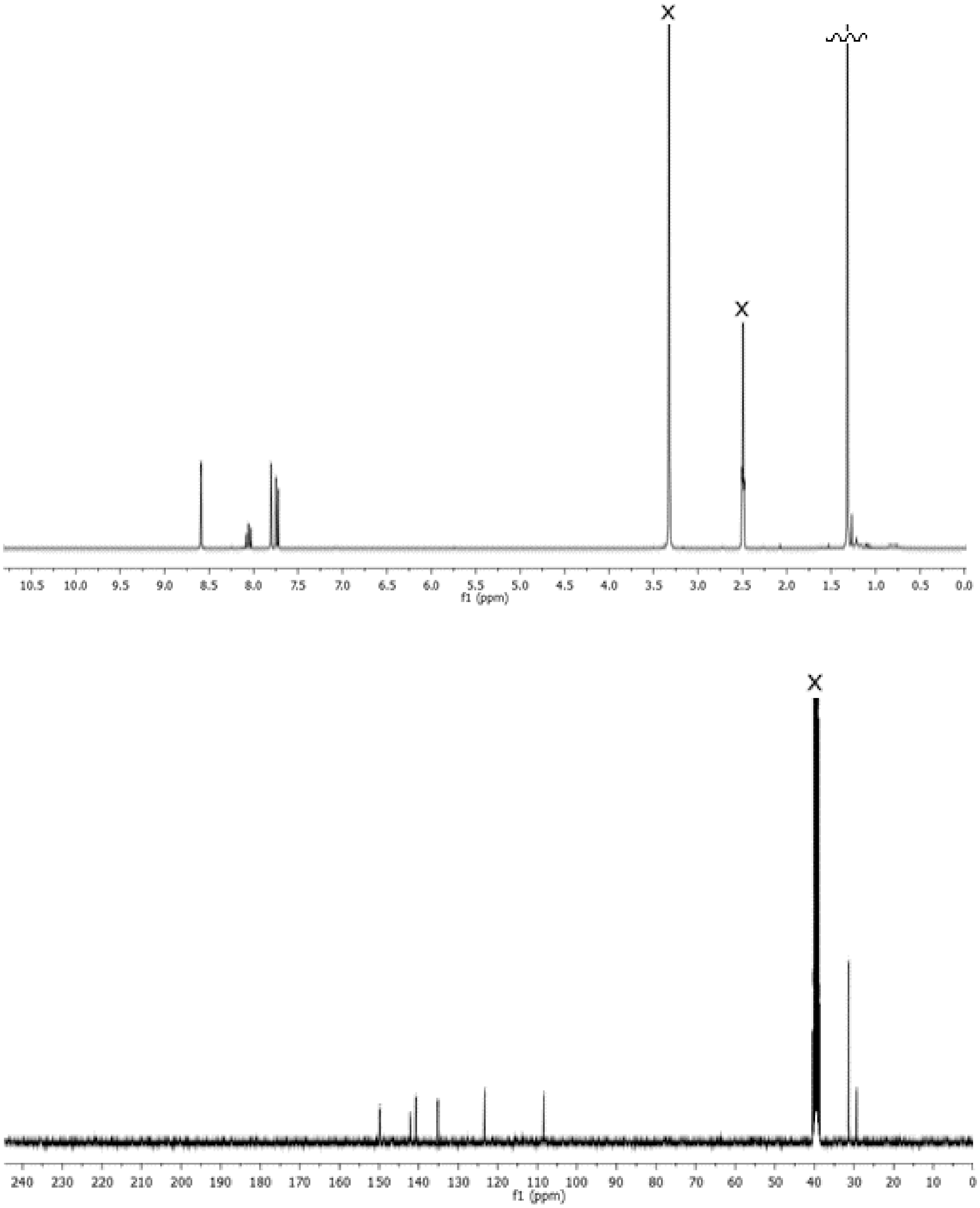

Figure S10 ^{1}H (top) and ^{13}C (bottom) NMR spectra of bpp^{H,tBu} ($\{CD_3\}_2SO$).

Single Crystal Structure Analyses

Single crystals of bpp^{NO2,H} were both obtained by crystallization from $CDCl_3$ inside an NMR tube, while crystalline bpp^{H,tBu} was obtained by... All the complex salts were crystallized by slow diffusion of diethyl ether into nitromethane solutions of the compounds, except for $[Fe(bpp^{OMe,H})_2][PF_6]_2$ where di-*iso*propyl ether was the antisolvent used. Diffraction data for the solvates of $[Fe(bpp^{NH2,H})_2]X_2$ were measured using a Bruker X8 Apex diffractometer, with graphite-monochromated Mo-K_α radiation (λ = 0.71073 Å) generated by a rotating anode. Other data were collected with an Agilent Supernova dual-source diffractometer using monochromated Mo-K_α radiation (λ = 0.71073 Å) or Cu-K_α radiation (λ = 1.54184 Å). Both diffractometers were fitted with Oxford Cryostream low-temperature devices. Experimental details of the structures determinations in this study are given in Table S1 and S2. All the structures were solved by direct methods (*SHELXS97*[32]), and developed by full least-squares refinement on F^2 (*SHELXL97*[32]). Crystallographic figures were prepared using *XSEED*.[33]

CCDC-1006313 (bpp^{NO2,H}), CCDC-1006314 ($[Fe(bpp^{NH2,H})_2][BF_4]_2\cdot 3MeNO_2$), CCDC-1006315 ($[Fe(bpp^{NH2,H})_2][ClO_4]_2\cdot 3MeNO_2$), CCDC-1006316 ($[Fe(bpp^{NMe2,H})_2][BF_4]_2\cdot xH_2O$), CCDC-1006317 ($[Fe(bpp^{NMe2,H})_2][ClO_4]_2\cdot xH_2O$), CCDC-1006321 ($[Fe(bpp^{OMe,H})_2][PF_6]_2$), CCDC-1006322 ($[Fe(bpp^{F,H})_2][BF_4]_2$, high-spin) CCDC-1006323 ($[Fe(bpp^{F,H})_2][BF_4]_2$, low-spin), CCDC-1006324 ($[Fe(bpp^{Cl,H})_2][BF_4]_2$) and CCDC-1422482 (bpp^{H,tBu}) contain the supplementary crystallographic data for this paper. These data can be obtained free of charge from The Cambridge Crystallographic Data Centre via www.ccdc.cam.ac.uk/data_request/cif.

[32] G. M. Sheldrick, *Acta Crystallogr. Sect. A* **2008**, *64*, 112.

[33] L. J. Barbour, *J. Supramol. Chem.* **2001**, *1*, 189.

Table S7 Experimental data for the ligand crystal structure determinations in this work.

	bpp[NO2,H]	bpp[H,*t*Bu]
molecular formula	$C_{11}H_8N_6O_2$	$C_{19}H_{25}N_5$
M_r	256.23	323.44
crystal colour	yellow plate	colorless prism
crystal class	monoclinic	triclinic
space group	$C2/c$	$P\bar{1}$
a [Å]	13.042(2)	10.7426(6)
b [Å]	9.6584(16)	12.0993(6)
c [Å]	9.7663(17)	15.6041(7)
α [°]	–	107.727(4)
β [°]	114.067(6)	90.826(4)
γ [°]	–	104.647(4)
V [Å^3]	1123.2(3)	1859.89(16)
Z	4	4
μ [mm^{-1}]	0.112[a]	0.557[b]
D_c [gcm^{-3}]	1.515	1.155
T [K]	150(2)	120(2)
measured reflections	2988	15679
independent reflections	1280	7040
observed reflections [$F_0 > 4\sigma(F_0)$]	957	6186
R_{int}	0.018	0.036
parameters	100	445
restraints	0	0
R_1 [$F_0 > 4\sigma(F_0)$][c]	0.058	0.053
wR_2, all data[d]	0.225	0.148
goodness of fit	1.171	1.028

[a]Collected with Mo-K_α radiation. [b] Collected with Cu-K_α radiation. [c]$R = \Sigma[|F_o| - |F_c|] / \Sigma|F_o|$ [d]$wR = [\Sigma w(F_o^2 - F_c^2) / \Sigma wF_o^4]^{1/2}$

Organic ligand structure refinements. The asymmetric unit of bpp[NO2,H] contains half a molecule, with N(1), C(4) and N(5) lying on the crystallographic C_2 axis 0, y, ¼. In contrast, bpp[H,*t*Bu] contains two unique molecules in its asymmetric unit (*ie* $Z' = 2$).

No disorder is present in any of these structures, and no restraints were applied to the refinements. All non-H atoms were refined anisotropically. For bpp[NO2,H], H atoms were located in the Fourier map and allowed to refine freely, with U_{iso} values constrained to 1.2x U_{eq} of the associated C atom. Attempts to refine H atom positions for bpp[H,*t*Bu] were unsuccessful, so H atoms in this structure were placed in calculated positions and refined using a riding model.

Table S8 Experimental data for the metal complex structure determinations in this work.

	$[Fe(bpp^{NH2,H})_2][BF_4]_2\cdot$3MeNO$_2$	$[Fe(bpp^{NH2,H})_2][ClO_4]_2\cdot$3MeNO$_2$	$[Fe(bpp^{NMe2,H})_2][BF_4]_2\cdot nH_2$O ($n \approx 0.29$)	$[Fe(bpp^{NMe2,H})_2][ClO_4]_2\cdot nH_2$O ($n \approx 0.22$)
molecular formula	$C_{25}H_{29}B_2F_8FeN_{15}O_6$	$C_{25}H_{29}Cl_2FeN_{15}O_{14}$	$C_{26}H_{28.58}B_2F_8FeN_{12}O_{0.29}$	$C_{26}H_{28.44}Cl_2FeN_{12}O_{8.22}$
M_r	865.10	890.38	743.30	767.32
crystal colour	yellow fragment	yellow prism	yellow prism	yellow prism
crystal class	monoclinic	monoclinic	orthorhombic	orthorhombic
space group	$P2_1/c$	$P2_1/c$	*Pbca*	*Pbca*
a [Å]	21.1810(6)	21.3665(8)	10.8941(4)	10.9387(4)
b [Å]	11.6538(3)	11.6494(5)	16.1236(8)	16.2484(9)
c [Å]	15.2026(4)	15.2693(5)	35.3237(14)	35.6396(16)
β [°]	100.1840(10)	99.4180(10)	–	–
V [Å^3]	3693.47(17)	3749.4(2)	6204.7(5)	6334.5(5)
Z	4	4	8	8
μ [mm^{-1}]	0.510[a]	0.629[a]	0.577[a]	0.715[a]
D_c [gcm^{-3}]	1.556	1.577	1.591	1.609
T [K]	150(2)	150(2)	100(2)	100(2)
measured reflections	60798	44388	30544	37621
independent reflections	9172	9343	7945	8244
observed reflections [$F_0 > 4\sigma(F_0)$]	6505	6974	5891	5903
R_{int}	0.077	0.069	0.061	0.079
parameters	565	565	472	471
restraints	12	12	20	20
R_1 [$F_0 > 4\sigma(F_0)$][c]	0.043	0.043	0.058	0.056
wR_2, all data[d]	0.126	0.117	0.121	0.106
goodness of fit	1.016	1.026	1.074	1.079
Flack parameter	–	–	–	–

[a]Collected with Mo-K_α radiation. [b] Collected with Cu-K_α radiation. [c]$R = \Sigma[|F_o| - |F_c|] / \Sigma|F_o|$ [d]$wR = [\Sigma w(F_o^2 - F_c^2) / \Sigma wF_o^4]^{1/2}$

Table S8 (continued)

	$[Fe(bpp^{OMe,H})_2][PF_6]_2$	$[Fe(bpp^{F,H})_2][BF_4]_2$<br>High-spin	 Low-spin	$[Fe(bpp^{Cl,H})_2][BF_4]_2$
molecular formula	$C_{24}H_{22}F_{12}FeN_{10}O_2P_2$	$C_{22}H_{16}B_2F_{10}FeN_{10}$	$C_{22}H_{16}B_2F_{10}FeN_{10}$	$C_{22}H_{16}B_2Cl_2F_8FeN_{10}$
M_r	828.31	687.92	687.92	720.82
crystal colour	yellow plate	yellow prism	dark brown prism	dark brown block
crystal class	monoclinic	monoclinic	monoclinic	orthorhombic
space group	$P2_1/c$	Cc	$P2_1$	$Pbcn$
a [Å]	19.0570(3)	12.2891(3)	8.4371(14)	17.7251(3)
b [Å]	9.7280(1)	11.8379(3)	8.5694(11)	9.2523(2)
c [Å]	16.9819(2)	19.0184(4)	18.354(3)	16.6822(3)
β [°]	106.266(2)	99.204(2)	97.249(19)	–
V [Å^3]	3022.20(7)	2731.12(11)	1316.4(3)	2735.85(9)
Z	4	4	2	4
μ [mm^{-1}]	6.121[b]	5.387[b]	5.588[b]	7.057[b]
D_c [gcm^{-3}]	1.820	1.673	1.735	1.750
T [K]	100(2)	290(2)	150(2)	100(2)
measured reflections	11462	5173	4996	11479
independent reflections	5937	3192	3418	2746
observed reflections [$F_0 > 4\sigma(F_0)$]	5536	3073	3212	2552
R_{int}	0.026	0.021	0.082	0.054
parameters	462	422	423	204
restraints	0	32	21	0
R_1 [$F_0 > 4\sigma(F_0)$][c]	0.034	0.039	0.101	0.039
wR_2, all data[d]	0.086	0.110	0.287	0.107
goodness of fit	1.014	1.060	1.229	1.038
Flack parameter	–	−0.004(4)	−0.004(10)	–

[a]Collected with Mo-K_α radiation. [b] Collected with Cu-K_α radiation. [c]$R = \Sigma[|F_o| - |F_c|] / \Sigma|F_o|$ [d]$wR = [\Sigma w(F_o^2 - F_c^2) / \Sigma wF_o^4]^{1/2}$

Structure refinements of [Fe(bpp^{NH2,H})$_2$][BF$_4$]$_2$·3MeNO$_2$ and [Fe(bpp^{NH2})$_2$][ClO$_4$]$_2$·3MeNO$_2$. These crystals are isostructural, and their diffraction data were modelled in the same way. One nitromethane molecule in both structures was disordered over two equally occupied sites, which were refined using the fixed restraints C–N = 1.48(2), N–O = 1.22(2), O...O = 2.14(2) and C...O = 2.32(2) Å. All non-H atoms were refined anisotropically, while C-bound H atoms were placed in calculated positions and refined using a riding model. The amino H atoms were located in the difference map and allowed to refine with a common U_{iso} displacement parameter.

Structure refinements of [Fe(bpp^{NMe2,H})$_2$][BF$_4$]$_2$·xH$_2$O and [Fe(bpp^{NMe2})$_2$][ClO$_4$]$_2$·xH$_2$O ($x \approx 0.25$). Data from this pair of isostructural crystals were also refined using the same procedure. One of the two anions in each structure is disordered over two sites, with refined occupancies close to 0.75:0.25. Refined distance and angle restraints were applied to the minor anion disorder site. In addition to the cations and anions, a Fourier peak that was not bonded to any other residue was modelled as a partial water molecule, whose occupancy also refined to approximately 0.25. This partial water site, O(50) lies within hydrogen bonding distance of the disordered anion, and of its symmetry equivalent related by ½+x, y, ½−z. Since the anion disorder appears to correlate with the presence or absence of O(50), O(50) was refined with the same occupancy as the minor disorder residue in the final least squares cycles. Although the partial H atoms associated with this water site could not be located or refined, the water H content is included in the density and F000 calculations. All non-H atoms with occupancy >0.5 were refined anisotropically, and H atoms were placed in calculated positions and refined using a riding model. The water content in the refinement is supported by microanalysis, since bulk samples of both salts analyse reasonably for a hemihydrate formulation.

Structure refinements of [Fe(bpp^{F,H})$_2$][BF$_4$]$_2$. Since cycling the crystal across the transition leads to rapid decay in diffraction quality, several attempts were required to obtain a good structure of the compound in its low-spin state. Hence, the two refinements reported here were obtained from different crystals, which have opposite handedness. One of the two unique anions was disordered at both temperatures, over three sites at 290 K and two sites at 150 K. This was modelled using refined B−F and F…F distance restraints. All non-H atoms except for the minor anion disorder sites were refined anisotropically, and H atoms were placed in calculated positions and refined using a riding model. There are ten residual peaks of +1.0–1.5 e.Å^{-3} in the low temperature refinement all of them <1 Å from Fe1 or another non-H atom in the complex dication.

Structure refinements of [Fe(bpp^{Cl,H})$_2$][BF$_4$]$_2$ and [Fe(bpp^{OMe,H})$_2$][PF$_6$]$_2$. The asymmetric unit of [Fe(bpp^{Cl})$_2$][BF$_4$]$_2$ contains half a formula unit with Fe(1) spanning the crystallographic C_2 axis at 0, y, ¾. In contrast, the complex molecule in [Fe(bpp^{OMe})$_2$][PF$_6$]$_2$ lies on a general crystallographic site with no internal symmetry. No disorder was detected during refinement of any of these structures, and no restraints were applied. All non-H atoms were refined anisotropically, while H atoms were placed in calculated positions and refined using a riding model.

Other measurements

Elemental microanalyses were performed by the University of Leeds School of Chemistry microanalytical service. Infra-red spectra were obtained as nujol mulls pressed between NaCl windows, between 600-4,000 cm^{-1}, using a Nicolet Avatar 360 spectrophotometer. ^{1}H NMR spectra employed a Bruker DPX300 spectrometer operating at 300.2 MHz. Electrospray mass spectra (ESI MS) were obtained on a Waters ZQ4000 spectrometer, from MeCN feed solutions. All mass peaks have the correct isotopic distributions for the proposed assignments.

Solid state magnetic susceptibility measurements were performed on a Quantum Design SQUID or SQUID/VSM magnetometer, with an applied field of 1000 or 5000 G and a scan rate of 5 Kmin^{-1}. A diamagnetic correction for the sample was estimated from Pascal's constants;[2] a diamagnetic correction for the sample holder was also applied.

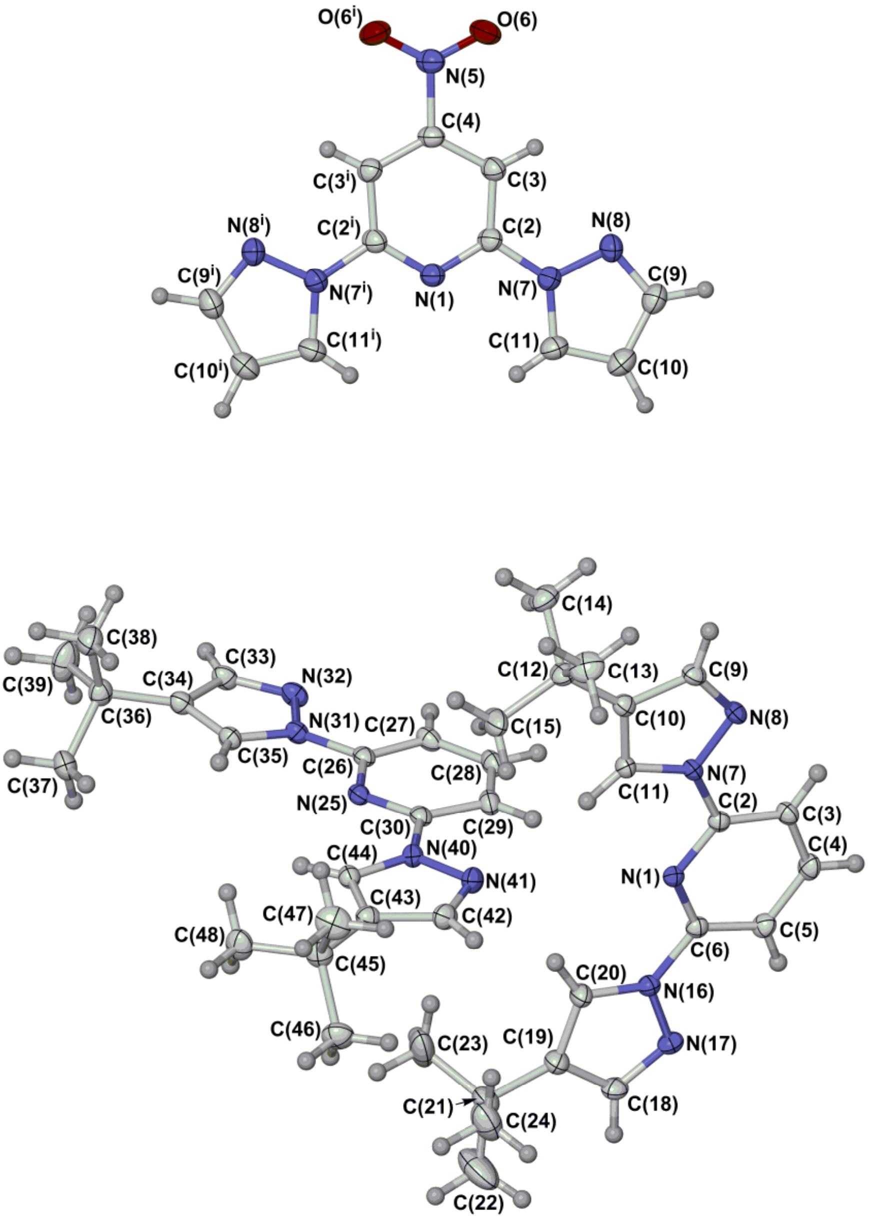

Figure S11 Views of the molecules in the ligand crystal structures: bpp^{NO2,H} (top) and bpp^{H,tBu} (both molecules, bottom). Displacement ellipsoids are at the 50 % probability level, except for H atoms which have arbitrary radii. Symmetry code: (i) −*x*, *y*, ½−*z*.

Color code: C, white; H, pale grey; N, blue; O, red.

Neighboring heterocyclic groups in each ligand structure have a *transoid*-coplanar orientation, which is normal for compounds of this type.[34]

[34] C. A. Bessel, R. F. See, D. L. Jameson, M. R. Churchill, K. J. Takeuchi, *J. Chem. Soc. Dalton Trans.* **1992**, 3223.

Table S9 Spin-state behavior of the new complexes in this work in the solid state (HS = high-spin, LS = low-spin, SCO = spin-crossover). Crystallographic and magnetic susceptibility data from these compounds are in the following Figures and Tables.

	Single crystal		Powder		
	T / K	Spin state	Spin state	$T_{½}$↓ / K	$T_{½}$↑ / K
$[Fe(bpp^{NH2,H})_2][BF_4]_2$	150	HS[a]	HS	–	–
$[Fe(bpp^{NH2,X})_2][ClO_4]_2$	150	HS[a]	HS	–	–
$[Fe(bpp^{NMe2,H})_2][BF_4]_2 \cdot xH_2O$	100	HS	HS	–	–
$[Fe(bpp^{NMe2,H})_2][ClO_4]_2 \cdot xH_2O$	100	HS	HS	–	–
$[Fe(bpp^{NO2,H})_2][BF_4]_2$[b]	–	–	SCO	287	287
$[Fe(bpp^{OMe,H})_2][PF_6]_2$	100	HS	HS	–	–
$[Fe(bpp^{F,H})_2][BF_4]_2$[b]	290/150	HS/LS	SCO	232	241
$[Fe(bpp^{Cl,H})_2][BF_4]_2$	100	LS	LS	–	–
$[Fe(bpp^{CO2H,H})_2][BF_4]_2 \cdot H_2O$[b,c]	–	–	LS	–	–
$[Fe(bpp^{H,tBu})_2][BF_4]_2 \cdot 2H_2O$[b]	–	–	HS/SCO[d]	240	240

[a]Solvated crystal structure, whose properties could differ from the dried bulk material. [b]No crystal structure of this compound was obtained. [c]The ClO_4^- salt of this complex has been previously published.[31] [d]The sample is poorly crystalline, and contains a mixture of HS and SCO-active material. The spin-transition is well-defined, but only proceeds to *ca.* 55 % completeness on cooling. The remainder of the material remains high-spin below 100 K.

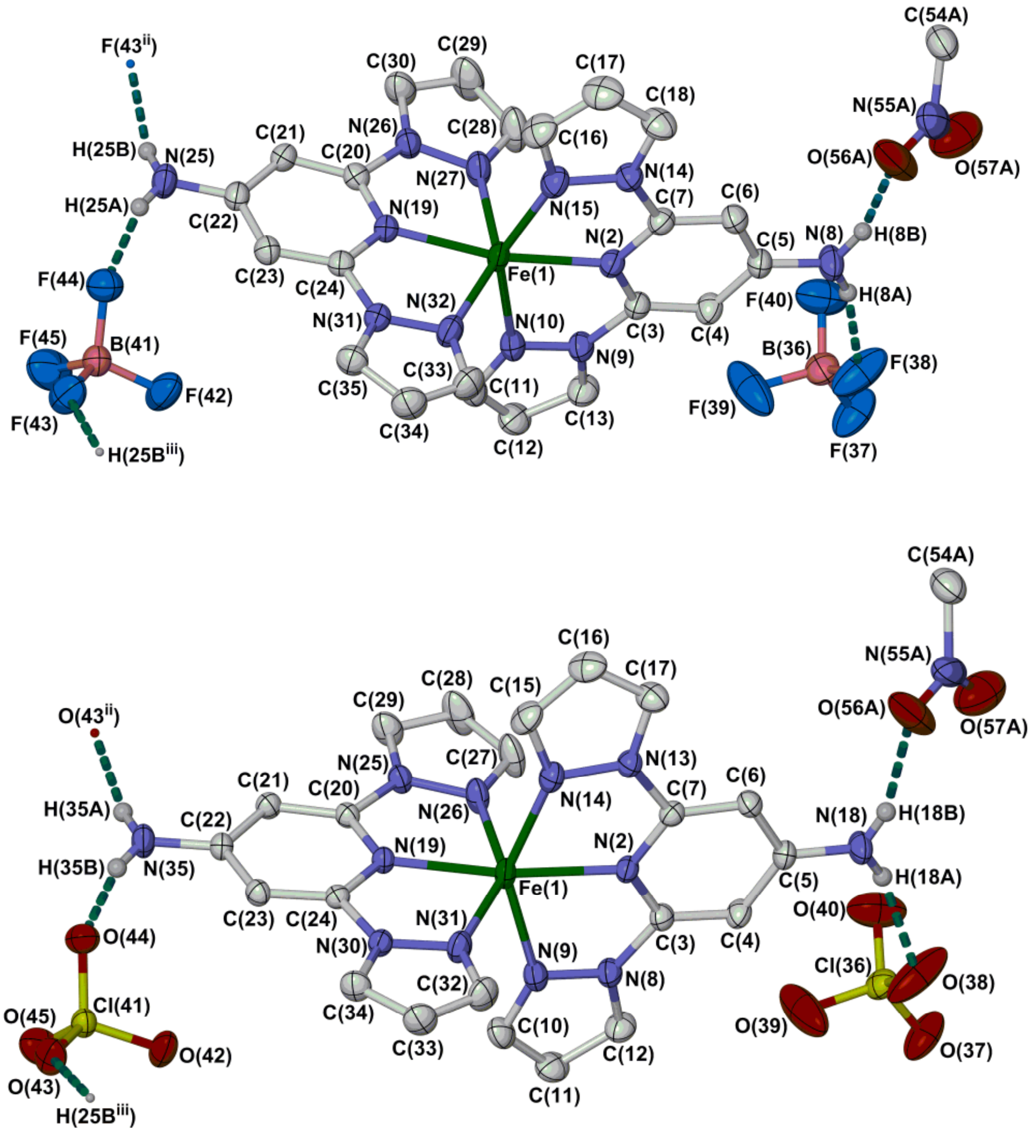

Fig. S12. View of the hydrogen bonded moieties in $[Fe(bpp^{NH2,H})_2[BF_4]_2\cdot3MeNO_2$ (top) and $[Fe(bpp^{NH2,H})_2[ClO_4]_2\cdot3MeNO_2$ (bottom), showing the full atom numbering scheme.

Atomic displacement ellipsoids are at the 50 % probability level. Only one orientation of the disordered nitromethane molecule is shown. Solvent molecules that do not take part in hydrogen bonding, and C-bound H atoms, have been omitted. Symmetry codes: (ii) *x*, ½–*y*, ½+*z*;
(iii) *x*, ½–*y*, –½+*z*.

Color code: C, white; H, grey; Cl, yellow; Fe, green; N, blue; O, red.

Table S10 Selected bond lengths and angles in the crystal structures of $[Fe(bpp^{NH2,H})_2][BF_4]_2\cdot3MeNO_2$ and $[Fe(bpp^{NH2,H})_2][ClO_4]_2\cdot3MeNO_2$ (Å, °). See Fig. S12 for the atom numbering scheme employed.

	$X^- = BF_4^-$	$X^- = ClO_4^-$
Fe(1)–N(2)	2.1244(17)	2.1250(17)
Fe(1)–N(9)	2.219(2)	2.219(2)
Fe(1)–N(14)	2.200(2)	2.200(2)
Fe(1)–N(19)	2.1134(17)	2.1113(16)
Fe(1)–N(26)	2.208(2)	2.2090(19)
Fe(1)–N(31)	2.193(2)	2.1924(19)
N(2)–Fe(1)–N(9)	72.85(7)	72.74(7)
N(2)–Fe(1)–N(14)	73.04(7)	73.03(7)
N(2)–Fe(1)–N(19) (ϕ)	166.47(7)	166.80(7)
N(2)–Fe(1)–N(26)	93.94(7)	93.99(7)
N(2)–Fe(1)–N(31)	119.67(7)	119.57(7)
N(9)–Fe(1)–N(14)	145.29(7)	145.22(6)
N(9)–Fe(1)–N(19)	109.28(7)	108.07(7)
N(9)–Fe(1)–N(26)	95.95(8)	95.52(8)
N(9)–Fe(1)–N(31)	92.91(7)	92.52(7)
N(14)–Fe(1)–N(19)	105.39(7)	106.66(7)
N(14)–Fe(1)–N(26)	92.60(8)	92.80(7)
N(14)–Fe(1)–N(31)	98.34(7)	98.98(7)
N(19)–Fe(1)–N(26)	72.62(7)	72.81(6)
N(19)–Fe(1)–N(31)	73.81(7)	73.62(6)
N(26)–Fe(1)–N(31)	146.36(7)	146.35(7)
θ	89.33(2)	88.57(2)

Table S11 Hydrogen bond parameters for the crystal structures of $[Fe(bpp^{NH2,H})_2]^{2+}$ complex salts (Å, °).[a]

	D–H	H…A	D…A	D–H…A
$[Fe(bpp^{NH2})_2][BF_4]_2\cdot3MeNO_2$				
N(18)–H(18A)…F(38)	0.90(3)	2.01(3)	2.891(3)	165(3)
N(18)–H(18B)…O(57A)/O(57B)	0.91(3)	2.47(3)/2.19(3)	3.340(14)/3.096(12)	161(2)/176(2)
N(35)–H(25A)…F(44)	0.82(3)	2.27(3)	3.060(3)	162(3)
N(35)–H(25B)…$F(43^{ii})$	0.86(3)	2.23(3)	3.071(3)	166(3)
$[Fe(bpp^{NH2})_2][ClO_4]_2\cdot3MeNO_2$				
N(18)–H(18A)…O(38)	0.86(3)	2.12(3)	2.944(3)	162(2)
N(18)–H(18B)…O(57A)/O(57B)	0.80(3)	2.60(3)/2.28(3)	3.371(10)/3.085(9)	162(3)/179(3)
N(35)–H(35A)…O(44)	0.81(3)	2.33(3)	3.092(3)	158(3)
N(35)–H(35B)…$O(43^{ii})$	0.88(3)	2.27(3)	3.138(3)	169(2)

[a]Symmetry code: (ii) x, $^1/_2-y$, $^1/_2+z$

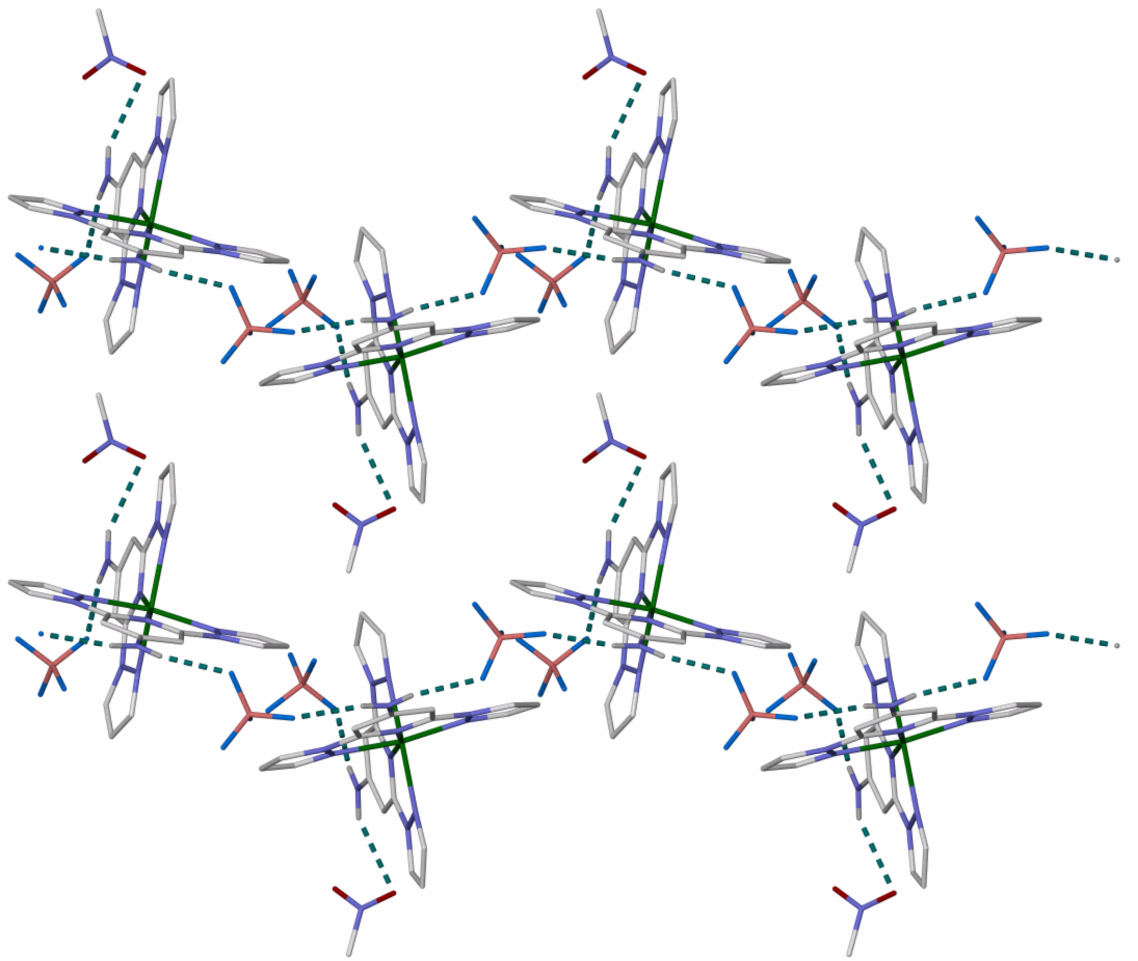

Fig. S13 Packing diagram of [Fe(bpp^{NH2,H})$_2$][BF_4]$_2$·3$MeNO_2$, showing the association of the complex cations into puckered hydrogen-bonded chains *via* the bridging BF_4^- ion B(41)-F(45).

The view is parallel to the (100) plane, with the unit cell *c*-axis horizontal. Only one orientation of the disordered nitromethane molecule is shown. Solvent molecules that do not take part in hydrogen bonding, and C-bound H atoms, have been omitted.

Color code: C, white; H, grey; B, pink; F, cyan; Fe, green; N, blue; O, red.

The hydrogen bonding connectivity in [Fe(bppNH$_2$)$_2$][ClO_4]$_2$·3$MeNO_2$ is visually indistinguishable from this figure.

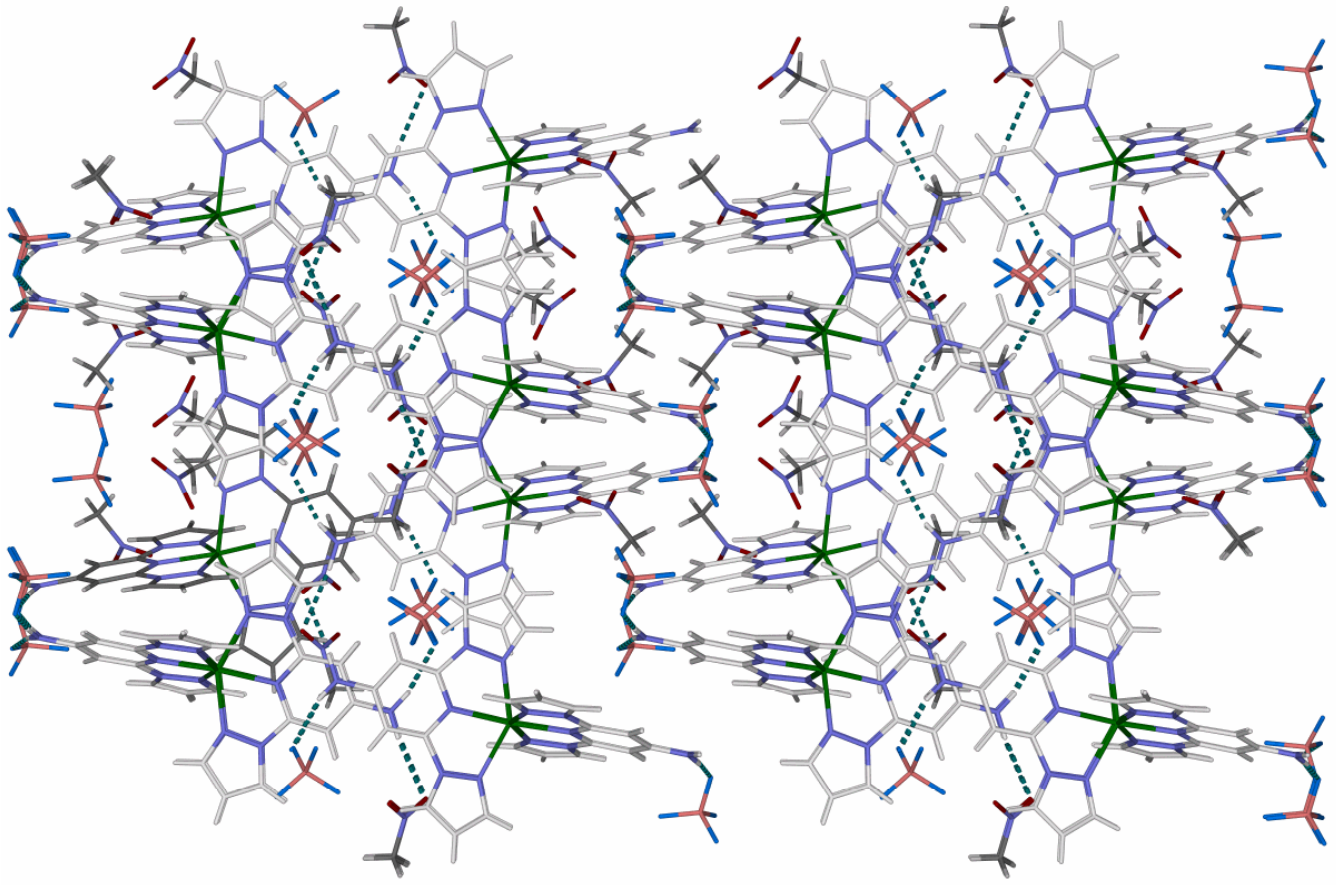

Fig. S14 Packing diagram of [Fe(bpp^{NH2,H})$_2$][BF_4]$_2$·3$MeNO_2$. The view is perpendicular to the [001] vector. Only one orientation of the disordered nitromethane molecule is shown.

Color code: C{complex}, white; C{solvent}, dark grey; H, pale grey; B, pink; F, cyan; Fe, green; N, blue; O, red.

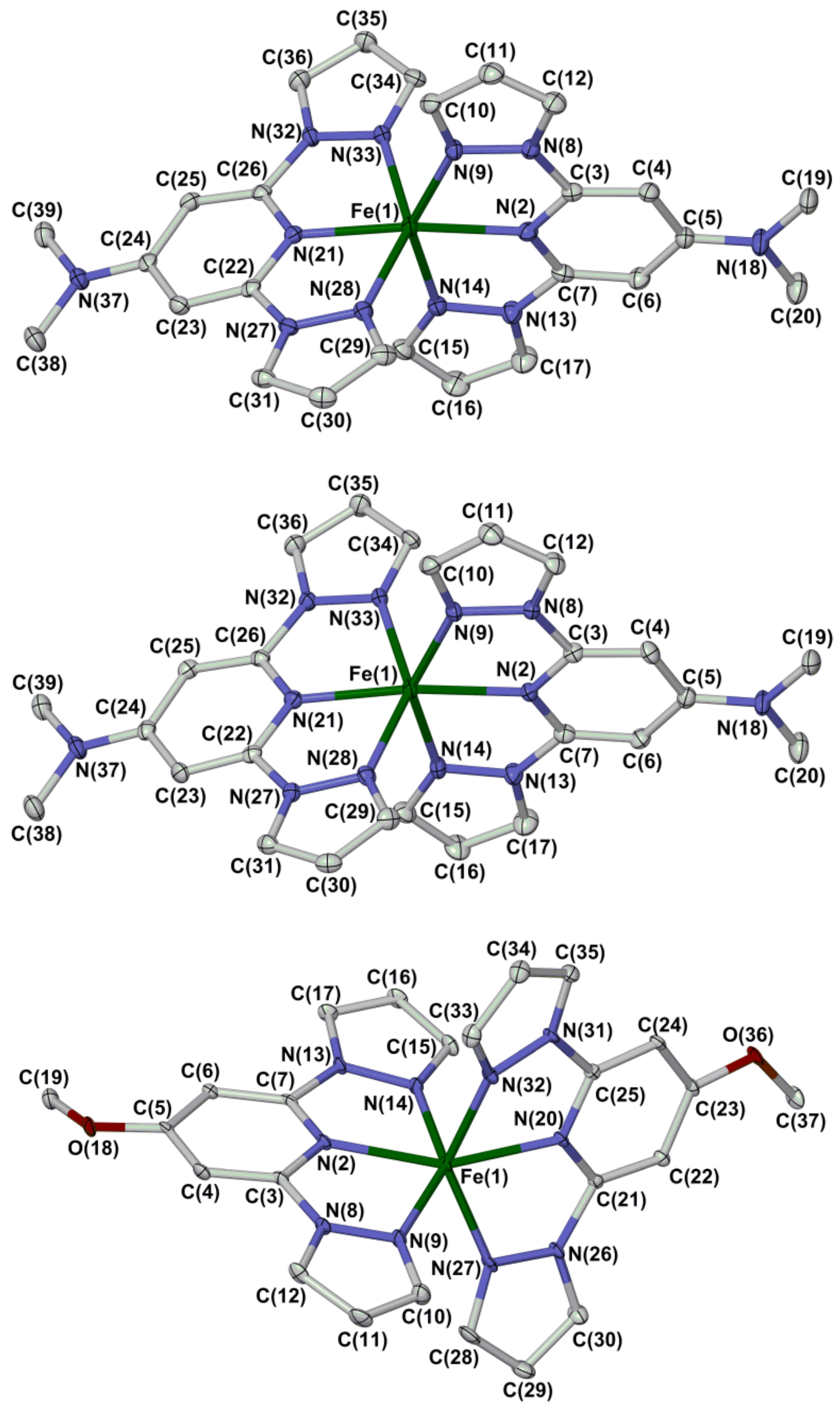

Fig. S15. View of the complex dications in $[Fe(bpp^{NMe2,H})_2][BF_4]_2 \cdot xH_2O$ (top), $[Fe(bpp^{NMe2,H})_2][ClO_4]_2 \cdot xH_2O$ (centre) and $[Fe(bpp^{OMe,H})_2][PF_6]_2$ (bottom), showing the full atom numbering scheme. Atomic displacement ellipsoids are at the 50 % probability level and H atoms have been omitted for clarity.

Color code: C, white; Fe, green; N, blue; O, red.

Table S12 Selected bond lengths and angles in the crystal structures of $[Fe(bpp^{NMe2,H})_2][BF_4]_2 \cdot xH_2O$, $[Fe(bpp^{NMe2,H})_2][ClO_4]_2 \cdot xH_2O$ and $[Fe(bpp^{OMe,H})_2][PF_6]_2$ (Å, °). See Fig. S15 for the atom numbering schemes employed.

	$[Fe(bpp^{NMe2})_2]X_2$		$[Fe(bpp^{OMe})_2][PF_6]_2$	
	X^- = BF_4^-	X^- = ClO_4^-		
Fe(1)–N(2)	2.112(2)	2.119(2)	Fe(1)–N(2)	2.1551(15)
Fe(1)–N(9)	2.201(2)	2.201(2)	Fe(1)–N(9)	2.2227(16)
Fe(1)–N(14)	2.198(2)	2.191(2)	Fe(1)–N(14)	2.1679(16)
Fe(1)–N(21)	2.113(2)	2.118(2)	Fe(1)–N(20)	2.1452(15)
Fe(1)–N(28)	2.200(2)	2.203(2)	Fe(1)–N(27)	2.1745(16)
Fe(1)–N(33)	2.168(2)	2.171(2)	Fe(1)–N(32)	2.1919(16)
N(2)–Fe(1)–N(9)	73.16(8)	73.22(8)	N(2)–Fe(1)–N(9)	71.81(6)
N(2)–Fe(1)–N(14)	72.87(8)	72.97(8)	N(2)–Fe(1)–N(14)	73.03(6)
N(2)–Fe(1)–N(21) (ϕ)	164.38(8)	164.33(8)	N(2)–Fe(1)–N(20) (ϕ)	153.61(6)
N(2)–Fe(1)–N(28)	112.52(8)	111.98(8)	N(2)–Fe(1)–N(27)	120.58(6)
N(2)–Fe(1)–N(33)	102.84(8)	103.19(8)	N(2)–Fe(1)–N(32)	101.05(6)
N(9)–Fe(1)–N(14)	146.03(8)	146.19(8)	N(9)–Fe(1)–N(14)	144.30(6)
N(9)–Fe(1)–N(21)	121.66(8)	121.77(8)	N(9)–Fe(1)–N(20)	83.45(6)
N(9)–Fe(1)–N(28)	97.16(8)	97.18(8)	N(9)–Fe(1)–N(27)	100.70(6)
N(9)–Fe(1)–N(33)	92.45(8)	93.04(8)	N(9)–Fe(1)–N(32)	95.58(6)
N(14)–Fe(1)–N(21)	92.18(8)	91.93(8)	N(14)–Fe(1)–N(20)	132.25(6)
N(14)–Fe(1)–N(28)	95.76(8)	95.37(8)	N(14)–Fe(1)–N(27)	91.80(6)
N(14)–Fe(1)–N(33)	94.92(8)	94.53(8)	N(14)–Fe(1)–N(32)	96.96(6)
N(21)–Fe(1)–N(28)	72.68(8)	72.79(8)	N(20)–Fe(1)–N(27)	72.20(6)
N(21)–Fe(1)–N(33)	73.31(8)	73.22(8)	N(20)–Fe(1)–N(32)	71.76(6)
N(28)–Fe(1)–N(33)	144.64(8)	144.83(8)	N(27)–Fe(1)–N(32)	138.18(6)
θ	86.86(2)	87.03(2)	θ	80.80(1)

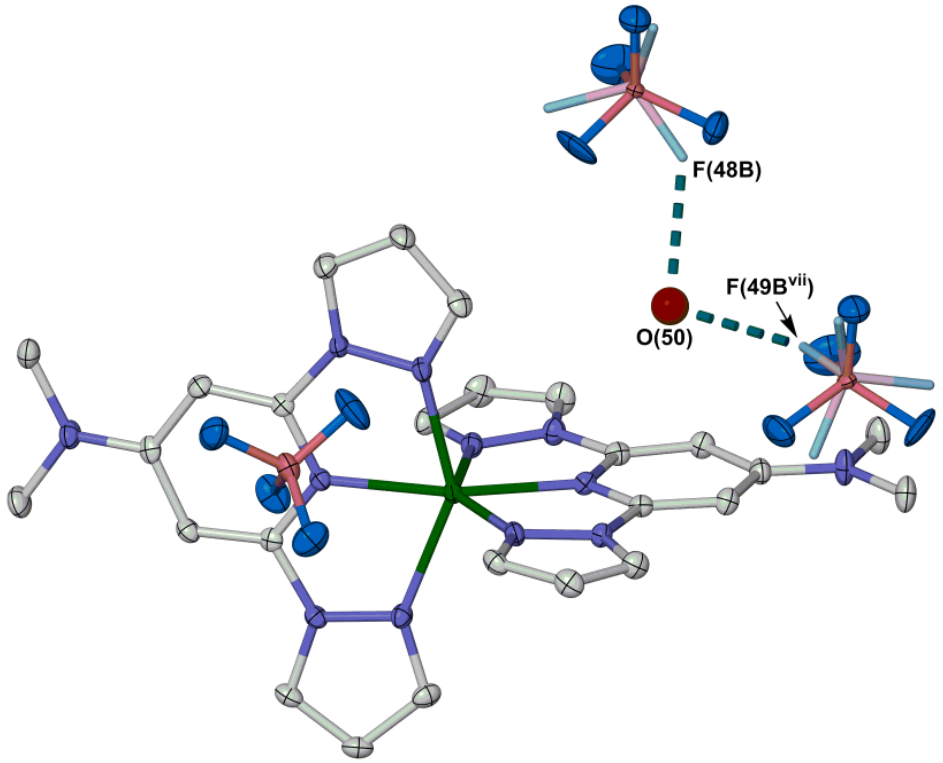

Fig. S16 View of the complete formula unit in $[Fe(bpp^{NMe2,H})_2][BF_4]_2 \cdot xH_2O$ (x = 0.29), showing the influence between the partial water site O(50) and the disordered anion. Thermal ellipsoids are at the 50% probability level except for the minor anion disorder site, which has arbitrary radii and paler coloration. All C-bound H atoms have been omitted for clarity. Symmetry code: (vii) ½+*x*, *y*, ½−*z*.

Color code: C, white; H, grey; B, pink; F, cyan; Fe, green; N, blue; O, red.

The marked hydrogen bond distances are O(50)…F(48B) = 2.40(2) and O(50)…F(49B^{vii}) = 2.50(2) Å.

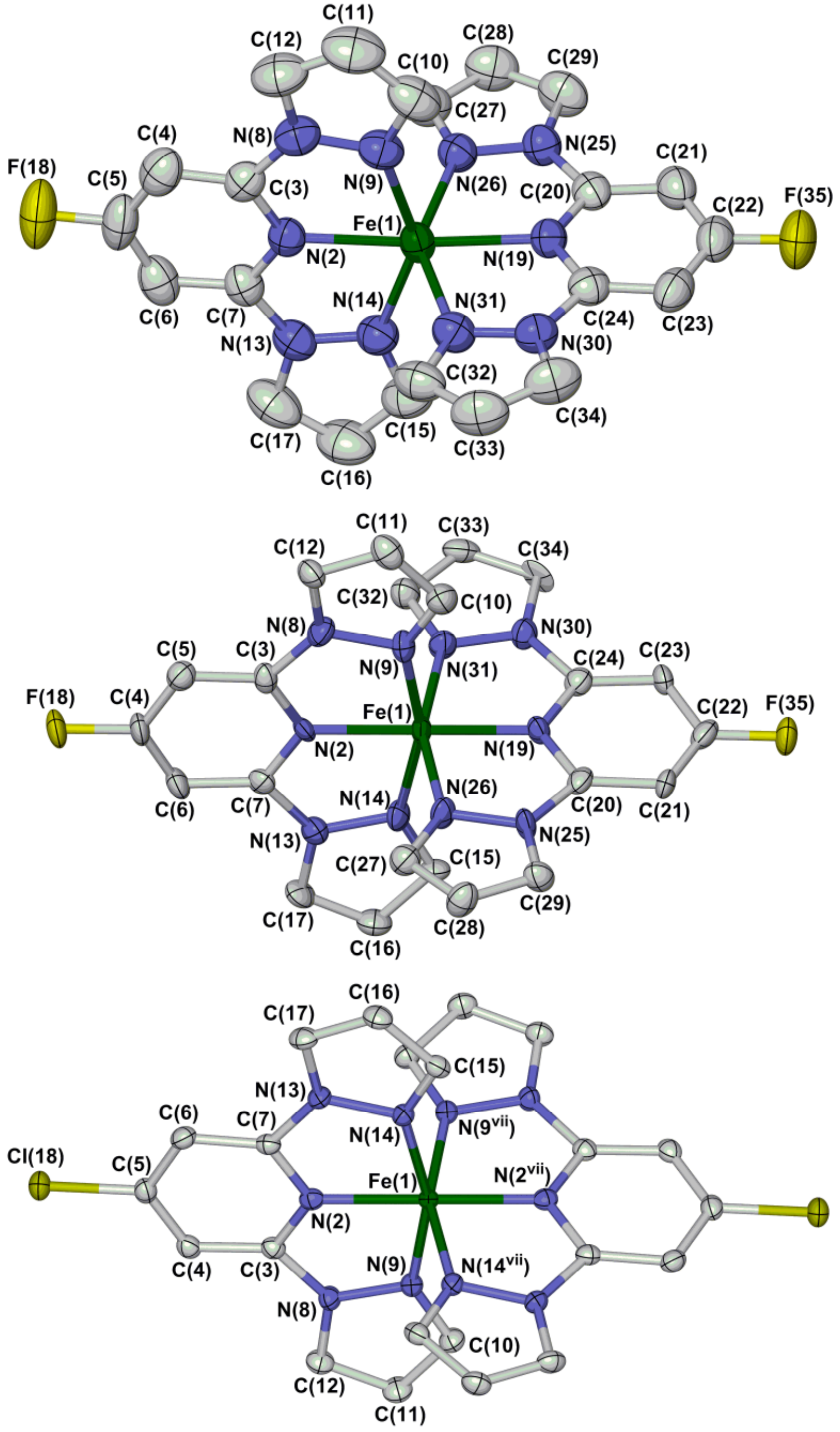

Fig. S17 View of the complex dications in $[Fe(bpp^{F,H})_2][BF_4]_2$ at 290 K (top) and 150 K (centre), and in $[Fe(bpp^{Cl,H})_2][BF_4]_2$ (bottom), showing the full atom numbering scheme. Atomic displacement ellipsoids are at the 50 % probability level and H atoms have been omitted for clarity.

Color code: C, white; F/Cl, yellow; Fe, green; N, blue.

Table S13 Selected bond lengths and angles in the crystal structures of the halogenated ligands (Å, º).[a] See Fig. S17 for the atom numbering schemes employed.

	$[Fe(bpp^{F,H})_2][BF_4]_2$		$[Fe(bpp^{Cl,H})_2][BF_4]_2$	
	290 K	150 K		
Fe(1)–N(2)	2.112(4)	1.904(7)	Fe(1)–N(2)	1.8982(19)
Fe(1)–N(9)	2.178(3)	1.964(8)	Fe(1)–N(9)	1.9661(19)
Fe(1)–N(14)	2.152(4)	1.984(8)	Fe(1)–N(14)	1.9822(18)
Fe(1)–N(19)	2.126(4)	1.904(7)		
Fe(1)–N(26)	2.191(3)	1.965(7)		
Fe(1)–N(31)	2.186(4)	1.996(8)		
N(2)–Fe(1)–N(9)	73.43(14)	80.2(3)	N(2)–Fe(1)–N(9)	79.98(7)
N(2)–Fe(1)–N(14)	73.61(14)	80.3(3)	N(2)–Fe(1)–N(14)	80.10(7)
N(2)–Fe(1)–N(19) (ϕ)	175.32(13)	179.3(3)	N(2)–Fe(1)–N(2^{iv}) (ϕ)	175.02(10)
N(2)–Fe(1)–N(26)	104.97(13)	99.2(3)	N(2)–Fe(1)–N(9^{iv})	96.57(7)
N(2)–Fe(1)–N(31)	107.94(13)	101.0(3)	N(2)–Fe(1)–N(14^{iv})	103.42(7)
N(9)–Fe(1)–N(14)	146.92(15)	160.5(3)	N(9)–Fe(1)–N(14)	160.00(7)
N(9)–Fe(1)–N(19)	102.08(13)	99.3(3)		
N(9)–Fe(1)–N(26)	95.32(13)	89.8(3)	N(9)–Fe(1)–N(9^{iv})	93.51(11)
N(9)–Fe(1)–N(31)	93.08(13)	92.9(3)	N(9)–Fe(1)–N(14^{iv})	90.57(7)
N(14)–Fe(1)–N(19)	110.94(13)	100.2(3)		
N(14)–Fe(1)–N(26)	95.80(13)	92.0(3)		
N(14)–Fe(1)–N(31)	94.28(14)	92.0(3)	N(14)–Fe(1)–N(14^{iv})	92.26(10)
N(19)–Fe(1)–N(26)	73.84(13)	80.3(3)		
N(19)–Fe(1)–N(31)	73.28(14)	79.5(3)		
N(26)–Fe(1)–N(31)	147.08(13)	159.8(3)		
θ	88.89(4)	89.15(7)	θ	87.30(1)

[a]Symmetry code: (iv) $-x$, y, $^3/_2-z$.

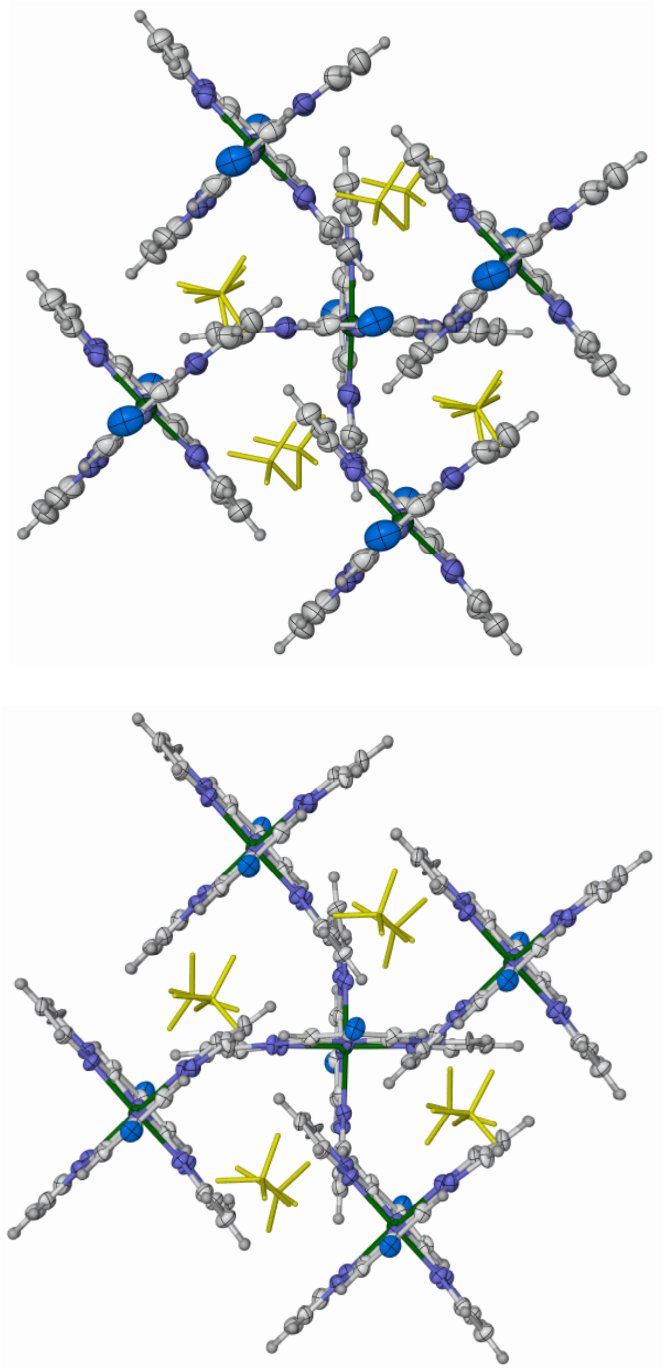

Fig. S18 Packing diagrams of $[Fe(bpp^{F,H})_2][BF_4]_2$ in its high-spin (top) and low-spin (bottom) states, showing the rearrangement of the lattice during the combined spin-transition and phase change.

Displacement ellipsoids are at the 50 % probability level except for the BF_4^- ions which are de-emphasized for clarity. Only one orientation of the disordered anion environment is shown. Both views are parallel to the [001] crystal vector.

Color code: C, white; H, pale grey; F{complex}, cyan; Fe, green; N, blue; BF_4^-, yellow.

These datasets were collected from different crystals, which were of opposite handedness. The low-temperature diagram is shown in the opposite of its true handedness, to allow comparison.

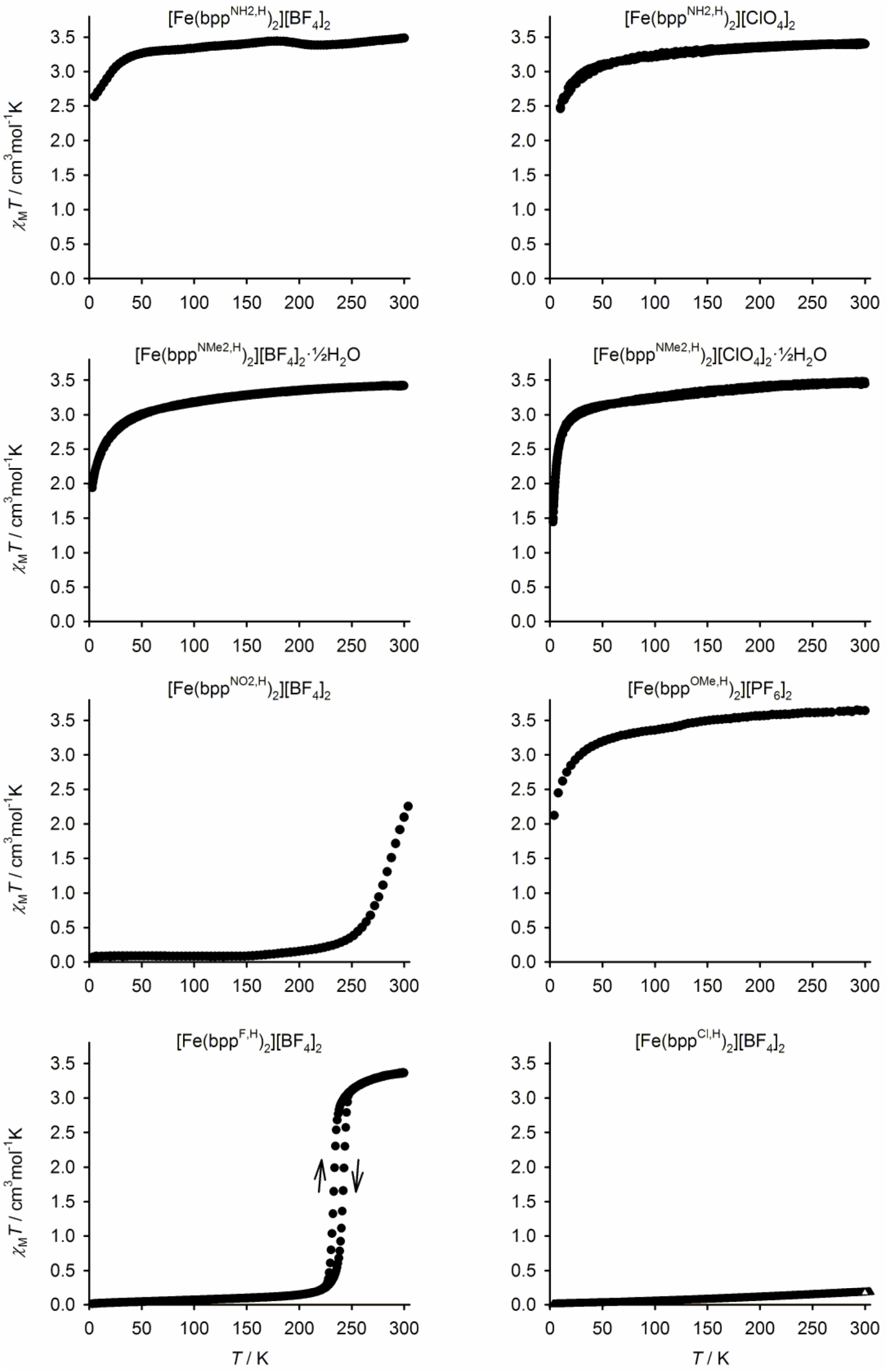

Fig. S19 Solid state magnetic susceptibility data for the new complex salts in this work (scan rate 5 Kmin^{-1}).

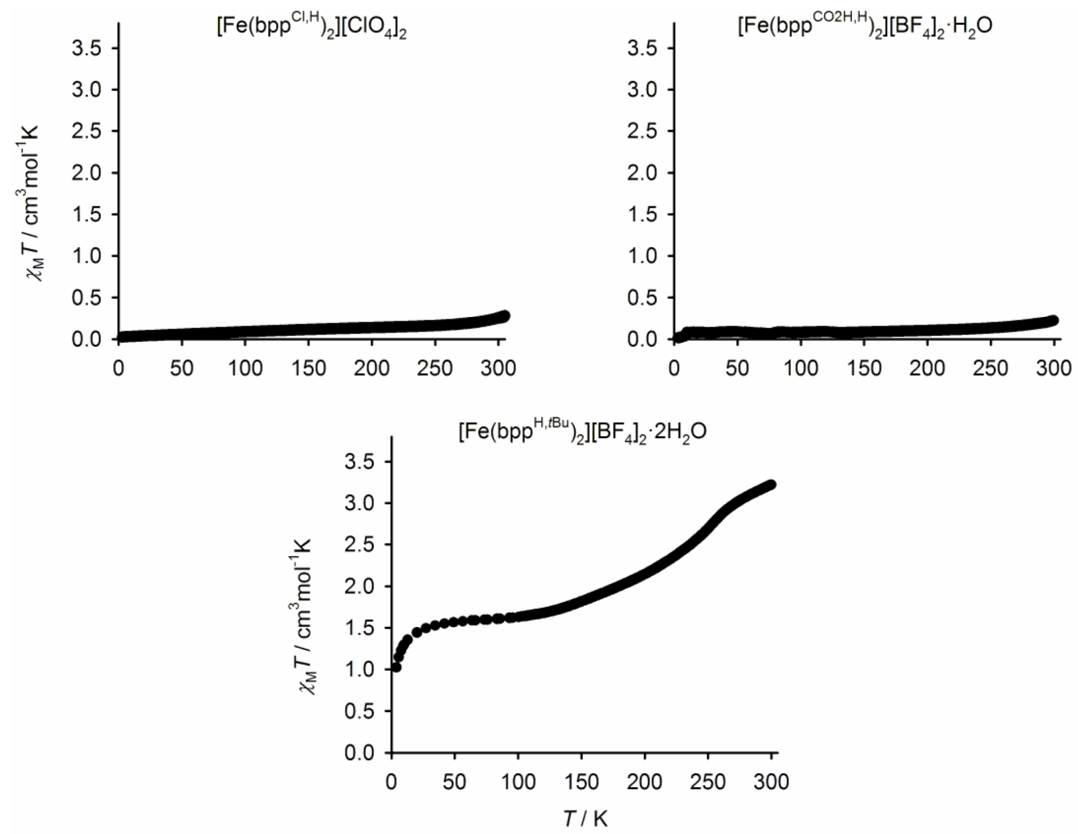

Fig. S19 continued.

The sample of $[Fe(bpp^{H,tBu})_2][BF_4]_2 \cdot 2H_2O$ is a poorly crystalline powder, that contains a mixture of high-spin and SCO-active material according to these data. The compound is almost fully high-spin at room temperature, but more detailed interpretation of its solid state properties is impossible at this stage since the compound was not obtained in crystalline form.

The spin-crossover temperatures from these data are listed in Table S9.
